# Inland surface waters in protected areas globally: Current coverage and 30-year trends

**DOI:** 10.1371/journal.pone.0210496

**Published:** 2019-01-17

**Authors:** Lucy Bastin, Noel Gorelick, Santiago Saura, Bastian Bertzky, Grégoire Dubois, Marie-Josée Fortin, Jean-Francois Pekel

**Affiliations:** 1 Joint Research Centre of the European Commission, Ispra (VA), Italy; 2 Department of Computer Science, Aston University, Birmingham, United Kingdom; 3 Google Inc., Zürich, Switzerland; 4 Department of Ecology and Evolutionary Biology, University of Toronto, Toronto, Ontario, Canada; Tanzania Fisheries Research Institute, UNITED REPUBLIC OF TANZANIA

## Abstract

Inland waters are unique ecosystems offering services and habitat resources upon which many species depend. Despite the importance of, and threats to, inland water, global assessments of protected area (PA) coverage and trends have focused on land habitats or have assessed land and inland waters together. We here provide the first assessment of the level of protection of inland open surface waters and their trends (1984–2015) within PAs for all countries, using a globally consistent, high-resolution (30 m) and validated dataset on permanent and seasonal surface waters based on Landsat images. Globally, 15% of inland surface waters are covered by PAs with mapped boundaries. Estimated inland water protection increases to 16.4% if PAs with reported area but delineated only as points are included as circular buffers. These coverage estimates slightly exceed the comparable figure for land but fall below the 17% goal of the Convention on Biological Diversity’s Aichi Target 11 for 2020. Protection levels are very uneven across countries, half of which do not yet meet the 17% target. The lowest coverage of surface water by PAs (<5%) was found in Africa and in parts of Asia. There was a global trend of permanent water losses and seasonal water gains within PAs, concomitant with an increase of both water types outside PAs. In 38% of countries, PAs lost over 5% of permanent water. Global protection targets for inland waters may well be met by 2020, but much stronger efforts are required to ensure their effective conservation, which will depend not only on sound PA governance and management but also on the sustainable use of water resources outside PAs. Given the pressures on water in a rapidly changing world, integrated management planning of water resources involving multiple sectors and entire basins is therefore necessary.

## Introduction

Protected areas (PAs) play a fundamental role in conserving genetic, species and ecosystem diversity, and in ensuring delivery of ecosystem services from natural habitats [1]. By protecting key habitats and associated species, PAs can contribute to averting biodiversity losses and meeting global conservation targets. The degree to which PA systems meet their declared goals, however, depends on whether they cover a sufficient proportion of natural habitats and on effective conservation management. For this reason, the international community agreed, in Aichi Target 11 of the Strategic Plan for Biodiversity 2011–2020 of the Convention on Biological Diversity (CBD), to conserve by 2020 at least 17% of terrestrial and inland water areas through effectively and equitably managed, ecologically representative and well-connected systems of PAs [2]. In this target, particular attention is given to inland waters, encompassing a wide range of water-influenced environments [3].

Many surface waters and wetlands are unique ecosystems upon which numerous plant and animal species depend [4], and can provide key ecosystem services such as nutrient cycling, primary production, water provisioning, water purification and recreation [5–7]. Surface waters may be at particular risk due to pressures such as unsustainable consumption, wetland drainage, land use intensification, stream diversion and climate change, especially in dry areas where water scarcity is becoming a major limiting factor for wildlife and humans [6, 8–9]. For these reasons, extinction risk for freshwater species has been found to be higher than for their terrestrial counterparts [10].

Despite the importance of, and threats to, surface water, global assessments of PA coverage and management effectiveness have primarily focused on land habitats (e.g., forest), or assessed terrestrial and water habitats as one [11–13]. Wetland decline globally and within RAMSAR regions has recently been estimated from literature review [14]. However, detailed assessment covering large contiguous areas has historically been challenging, particularly at regional to global scales, because inland waters cover a small proportion of the earth, and are frequently distributed in many small water bodies. This makes it difficult to specifically identify surface waters and wetlands, despite their disproportionate importance for ecosystem functioning. In addition, water coverage is strongly dynamic, seasonally and inter-annually, which restricts the representativity of the information that may be obtained at any single date.

Here we present the first global assessment of the areal protection coverage of inland open surface waters and their trends for all countries, considering both permanent and seasonal water. For this purpose, we use the unprecedented spatial (30 m) and temporal resolution (seasonally and yearly) of a recent, globally consistent and validated data set, the Global Surface Water Explorer (GSWE), which was derived from the analysis of three million Landsat satellite images from 1984 to 2015 [15]. First, we evaluate the spatial extent of current surface water protection, i.e. how much of surface water, as mapped in 2015, is covered by formally designated PAs. Second, we assess net changes (1984–2015) to areal extent of water bodies within PAs to track perturbations in one of the most precious and threatened habitat resources on Earth, and compare these changes to those observed, over the same period, in the unprotected portion of the surface water in the same countries, to provide an informative benchmark for the significance of these changes. Our analysis focuses, as does the GSWE, on open inland surface waters, hereafter referred, for brevity, as surface water. This excludes many water bodies in swamps and flooded forests, bogs, fens and mires that are usually encompassed under the CBD definition of “inland water” [16] but that are not easy to detect and reliably map from optical remote sensors [15]. While the global extent of inland and coastal wetland areas has previously been estimated at 530–1,620 million ha (S1 Appendix), our analysis of open inland surface waters covers approximately 359 million ha of permanent and seasonal water bodies[15].

## Materials and methods

### Inland surface water spatial and temporal data

The Global Surface Water Explorer (GSWE) uses the full 32-year history of the entire multi-temporal orthorectified Landsat 5, 7 and 8 archive between 1984 and 2015 to map the spatial and temporal variability of global open surface water and its long-term changes [15], adapting a Hue-Saturation-Value (HSV) transformation method first described in [17]. Each pixel in 1,823 terabytes of Landsat data was classified as open water (defined as any stretch of water larger than 30 m by 30 m open to the sky, including fresh and saltwater), as land or as a non-valid observation using an expert system [15]. Classification performance was measured using over 40,000 reference points; the classifier produces less than 1% of false water detections, and misses less than 5% of water [15]. Further details of this validation are given in S2 Appendix. GSWE also describes the intra-annual distribution of water, discriminating between permanent (underwater throughout the year) and seasonal (underwater for less than 12 months of the year) water surfaces [15]. The GSWE shows that during the period from 1984 to 2015 just over three per cent of the Earth’s landmass was under water at some time [15]. It must be noted that some classes of surface water features are not considered in this global assessment. Optical remote sensing can only detect land covers which are directly visible from above; this excludes surface water which is obscured by floating, overhanging or standing vegetation cover, such as swamp forests, or water which is beneath infrastructure such as tunnels and bridges. In addition, given the fixed granularity of the historic satellite data, bodies of water smaller than 30 m by 30 m will remain undetected at past epochs. Rivers will be detected where they are wide enough to dominate at least one pixel, and where the water is open to the sky. Narrow and heavily-forested river segments are often undetected at least for some parts of the year.

### Protected areas: Data source and processing

To assess the coverage of water bodies by PAs, we used the April 2016 version of the World Database on Protected Areas (WDPA) [18]. WDPA includes all sites designated at a national level (e.g. national parks), under regional agreements (e.g. the Natura 2000 network in the European Union) and under international conventions and agreements (e.g. natural World Heritage sites). As in other global PA assessments (see e.g. [19–21]), we excluded from analysis those PAs with a “proposed” or “not reported” status, sites reported as points without an associated reported area, and UNESCO Man and the Biosphere Reserves (as their buffer areas and transition zones may not meet the IUCN PA definition, and because most of their core areas overlap with other PAs). We assessed PA coverage in two ways: first, considering only PAs which have a polygon geometry in the WDPA; second, including also the PAs provided as point locations with a reported area (about 5% of the world’s PAs). For these points, we generated a circular buffer matching the reported area, as is common practice in similar global analyses [19–22]. In both cases, all PA polygons (including the buffered points in the second case) were dissolved to remove all overlaps between different designation types and avoid double counting (e.g. where the same area is designated both as a national park and as a World Heritage site). For a complete workflow description of processing details, see S3 Appendix. Unless otherwise noted, the coverage results presented below refer to the case in which only the PAs with a polygon geometry in the WDPA have been considered. S4 Appendix gives more detail about the location and size of PAs which only have point geometry, and investigates the effect of this data limitation and of the buffering strategy.

### Assessment of changes in surface water

We summarized transitions between three classes (permanent water, seasonal water, no water) from 1984 to 2015 as discriminated from intra-annual water distribution patterns by our colleagues [15]. A full description of the treatment of water occurrence, recurrence and seasonality may be found in that paper. For this analysis, we quantified transitions between the first year in which representative observations were acquired and the last year of observation. Representative years are identified by comparing each year in turn with the annual pattern of monthly recurrence from the temporal profiles (i.e. availability of Landsat images processed in the GSWE). These profiles identify months in which water was observed, and indicate the percentage of valid observations classified as water in any given month. A year is flagged as representative if it contains sufficient valid observations from any combination of months to bring confidence to the determination of the presence or absence of water. Years where unobserved data exceeded 5% were excluded from the analysis. Given that transitions are detected based on the first and last available year of reliable data, and that the available data history is shorter than 32 years for some regions, there may be some differences in the actual period of change being considered in each particular location (discussed fully in [15]). S2 Appendix gives a full description of the change detection method and the spatial distributions of validation points, misclassifications and invalid observations. While it might be expected that detection accuracy would vary with latitude and aridity, in fact there is not a clear correlation which can be exploited to estimate the reliability of results based on these factors (see S2 Appendix for full discussion).

We quantified net change including transitions between categories: for example, areas which transitioned from permanent to seasonal water are counted in the net loss of permanent water and in the net gain of seasonal water. When classifying countries by their net change of permanent surface water, we applied a leeway of 5% to allow for normal inter-annual variability; for seasonal water trends, and for seasonal and permanent surface water combined, a 10% leeway was applied. The latter accounts for higher inter-annual variability and more challenging detectability of seasonal water from the reduced number of cloud-free satellite images available over shorter seasonal periods. The rationale for, and effect of, these threshold levels are explained in more detail in S2 Appendix.

Changes were assessed within and outside PAs in each country. Buffered point PAs may give misleading results in such a comparative analysis because of the uncertainty of PA boundaries relative to the high-resolution mapped water changes. However, it can be argued that a circular buffer is better than nothing, since it represents the fact that some protection exists at a location, and may capture at least some of the protected habitats, albeit very roughly. In the following discussions, results are given for the analysis excluding point buffers (unless explicitly stated otherwise). As a supplement, S4 Appendix and S3 Table present the results of including buffered point PAs on national surface water trends.

### Country layer

We conducted all analyses at the country level, both for the PA coverage of inland surface waters and for the surface water trends from 1984 to 2015. For this purpose, we considered, for each country, the PAs and inland surface water that occurred within the country boundaries as mapped in version 2.6 of the Global Administrative Areas dataset (GADM) for year 2012 [23]. 238 of the countries differentiated by this dataset contained pixels identified as inland surface water, and these are listed in all supplementary Tables. It is important to note that on occasion, there may be mismatches between a PA boundary which follows a national border and that same border as mapped in GADM. This results from the varying basemaps and digitising strategies of the countries which contribute data to WDPA, and it means that, very occasionally, small areas of a PA within a country will apparently ‘spill over’ into another country. This affects a very small proportion of the overall protected area. Global results have been derived from these country-level values by directly aggregating the areas computed–i.e., global percentage of permanent water under protection is computed using the total area of permanent inland surface water and the total area of *protected* permanent surface water across all countries.

## Results

### Coverage of inland surface waters by PAs

Globally, 15.0% of currently (as of 2015) existing open surface waters (permanent and seasonal combined) are included in PAs whose boundaries are mapped in the WDPA. Global protection is higher for seasonal water (17.8%) than permanent water (14.1%). If we include circular buffers around point PAs, the estimated coverage of surface waters by PAs increases to 16.4% (19.8% and 15.4% for seasonal and permanent waters, respectively). These numbers all exceed the 14.7% of protection for land and inland water combined, as estimated in recent assessments which also included point PA buffers in their coverage statistics [19–21], and are close to the 17% of Aichi Target 11. Because of the poor spatial representation of actual PA geometry by these circular buffers, all statistics which follow exclude point-only PAs, unless otherwise explicitly stated.

**Fig 1:**
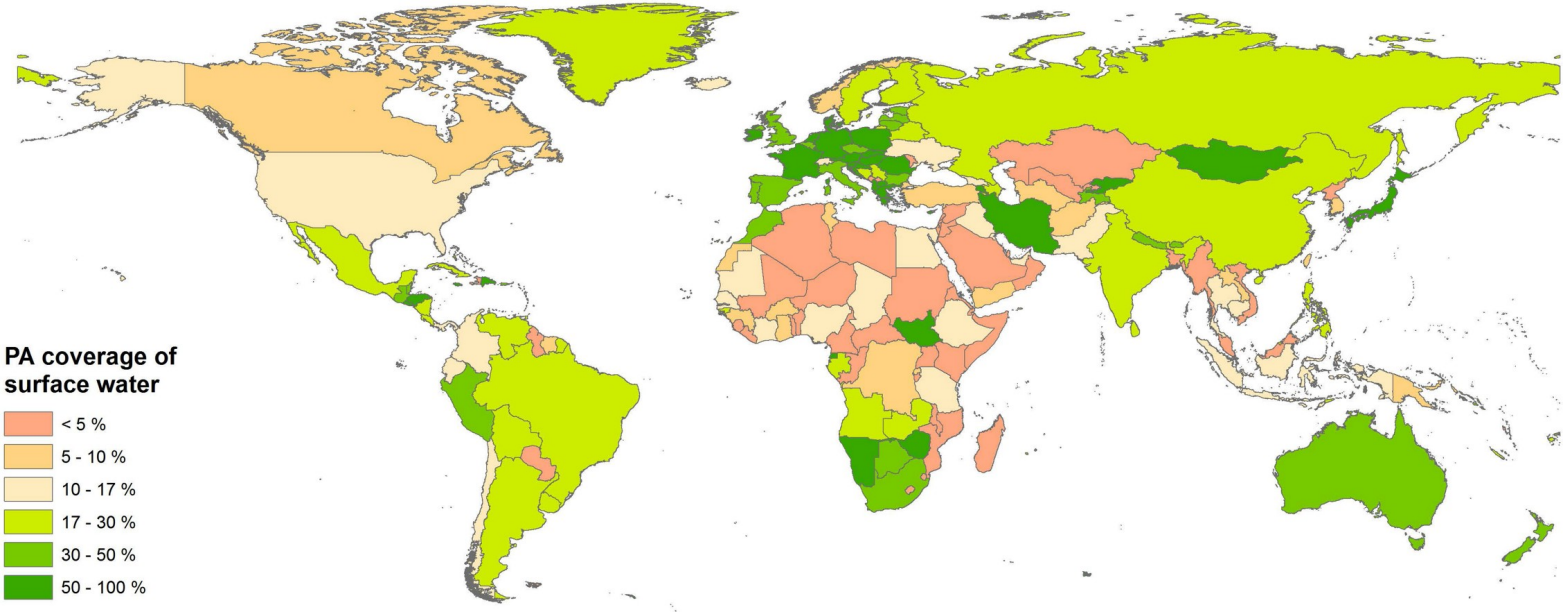
Coverage of surface waters (both seasonal and permanent) by protected areas in all countries.

Despite the encouraging level of protection at an aggregate global level, water coverage by PAs is quite uneven when compared between countries (Fig 1). The lowest surface water protection (<10%) is found in most of Africa, parts of Asia, Canada and several other smaller countries, while the highest (>30%) is found in Europe, Southern Africa, Mongolia, Iran, Australia, New Zealand, Peru and other smaller countries. 53% of countries (representing 45% of the world’s land) still have surface water protection far below the 17% of Aichi Target 11 (see S1 Table).

**Fig 2:**
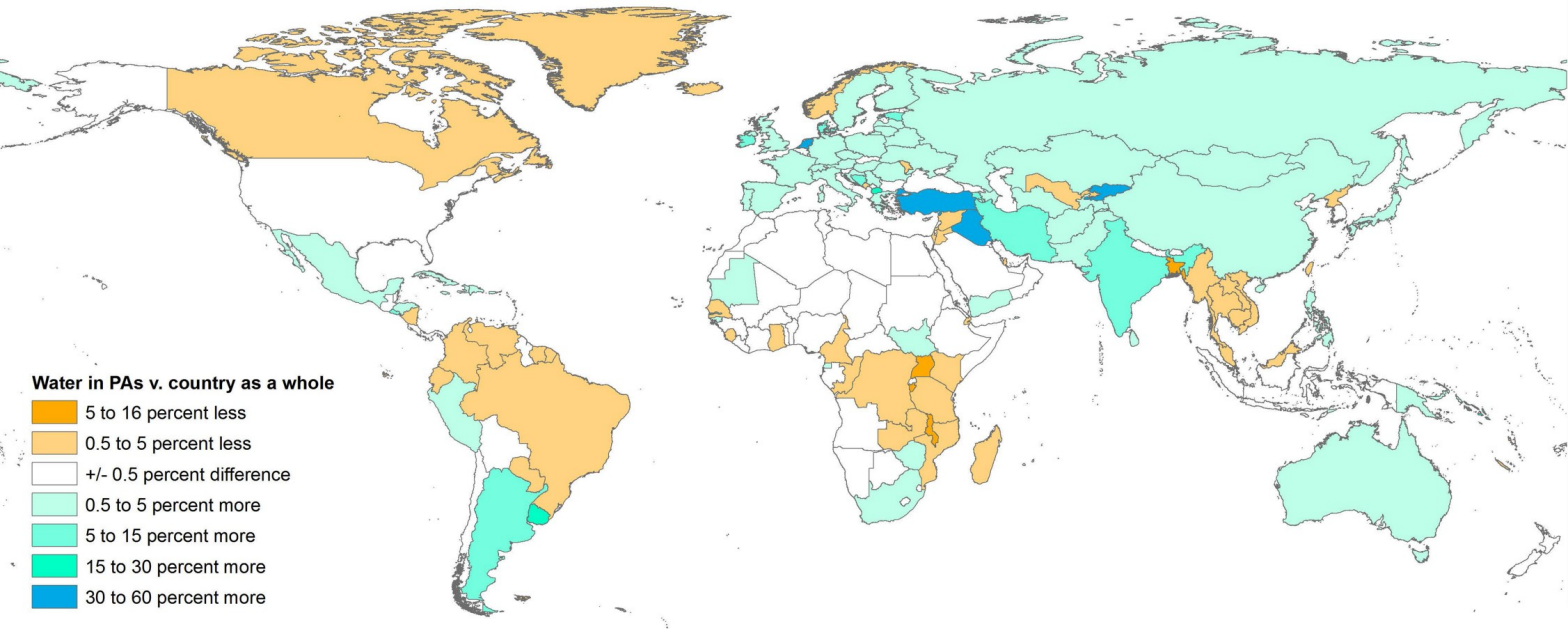
Representativity of water protection, by country. Difference between the percentage of a country's area covered by surface waters in 2015 and the percentage area of that country's PAs which is covered by surface waters. Positive values (bluish colours) indicate that surface waters are represented in PAs more often than would be expected by chance (i.e. that the PA system preferentially covers surface water bodies).

In about half of countries (51%), the percentage of PAs’ area occupied by surface waters is above the percentage of water over the entire country (Fig 2)–i.e., PAs preferentially cover surface water rather than land habitats. However, this is balanced by other countries where this relative representation of water habitats in PAs is quite low, such as Canada, most of tropical South America, or some countries in Africa or South-Eastern Asia (Fig 2). Consequently, open surface waters (both permanent and seasonal waters) currently occupy, as of 2015, 2.66% of the surface of the world’s PAs–comparable to their 2.68% share of the world’s total land area.

### Permanent water trends in PAs

**Fig 3:**
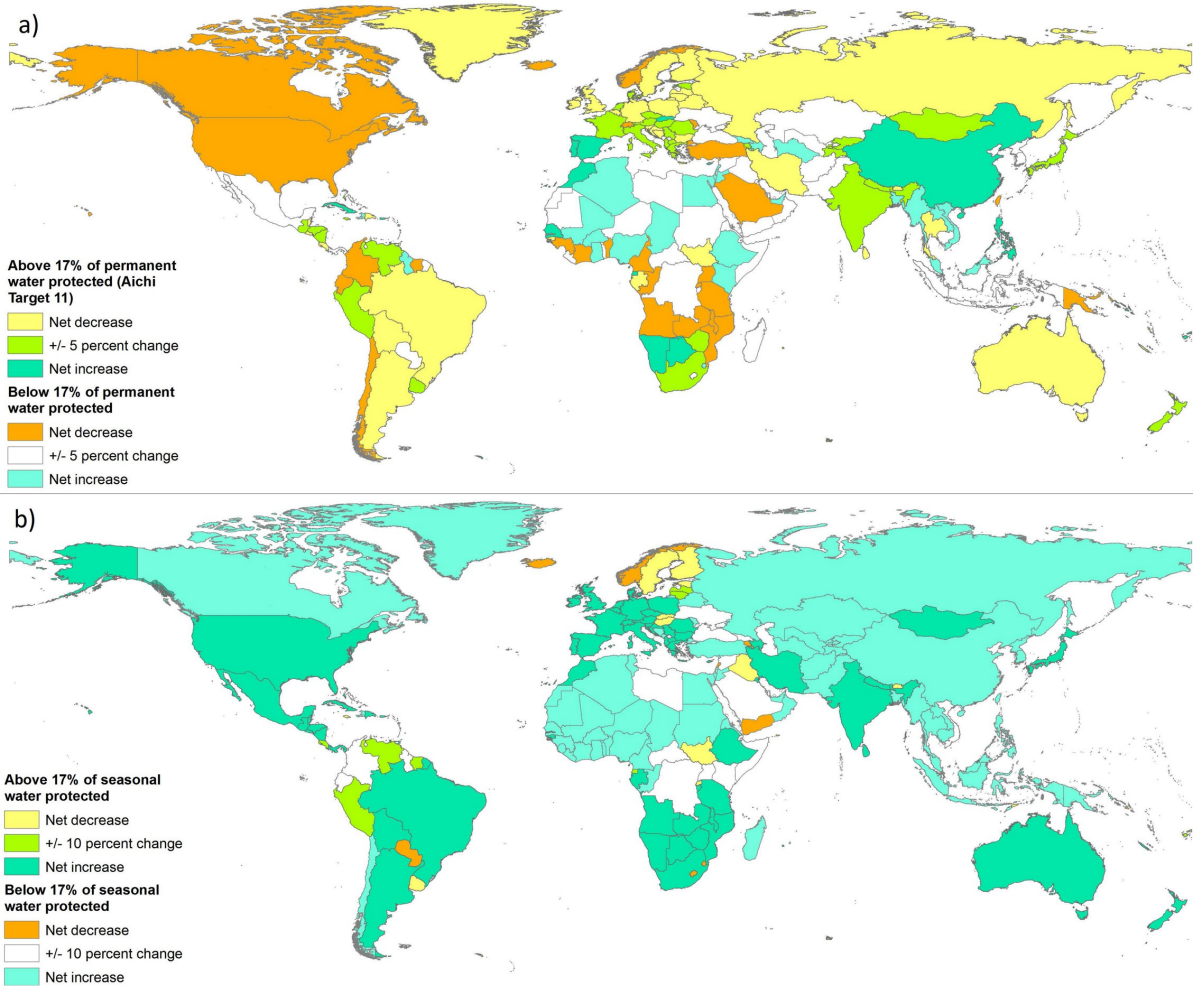
Water trends within protected areas, 1984–2015. Surface water trends (increasing or decreasing in the period 1984–2015) within current PAs and surface water coverage by current PAs (above or below the 17% coverage specified in Aichi Target 11) separately assessed for permanent water (a) and seasonal water (b) in all countries. ‘Stable’ thresholds are +/- 5% for permanent water, and +/- 10% for seasonal / all water.

**Fig 4:**
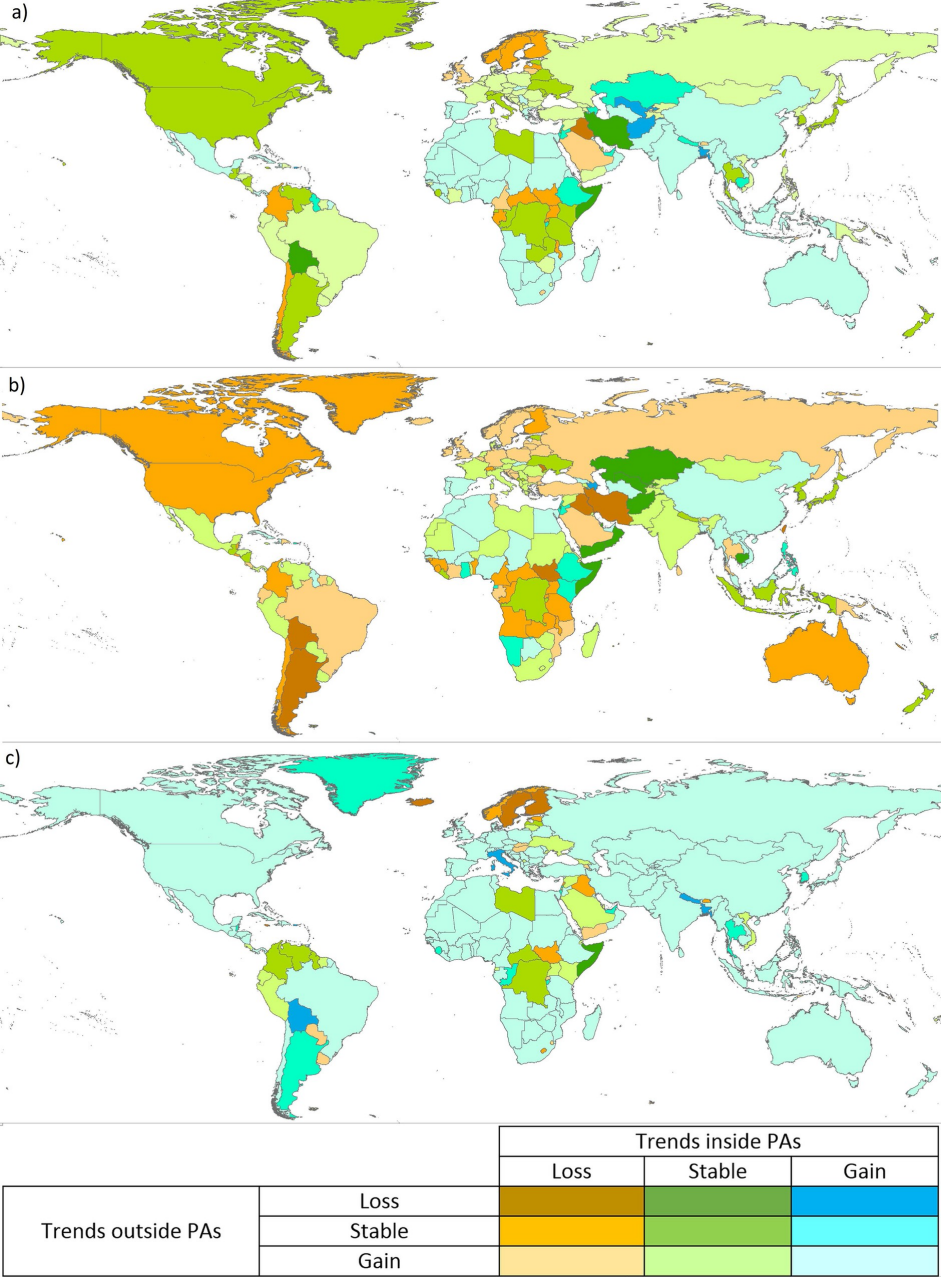
Net changes in water surface within and outside PAs, 1984–2015. Changes are assessed for (a) permanent water (b) seasonal water and (c) all surface water in the period 1984–2015 for all countries. ‘Stable’ thresholds are +/- 5% for permanent water, and +/- 10% for seasonal / all water.

In more than a third of countries (38.2%) there was a net decrease in permanent water surface within PAs (with the 5% threshold), mainly in North and South America, Northern Europe, Russia, Australia and other countries in Africa and Asia (Fig 3A). In many of these countries (16.0% compared to 38.2%) the net decrease within PAs contrasted with an increase in permanent water outside PAs; for example in Sweden, Turkey and Sri Lanka (Fig 4A). A similar number of countries (15.9% of all countries) had a net decrease in permanent waters inside PAs but a stable trend outside, most noticeably Zambia, Uganda, Tanzania, and the United States (Fig 4A). Only a few countries (6.3%), including South Sudan, Bolivia, Iran and Iraq, presented net permanent water losses both inside and outside PAs (Fig 4A).

Net gains in permanent water within PAs occurred in 19.7% of countries (Fig 3A), i.e. much less frequently than net decreases (38.2% of countries). When permanent water increased in PAs, it was usually accompanied by a net increase in permanent water in the unprotected landscapes (13.9% of countries). Less frequently, permanent water gains inside PAs coincided with a static situation outside (4.6% of countries) or with losses of permanent water outside PAs (only two countries). For summaries of net areal change at an individual country level, see S2 Table, and for an aggregation of trends, see S3 Table.

### Seasonal water trends in PAs

Seasonal waters were more dynamic within PAs than permanent waters, and seasonal water dynamics were more pronounced within than outside PAs. The global sum of absolute net area change per country was 52% higher for seasonal than for permanent waters inside PAs, but only 18% higher outside PAs.

Most seasonal water changes inside PAs were gains (80% of the 183 countries with more than 10% net change, Fig 3B). This was generally accompanied by a similar pattern outside PAs (Fig 4B). Only a few countries (8.4%) gained seasonal water within PAs when seasonal water was being lost or remaining relatively static outside; most notably Bolivia, Bangladesh and Argentina (Fig 4B). About 15% of countries lost seasonal waters within PAs, quite evenly split between three subcases: loss outside PAs (such as Finland and Sweden), gains outside PAs (including Paraguay, Uruguay, Yemen, Swaziland, Rwanda and Armenia) or no trend outside PAs (including Lesotho, Estonia, Lebanon, Iraq and South Sudan).

### Conversion from permanent to seasonal waters and overall inland surface water trends

**Fig 5:**
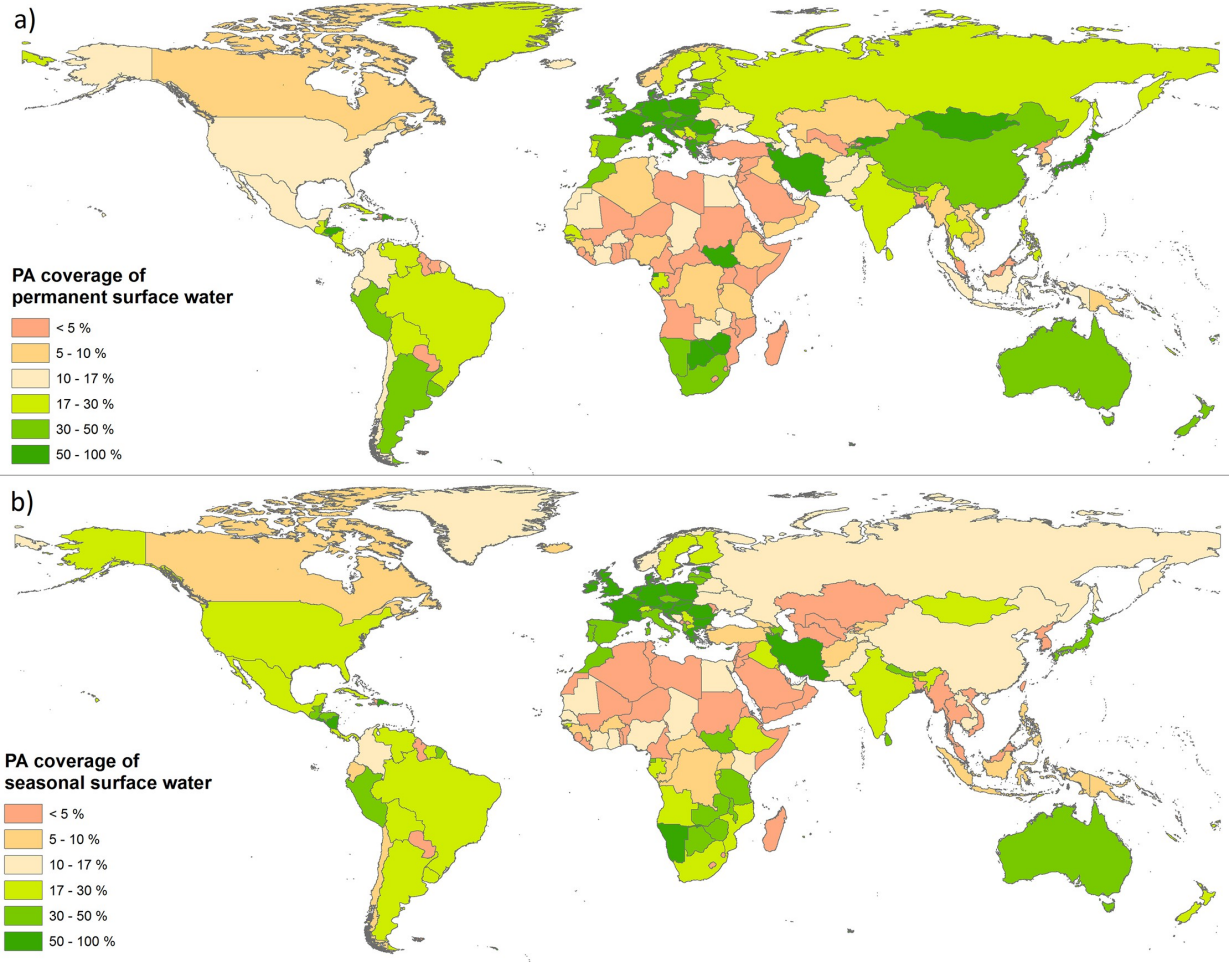
Protected area coverage of (a) permanent surface water and (b) seasonal surface water, by country.

The pattern worldwide shows a general shift from permanent to seasonal water in many PAs over the 32-year period (Fig 3A and Fig 3B). There is a dominant tendency for PAs to lose permanent water and gain seasonal water, giving the overall impression of a global increase in the total area of surface water within PAs (Fig 4C). This aggregated picture may mask important changes for conservation monitoring and planning, since seasonal and permanent water bodies fulfil some very different ecological functions and cannot be considered interchangeably. The GSWE’s ability to distinguish seasonal water over a long time period is therefore of huge potential value in a conservation and water management context. A global picture of current protection levels for seasonal and permanent water can be seen in Fig 5.

About half of countries had net change in all inland surface waters within PAs (for the 10% threshold considered). Gains were more common than losses, occurring in about two thirds of the countries with net change (Fig 4C). In countries like Bolivia, Somalia and Iran, surface water was lost countrywide, but remained relatively stable within PAs (Fig 4C). In some countries, including Afghanistan, Bangladesh, Puerto Rico and Uzbekistan, water within PAs increased despite losses in unprotected lands. Conversely, losses within PAs, despite neutral or positive trends outside are reported in Chile, Colombia, Fenno-Scandinavia, Iraq and several Central and East African countries (Fig 4C).

## Discussion

### Surface water coverage by PAs is close to Aichi Target 11 globally, but not in many countries

Aichi Target 11 specifies a 17% protection target for terrestrial and inland water areas for year 2020. Considering inland surface waters as a separate subset (see [3], sections 10c, 10d), this target is already met for seasonal, but not for permanent surface water. Present protection estimates for permanent and seasonal surface water combined are 15.0% considering only polygon PAs, and 16.4% when buffered point PAs are included. The latter may underestimate actual coverage, since any PAs designated for wetland habitats would preferentially follow rivers and other water bodies, potentially bringing the global protection level closer to the emblematic 17% threshold. The current global coverage provided by all PAs (polygons and buffered points) to open water habitats is better than that for the terrestrial realm in general (14.7%; [20–21]). Considering that more PAs are likely to be designated before 2020, and given the growing recognition of the importance of many water bodies and wetlands for biodiversity and PA conservation goals, a global 17% target has a good chance of being achieved by 2020, at least when seasonal and permanent waters are considered together.

The situation is, however, quite uneven across the world, with low and worrying levels of protection in some countries or regions, such as in most of Africa, parts of Asia and Canada, although there is mounting evidence for the benefits of effective protection for freshwater ecosystems and species. In South Africa, for example, it has been shown that rivers within PAs tend to be a lot more intact than outside PAs [24], while several PAs along the Tanzanian shores of Lake Tanganyika effectively contribute to the conservation of freshwater fish diversity despite their largely terrestrial focus [25]. It is, therefore, important to expand and reinforce the PA system in those countries with low surface water protection, preferentially focusing on water bodies of conservation importance, to safeguard the rich biodiversity and the delivery of ecosystem services associated with aquatic habitats [26–28].

### The importance of accurate PA delineation to assess aquatic habitat protection

For a number of countries, including Niger, Algeria and Democratic Republic of Congo, the impact of including buffered point PAs was very significant, even though these are not the countries with the highest numbers of point-only PA records. The additional estimated percentage of protection contributed by these buffered point features is provided in S1 Table (last column). Given that open water habitats are in many cases dendritic and elongated, unknown or inaccurate PA boundaries can have a particularly significant effect. When point PAs are included in the analysis, a few countries show switches in their estimated trends: for example, Uzbekistan moves from a neutral to a positive trend for permanent water inside PAs despite general loss outside, due to a reported protection of over 5 thousand square kilometres in the Aydar-Arnasay Lakes. Likewise, Mozambique moves from a negative to neutral trend within PAs, largely due to the inclusion of the Marromeu Complex of the Zambezi Delta (a delineated polygon for this PA has been added to the WDPA since the analysis described here). S4 Appendix explores this issue in more detail and illustrates the impact of the point-only data at country level, and for a few selected examples.

We therefore stress the importance of consistently-mapped PA polygons for accurate and comparable reporting of countries’ efforts towards conserving their habitat resources [29]. Data completeness is being improved through systematic efforts by each of the countries providing information to the WDPA [18], and the constant efforts of UNEP-WCMC to improve this database mean that more accurate assessments will be possible in the future.

### Coverage by PAs does not necessarily translate into maintenance of surface waters

Although global coverage of surface waters by PAs was close to the 17% goal of Aichi Target, protection success should not be assessed on percentage area alone [30–31]. PA coverage does not always translate into effective conservation [31–33], and the within-PA water transitions reported here demonstrate that protection alone does not always ensure steady hydrological cycles or buffer against prevailing effects of extraction outside. Hence, management approaches specifically targeted towards the conservation of important water bodies and wetlands are needed, especially given the pressures on water resources in a rapidly-changing world [34–36].

Changes in water availability (and thus in biodiversity) within PAs also depend heavily on management outside, particularly extraction of water for agriculture, industry, urbanization, human consumption and dams whose upstream and downstream impacts are felt at the watershed level [11, 37–38]. Therefore, the changes reported above reflect changes in hydrological regimes and water use both inside and outside PAs. For example, the construction of dams, which usually happens outside PAs, may reduce, due to water flow regulation, the extent of permanent water in lakes or river sectors in the PAs located downstream [39]. Negative transitions due to agricultural irrigation will be especially accentuated by temperature increase and precipitation decrease resulting from climate change [40]. However, some surface water loss within PAs may also be deliberate, for example where dams or artificial lakes are being removed or rehabilitated [41]. It should also be remembered that the currently available GSWE detects changes in surface area which can be used to estimate changes in flux and volume [16], but cannot reliably evaluate the quality of water available to humans and wildlife. Issues such as salinization and pollution must therefore be assessed in addition to the trends reported in this work.

### Caveats and further research

The analysis presented here represents a baseline workflow for assessing and monitoring trends in the world’s surface water within and outside PAs, and inevitably suffers from deficiencies in the available data. The most critical gaps are limits in the accuracy of PA mapping and, to a lesser extent, in the resolving power and coverage of the sensing satellites. We here discuss practical strategies to overcome these challenges, or at least to quantify their impact, so that future analyses can yield detailed recommendations for countries wishing to incorporate representative protection of water habitats into their planning for Aichi Target 11 or other post-2020 targets.

We treated current PA boundaries as constant throughout the study period because the WDPA does not explicitly track changes in PA boundaries. Thus our study presents a summary of surface water changes in those areas that are currently recorded as ‘protected’. Furthermore, we did not separately consider potentially important PA characteristics such as protection regime (whether they are strict reserves or open to sustainable use of resources), governance / management types, and the year of PA designation, which may fall after the start of the temporal window of the water trend dataset. Dedicated analysis at the individual PA level will improve our understanding of the interactions between different types of protection, the period for which protection has been in place, and the status of open water habitats through time. PA-level information is already available, for PAs larger than 25 km^2^, in version 3.0 of the DOPA Explorer (released in November 2018), one of the key applications of the Digital Observatory for Protected Areas (DOPA) of the Joint Research Centre of the European Commission [42]. The PA-level statistics available through this platform are regularly updated (at the time of writing, they are based on the July 2018 version of the WDPA) and thus they benefit from the constant improvements in PA mapping. While the WDPA is the primary authoritative global resource for mapping protection, it currently omits some categories of management which may effectively confer high ecological protection on a site, known as ‘other effective area-based conservation measures’ (OECMs). Conversely, some of the PAs in the global database are effectively ‘paper parks’ where a lack of management on the ground can lead to degradation, deforestation and other destructive processes. On occasion, deliberate conservation planning actions may lead to the degazettement of a site, and there can be a time lag before this information is submitted to the WDPA. For all these reasons, it will be critical to closely follow the implementation of the recently-drafted OECM reporting guidelines [43], and to make use of complementary sources of information such as PADDDTracker [44]. PADDDtracker is a WWF-led initiative which collates information on downsizing, downgrading and degazettement of protected areas, and which captures some information on OECMs that may not currently be submitted to the WDPA. The data currently available from PADDDtracker is at present rather patchy and not sufficiently complete for a global study. However, there are a number of regions where an aggregation of the WDPA with PADDDtracker data can yield a more complete picture of current protection.

The area of water bodies was identified as an Essential Climate Variable (ECV) by the Global Climate Observing System (GCOS) and its monitoring by remote sensing has therefore been the subject of much research. The GSWE [16] has been adopted by UN Environment -the custodian agency for Sustainable Development Goal (SDG) 6.6—as one of the official indicators for SDG 6.6.1 (Change in the extent of water-related ecosystems over time). Despite the quality and scope of the GSWE, some biases still remain: for example, water under forest canopy remains undetected, and the current 30-metre resolution is too coarse for detection of small rivers, streams and ponds. Note also that a number of the countries of the world are islands or have highly-dynamic coastlines. This fact, combined with currently-designated PAs in coastal areas, can mean that observed ‘loss’ and ‘gain’ in surface water also captures some coastal erosion and deposition over the 32 years considered. Mobile PAs have been proposed [45–46], especially in the marine context, and may also be appropriate for such coastal habitats, depending on the original reasons for designation. Finally, for some spatio-temporal windows, the Landsat archive is restricted by cloud cover or for other reasons, and transitions were only detected between the first and last years of available valid acquisitions. However, the retrieval by the Landsat Global Archive Consolidation Project of c. 750,000 historic images will fill some temporal gaps, allowing future versions of the GSWE to more accurately reflect historic water distribution. Future remote sensing image acquisitions will also be available from Landsat, and from the new contributions from the satellites Sentinel 1 and Sentinel 2 from the European Copernicus earth observation programme which will also offer additional radar and optical satellite imagery. This will help to improve the detail and accuracy of the information derived through active fusion of the Copernicus instruments’ higher radiometric and spatial resolution with Landsat’s thermal band and overlapping spectral acquisitions. This will allow the development of an improved monitoring service to go beyond 2015 and approach near real-time information (see e.g. [47]).

Further related work is in progress to derive the full temporal distribution of surface water for individual PAs, and to distinguish features such as dams and artificial or rehabilitated lakes inside PAs. Such further work is planned as part of the development of, and of the information to be integrated into, the DOPA. These planned developments will in future allow users to select customized periods of interest for assessing surface water changes on individual PAs, beyond the fixed period 1984–2015 considered in this study and in the current DOPA Explorer 3.0. Customizing the analysed period in this way will allow the contribution of inter-annual climatic variability, such as drought or rainy years, to be distinguished in the reported trends. It will also allow quantification of the minimum and maximum extents of surface water within a focal period of local ecological interest, rather than just the trends from the initial to the last available year in the global dataset.

## Conclusions

Global targets on PA coverage of inland waters may well be met for open surface waters by 2020. Dedicated and much stronger efforts are however required to achieve effective conservation of formally protected water resources, given the trends observed and the multiple pressures to which inland waters are exposed in many parts of the world. The effective conservation of water habitats within PAs will depend not only on management actions within individual PAs, but also, and strongly, on the use of water resources in the unprotected landscapes in the basins in which the PAs are found. Therefore, an integrated management planning of water resources, involving multiple sectors from agriculture to water supply to cities, would be required to achieve the persistence of water habitats and associated species, even if found within formally designated PAs, in the decades to come [48, 49]. Future work considering field water quality monitoring data would improve our understanding of the actual effectiveness of the protection of surface water, and of the ability of the protected water bodies to support the associated biodiversity in the long run [47]. The combination of recently available Sentinel 2A / 2B with Landsat imagery will allow near real-time monitoring of global surface water at higher spatial resolution (10-20m vs 30m currently). Work is also underway to more clearly delineate the nature of change: for example, identifying dams and water volume changes within PAs. Detailed statistics and trends at the individual PA level are available from the Digital Observatory for Protected Areas (DOPA) of the Joint Research Centre of the European Commission [42], accessible at http://dopa.jrc.ec.europa.eu, and the system will further report summaries at country and ecoregion level. In addition, tools currently being developed within DOPA will include the possibility for the user to select customized periods for surface water trends within the PAs.

## Supporting information

S1 Appendix Estimates of global surface water from previous studies. (DOCX)

S2 Appendix Details of the validation of GSWE, with reasoning for, and effect of, the 5% and 10% percentage thresholds applied in this analysis. (DOCX)

S3 Appendix Details of processing for the protected area geometries. (DOCX)

S4 Appendix Illustration of the location and impact of point-only PAs in the WDPA version used for this analysis. (DOCX)

S1 Table Country water coverage and water protection. Percentages of each country’s area which is covered by water (seasonal, permanent and all surface water) and the percentages of that water that is protected. PAs with point geometries only are assessed separately and their effect on the total protection is noted.
(PDF)

S2 Table Net change in area (1984–2015) of permanent and seasonal water inside and outside each country’s protected areas. (PDF)

S3 Table Overall net change trends (1984–2015) in water inside and outside each country’s protected areas. This table also highlights the cases where trends are altered by including buffered points.
(PDF)
