# Supplementary material for: Inland surface waters in protected areas globally: Current coverage and 30-year trends
Source: PLoS One. 2019 Jan 17;14(1):e0210496. doi: 10.1371/journal.pone.0210496 (PMC6336238; doi:10.1371/journal.pone.0210496)
Supplement: S1 Appendix — (DOCX) [file pone.0210496.s001.docx]

**S1 Appendix.**

**Estimates of global surface water from previous studies.**

Our analysis helps measure progress towards the CBD’s Aichi Target 11 and its quantitative target to conserve 17% of terrestrial and inland water areas in effective protected area systems by 2020. However, the dataset of global surface water bodies (e.g. lakes, rivers, ponds and streams) used for our analysis does not fully cover the CBD definition of “inland waters”, which also includes groundwater, springs, cave waters, floodplains, bogs, marshes, swamps and even the land that is “an integral part of an inland water ecosystem” (REF: [https://www.cbd.int/waters/inland-waters/default.shtml](https://webmail.ec.europa.eu/owa/redir.aspx?C=O7pSBhB4qLVQ-s35WxSPvjZKA5xR6Y0Z8403XUMgiRIaYe6CQjvWCA..&URL=https%3a%2f%2fwww.cbd.int%2fwaters%2finland-waters%2fdefault.shtml)). Hence the CBD definition of inland waters is difficult to map and measure, and, to the best of our knowledge, the CBD has never officially adopted a global map or estimate of inland water extent. The great inter-annual and intra-annual variation in global surface water and wetland areas further complicates the matter. As a result, previous estimates of the global extent of inland waters have varied greatly, for example from 530 to 970 million hectares (Revenga & Kura 2003). Based on those estimates, our analysis of permanent and seasonal surface water bodies covers approximately 22 - 68% of the global extent of inland and coastal wetland areas (estimated at 530-1,620 million ha; Table S1.1).

**Table 1.1.** Comparison of global estimates of inland surface water extent and protection.

| **Reference** | **Definition** | **Global extent** | **Global extent protected** |
| --- | --- | --- | --- |
| Revenga & Kura 2003 | Inland waters | 530-970 million ha | Not analysed |
| Juffe-Bignoli et al. 2014, using Lehner & Döll 2014 | Lakes, reservoirs, rivers and six major inland wetland types from the Global Lakes and Wetlands Database | 904 million ha | 187 million ha (20.7%) |
| Ramsar Convention on Wetlands 2018 | Inland and coastal wetland area | 1,210 million ha | Not analysed |
| Davidson & Finlayson 2018 | Inland and coastal wetland area | 1,520-1,620 million ha | Not analysed |
| This analysis, using Pekel et al. 2016 | Permanent and seasonal surface water from the Global Surface Water Explorer | 359 million ha:  278 m ha permanent  81 m ha seasonal | 59 million ha:  43 m ha permanent  16 m ha seasonal |

**References**

Davidson Nick C., Finlayson C. Max (2018) Extent, regional distribution and changes in area of different classes of wetland. *Marine and Freshwater Research* **69**, 1525-1533.

Juffe-Bignoli, D., Burgess, N.D., Bingham, H., Belle, E.M.S., de Lima, M.G., Deguignet, M., Bertzky, B., Milam, A.N., Martinez-Lopez, J., Lewis, E., Eassom, A., Wicander, S., Geldmann, J., van Soesbergen, A., Arnell, A.P., O’Connor, B., Park, S., Shi, Y.N., Danks, F.S., MacSharry, B., Kingston, N. (2014). Protected Planet Report 2014. UNEP-WCMC: Cambridge, UK.

Lehner, B. and Döll, P. (2004) ‘Development and validation of a global database of lakes, reservoirs and wetlands’, Journal of Hydrology 296: 1-22.

Ramsar Convention on Wetlands. (2018). Global Wetland Outlook: State of the World’s Wetlands and their Services to People. Gland, Switzerland: Ramsar Convention Secretariat.

Revenga, C. and Y. Kura. 2003. Status and Trends of Biodiversity of Inland Water Ecosystems. Secretariat of the Convention on Biological Diversity, Montreal, Technical Series no. 11.
