# Supplementary material for: Inland surface waters in protected areas globally: Current coverage and 30-year trends
Source: PLoS One. 2019 Jan 17;14(1):e0210496. doi: 10.1371/journal.pone.0210496 (PMC6336238; doi:10.1371/journal.pone.0210496)
Supplement: S2 Appendix — (DOCX) [file pone.0210496.s002.docx]

**S2 Appendix.**

**Details of the validation of GSWE, with reasoning for, and effect of, the 5% and 10% percentage thresholds applied in this analysis.**

The water classifications of the Global Surface Water Explorer (permanent, seasonal, and transition between the two) are based on a time series analysis which takes into account the consistency of observations for each pixel across time. For each monthly time step, a pixel can fall into one of three classes: ‘water’, ‘non-water’ or ‘non-valid observation’. The third label can stem from a number of causes: for example, there may be a gap in the Landsat archive for that location at that time, the area may have been obscured by clouds or the acquired signal may have been rendered invalid by instrument error – for example, the ‘striping’ on Landsat 7 imagery caused by the failure of the Scan Line Corrector. To some extent there is a spatial pattern to these non-valid observations: the Landsat archive is most complete in space and time over the conterminous United States, while over certain forested areas of the world it is notoriously difficult to achieve a cloud-free observation. Once these problematic areas are discounted, we are left with a series of monthly binary maps, which are used to define the nature of any visible surface water based on its recurrence and consistency over the year, between years and between months. The full methodology is described in the supplementary materials of Pekel et al. (2016).

The binary water maps were validated using photointerpretation over a set of more than 40 thousand reference points, whose global distribution is shown in Figure 2.1.
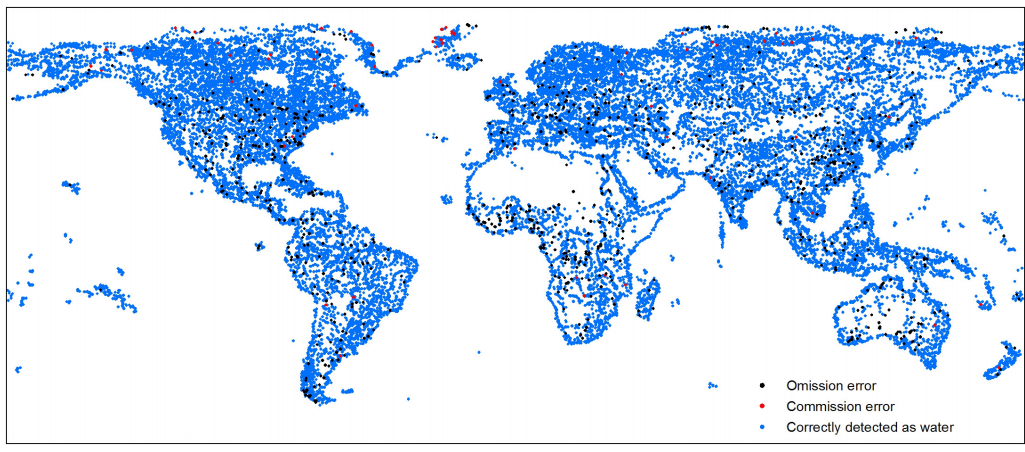


**Figure 2.1. Locations of the sample points in the validation database (reproduced with permission from Pekel et al., 2016).**

This validation process confirmed that the product contains less than 1% of false water detections, and misses less than 5% of water. This 5% level is therefore a well-justified figure to use when assessing the patterns of permanent water. It can be assumed that up to 5% of a country’s water which is detectable by the classifier (i.e., which is sufficiently large to dominate a 30m Landsat pixel) might be missed at any one time step. This affects the detection of permanent water because it will impact on the presence of a consistent series throughout any one year. For this reason, the authors of the Global Surface Water Explorer chose a 5% level of detections when generating the trends reported in Pekel et al (2016). An illustrative figure has been reproduced from that paper, with the authors’ permission, and is shown in Figure 2.2. It can be seen that when generating trend lines, only those years with an unobserved component less than 5% were included.


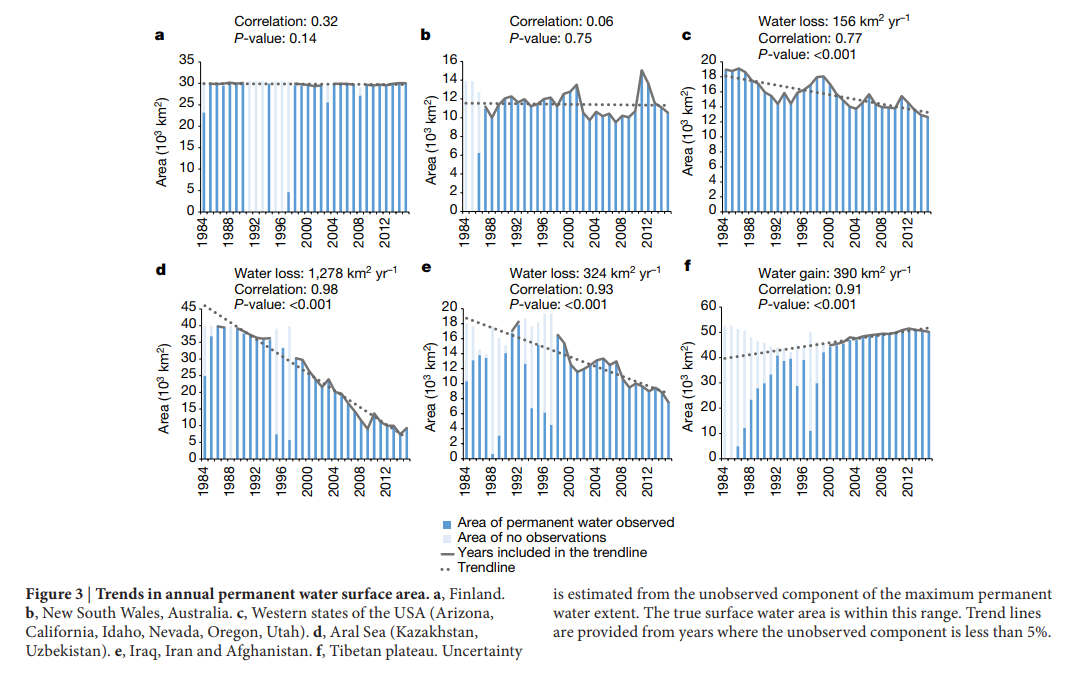


**Figure 2.2. Trends in annual permanent water surface area, from (a) Finland, (b) New South Wales, Australia, (c) Western states of the USA (Arizona, California, Idaho, Nevada, Oregon, Utah), (d) Aral Sea (Kazakhstan, Uzbekistan), (e) Iraq, Iran and Afghanistan, (f), Tibetan plateau. This figure is reproduced with permission from Pekel et al. (2016) and shows how the authors estimate uncertainty from the unobserved component of the maximum permanent water extent. Trend lines only use those years where the unobserved component is less than 5%.**

For seasonal water, the challenge of accurate delineation is greater, since there are fewer opportunities to observe each water body, and non-valid observations will have a higher impact. The validation exercise identified higher errors of omission for seasonal than permanent water, with up to 25% of seasonal water being missed at any single time step. However, it would be over-simplistic to simply adopt this 25% threshold for seasonal water, since, to quote Pekel et al. ‘over the 32 years, multiple opportunities for observation arise. Thus sites where seasonal water can occur may be missed at one date, but may be correctly mapped at another. Overall, less than 1% of the points in the validation database where water was present remained entirely unmapped over time’. In other words, while seasonal water is indeed easier to miss at any one point in time, as a category it is more robust to single omissions than permanent water, which could be reclassified as ‘seasonal’ because of very small gaps in the time series caused by the limited (albeit high) detection accuracy of 95%.

Taking all this evidence into consideration, we decided on a higher threshold for seasonal than permanent water, at a compromise level of 10%. The next factor we needed to consider was whether the threshold we applied to define significant net gain or loss should vary across space: for example, whether the leeway applied should vary according to climate zone or latitude. The map of errors in Figure 2.2 shows that the errors are not significantly clustered in space, and there is not sufficient evidence for a varying threshold based on latitude. Figure 2.3 shows the spatial distribution of valid observations over the 32 years (which have the potential to bias the detection of both seasonal and permanent water); there is no particular gradient or spatial correlate which could be used as a reliable weighting for the threshold.
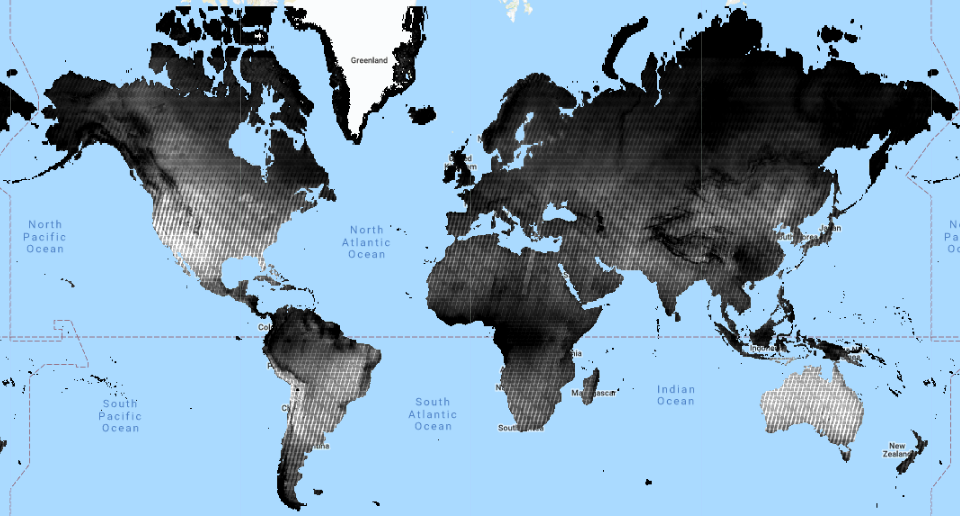


**Figure 2.3. Spatial distribution of valid observations**

In order to assess whether these thresholds were masking any important patterns in relative net gain and loss for countries, we have applied a range of alternative thresholds from those chosen down to zero, and these are illustrated in Figures 2.4 to 2.7. In all four of these charts, it can be seen that the underlying ratios of change persist whichever threshold is applied. In the case of Figures 2.6 and 2.7, it can be seen that the disparity between protected and unprotected areas in a number of countries remains consistent regardless of the threshold. Generally, in a majority of countries, seasonal surface water area increased both inside and outside protected areas, while permanent water shows a very different trend: in a large number of cases it is increasing outside protected areas and decreasing inside, possibly because of dams and extractive practices. (It should be noted that in all of these charts, the protected and unprotected bars do not achieve the country total because some countries do not have any surface water in one or both of those zones.)


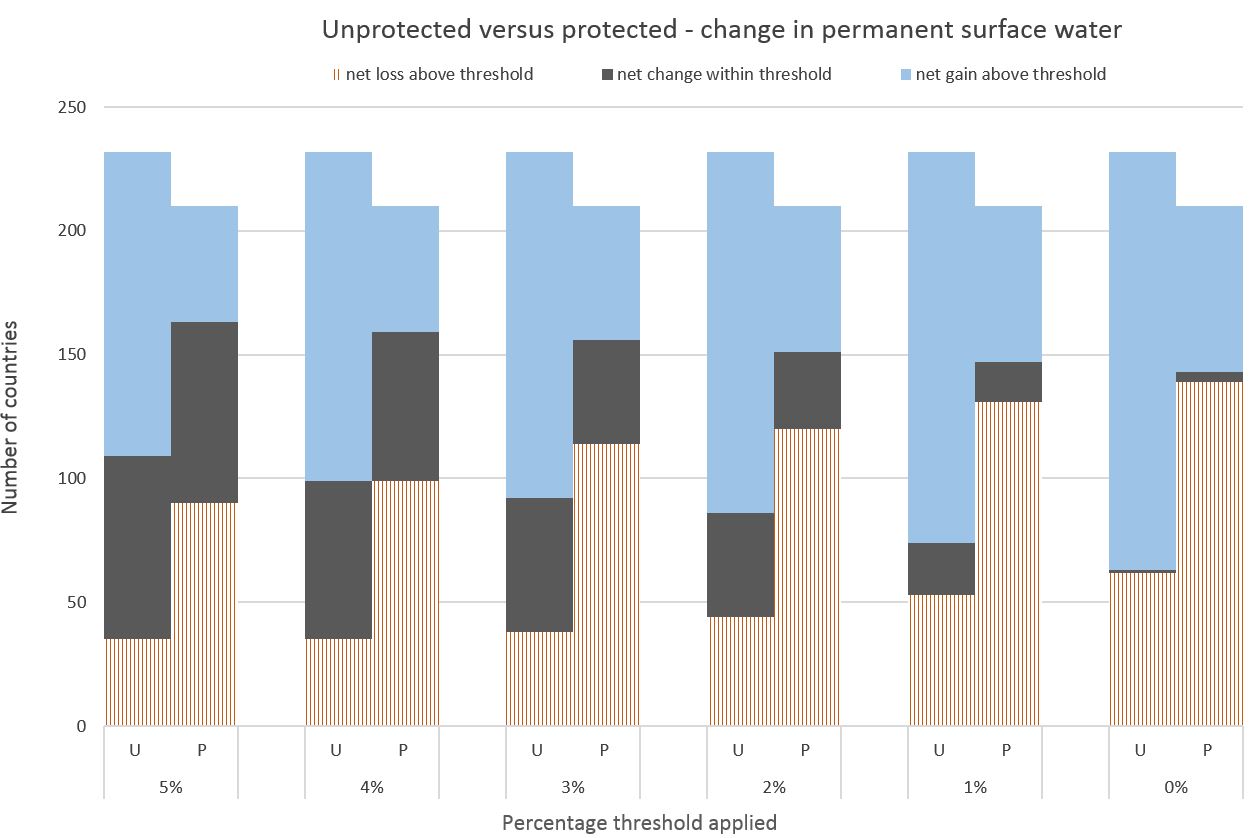


**Figure 2.4. Net change in permanent surface water, by country, in protected and unprotected areas, for different thresholds from 0 to 5%.**

**
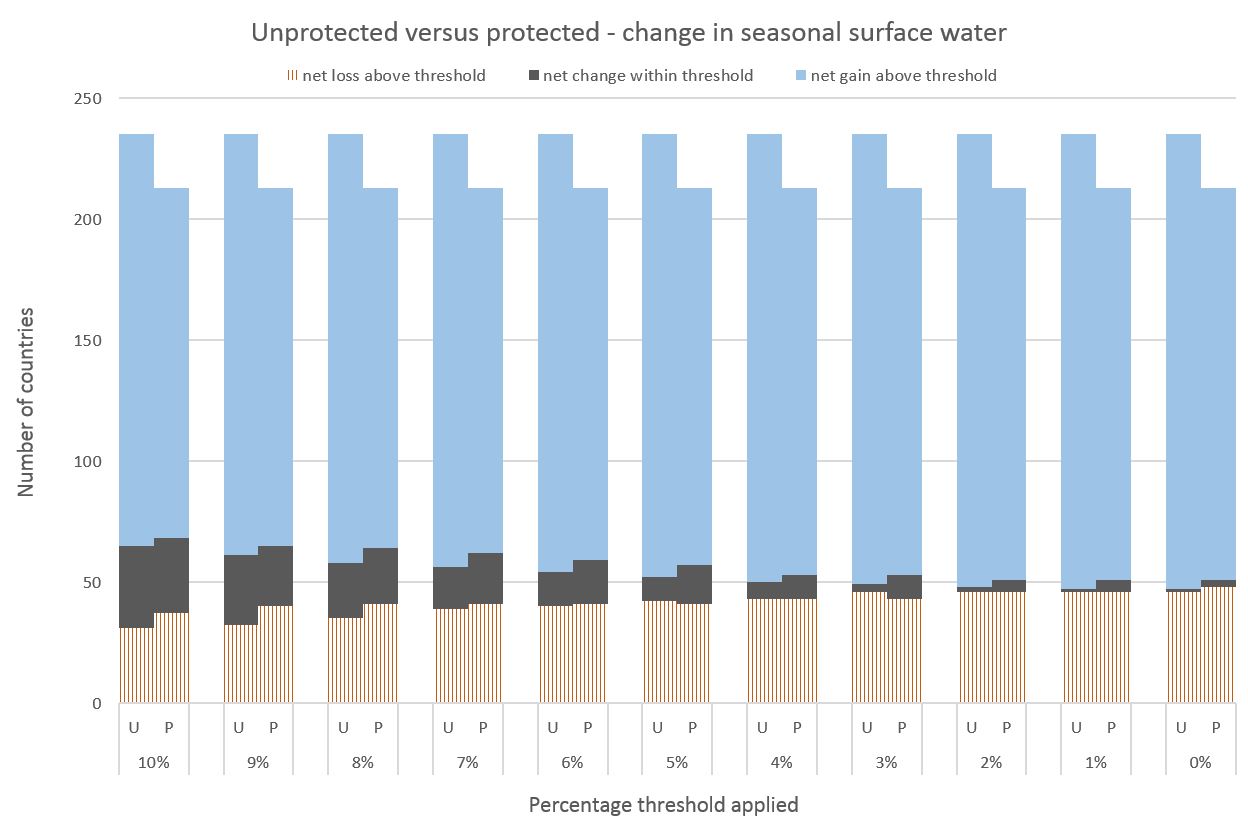
**

**Figure 2.5. Net change in seasonal surface water, by country, in protected and unprotected areas, for different thresholds from 0 to 10%.**

**
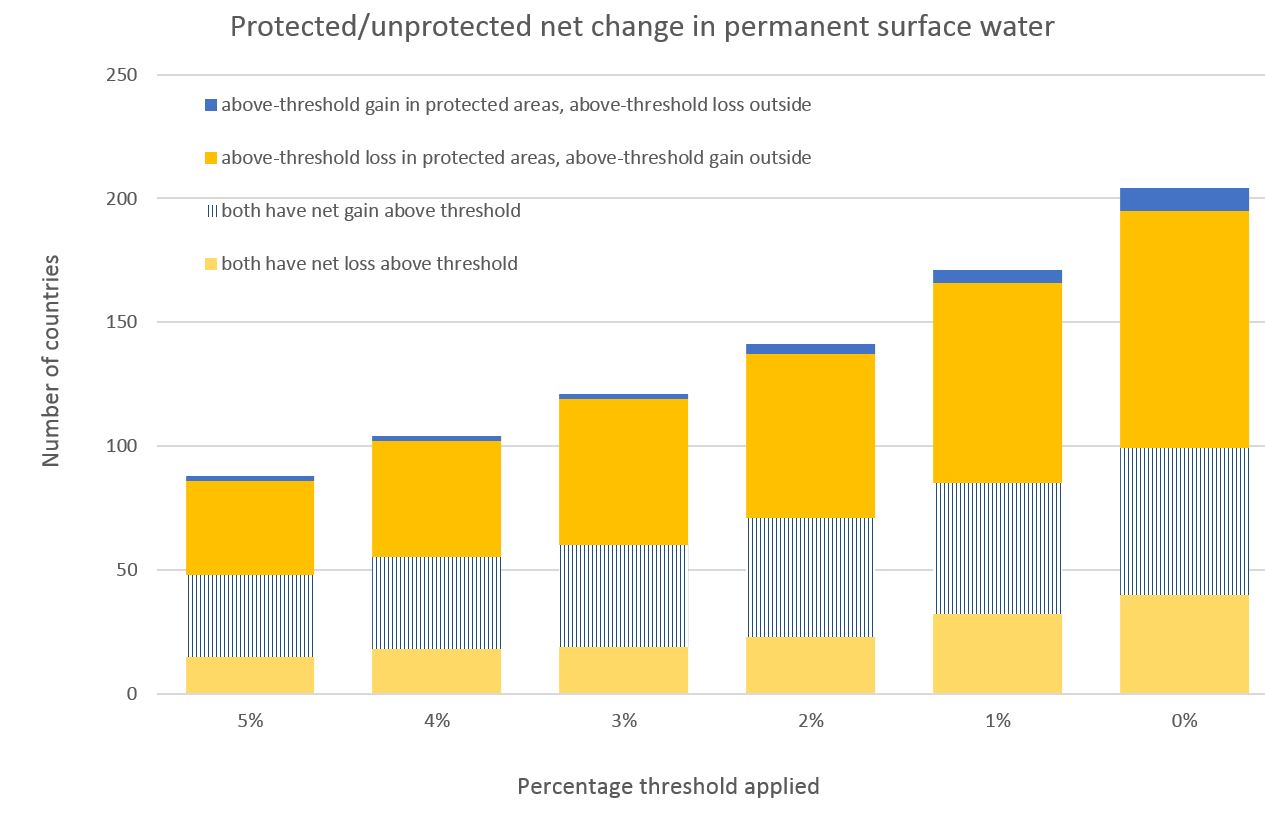
**

**Figure 2.6. Relationship between net change in permanent surface water in protected and unprotected areas, by country, for different thresholds from 0 to 5%.**

**
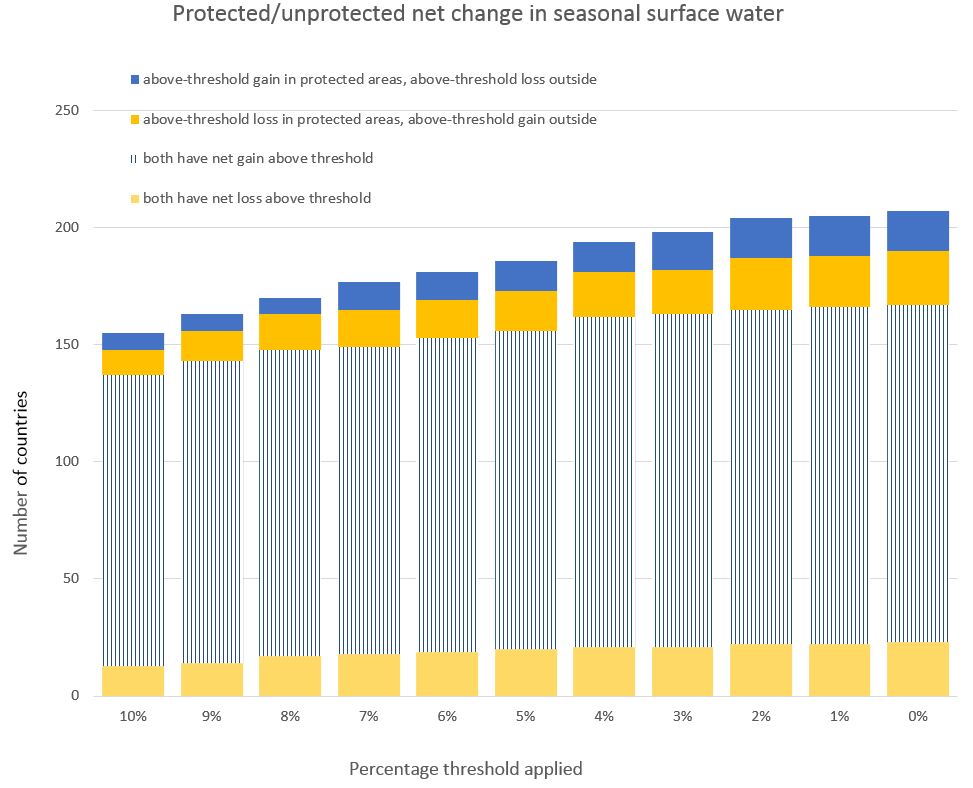
**

**Figure 2.7. Relationship between net change in seasonal surface water in protected and unprotected areas, by country, for different thresholds from 0 to 10%.**

Finally, a set of maps were produced to illustrate where exactly threshold changes would have an effect. These are shown in Figures 2.8 and 2.9.


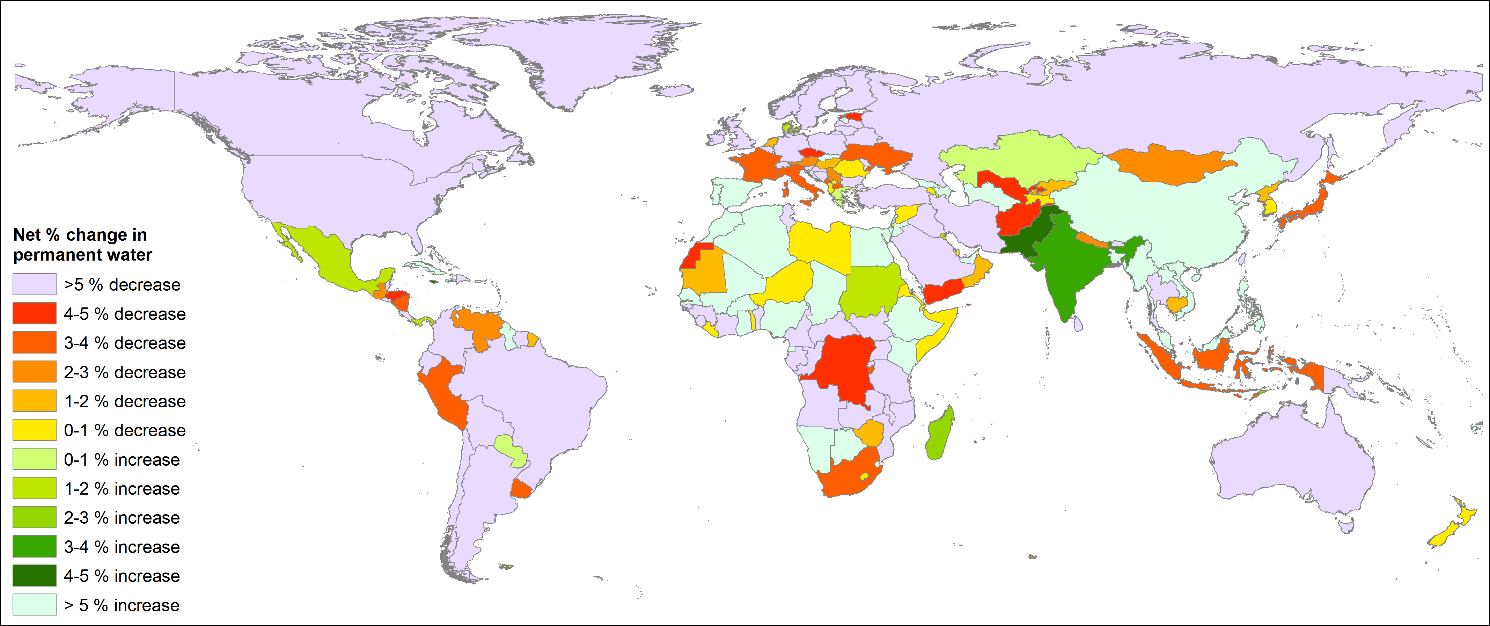


**Figure 2.8. Countries where net gain/loss for permanent surface water falls within the threshold of 5%.**

**
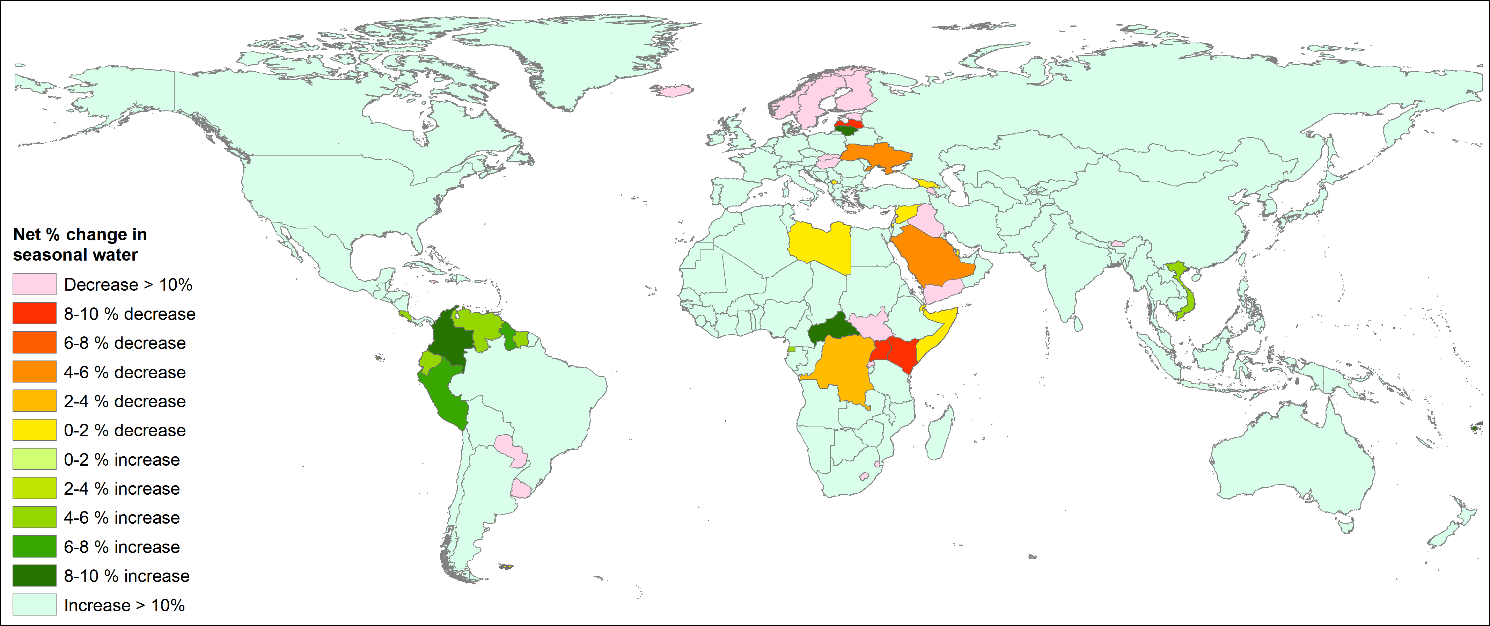
**

**Figure 2.9. Countries where net gain/loss for seasonal surface water falls within the threshold of 10%.**
