# Supplementary material for: Inland surface waters in protected areas globally: Current coverage and 30-year trends
Source: PLoS One. 2019 Jan 17;14(1):e0210496. doi: 10.1371/journal.pone.0210496 (PMC6336238; doi:10.1371/journal.pone.0210496)
Supplement: S3 Appendix — (DOCX) [file pone.0210496.s003.docx]

**S3 Appendix.**

**Details of processing for the protected area geometries**

The following steps were followed to process the PA geometries in the World Database on Protected Areas (WDPA) and to combine them with the Global Surface Water Explorer (Pekel *et al.* 2016):

1. The April 2016 version of the WDPA (IUCN and UNEP-WCMC 2016) was downloaded from <https://www.protectedplanet.net/> in the form of a feature geodatabase.

2. Protected areas with point-only geometries and a reported area > 0 were given a circular geodesic point buffer with the reported area.

3. Polygon self-intersections were corrected in PostGIS, and point and line geometries resulting from the correction were discarded.

4. Polygons which had been digitised with only two coordinates (e.g. some sunken vessels protected for their marine habitat value) were converted to lines and buffered by a very small distance to create valid polygons.

5. Features at the dateline whose geometries fell outside legal coordinates were split, and the remaining part of the geometry translated to the correct side of the dateline.

6. As recommended by UNEP-WCMC (UNEP-WCMC and IUCN 2016), the data was filtered to remove all features with a status of "not reported" or "proposed", and all features designated as UNESCO Man and the Biosphere (MAB) Reserves.

7. The polygon PAs were converted to a binary raster with a cell size of 3 arc seconds (representing a resolution of c. 31m at the equator). The resulting raster was used as an asset in Google Earth Engine to mask the transition layer of the Global Surface Water Explorer and identify protected and unprotected water of different types.

8. The geodesic buffers for point-only PAs were converted to a binary raster of the same resolution, and this was used as a supplementary mask to estimate the added protection offered by these PAs.
