# Supplementary material for: Inland surface waters in protected areas globally: Current coverage and 30-year trends
Source: PLoS One. 2019 Jan 17;14(1):e0210496. doi: 10.1371/journal.pone.0210496 (PMC6336238; doi:10.1371/journal.pone.0210496)
Supplement: S4 Appendix — (DOCX) [file pone.0210496.s004.docx]

**S4 Appendix.**

**Illustration of the location and impact of point-only PAs in the WDPA version used for this analysis.**

While most PAs in the WDPA have delineated polygon boundaries, a subset consists of only a geographic point (assusmed usually to be a centroid) and a reported area. In the absence of other data, the recommendation of the data publishers is to buffer these centroids with a geodesic circular buffer whose area corresponds to the reported area [1]. This is the method used in the Protected Planet report [2] and in many other publications [e.g. 3,4] to avoid undue bias in countries where fully-mapped boundaries are currently unavailable.

The distribution of these point-only PAs is geographically biased and dependent on geopolitical factors. Figure 4.1 shows the distribution and size of these buffer polygons within the April 2016 version of the WDPA used for this analysis.


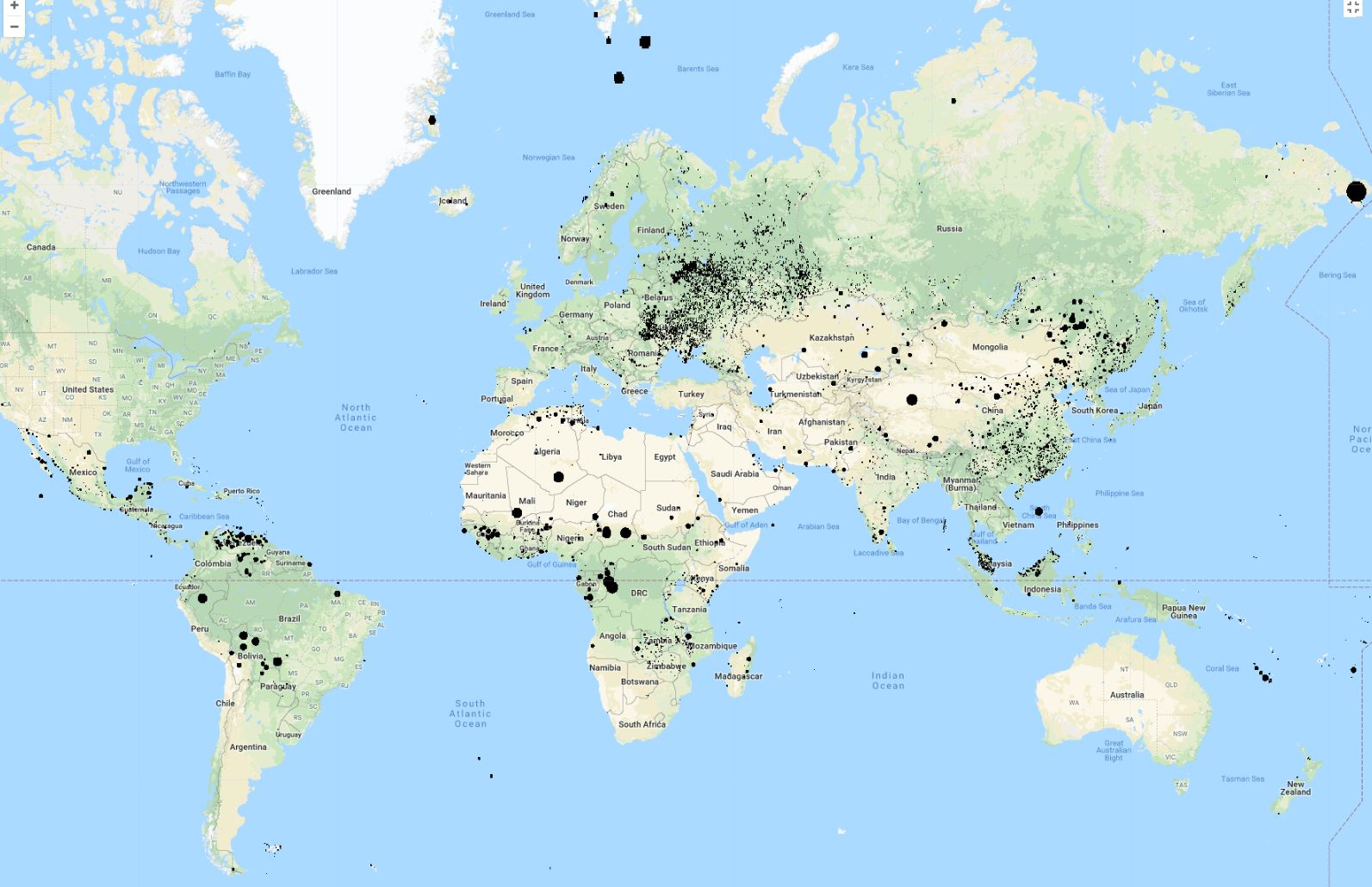


**Figure 4.1. Location of point buffers in the World Database on Protected Areas of April 2016.**

For some countries, the major part of their protected area extent is recorded in this form, despite constant efforts from the database managers to improve this situation. Figure 4.2 shows the proportion of protection in point-only form for the April 2016 version of the WDPA.


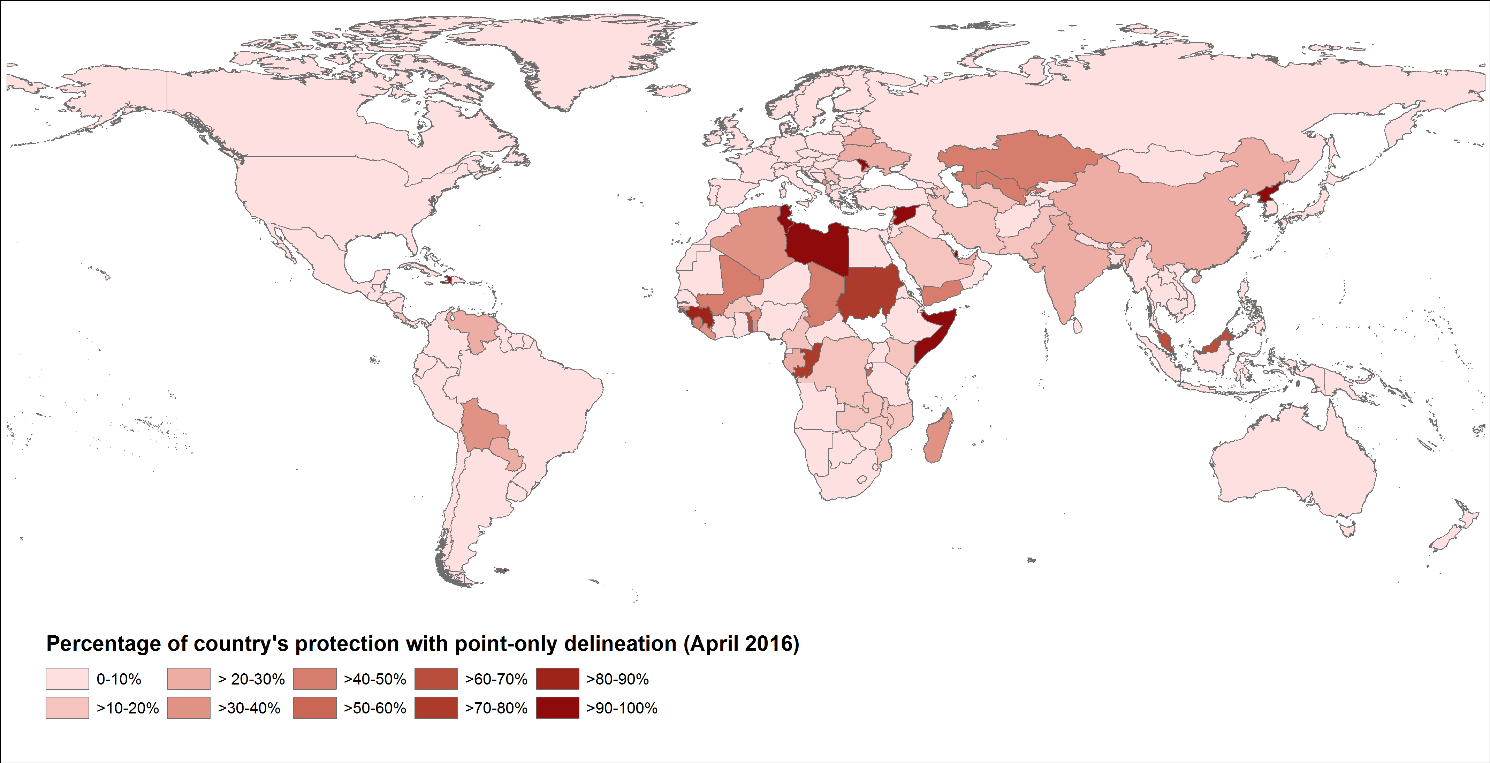


**Figure 4.2 Point-only protection coverage in the World Database on Protected Areas of April 2016.**

It can be seen that this bias in the data will affect assessments of water protection. Figure 4.3 compares the proportion of permanent water protected when point buffers are not considered, compared to the case when these geometries are included.


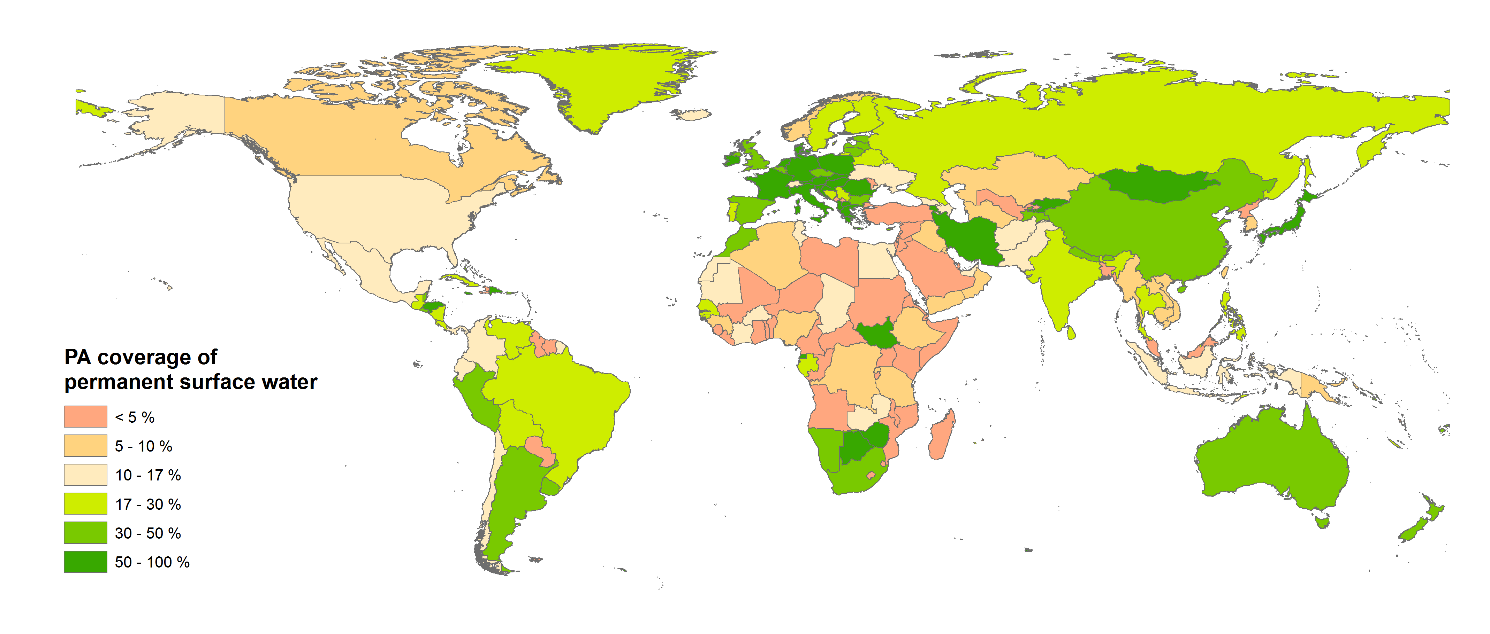


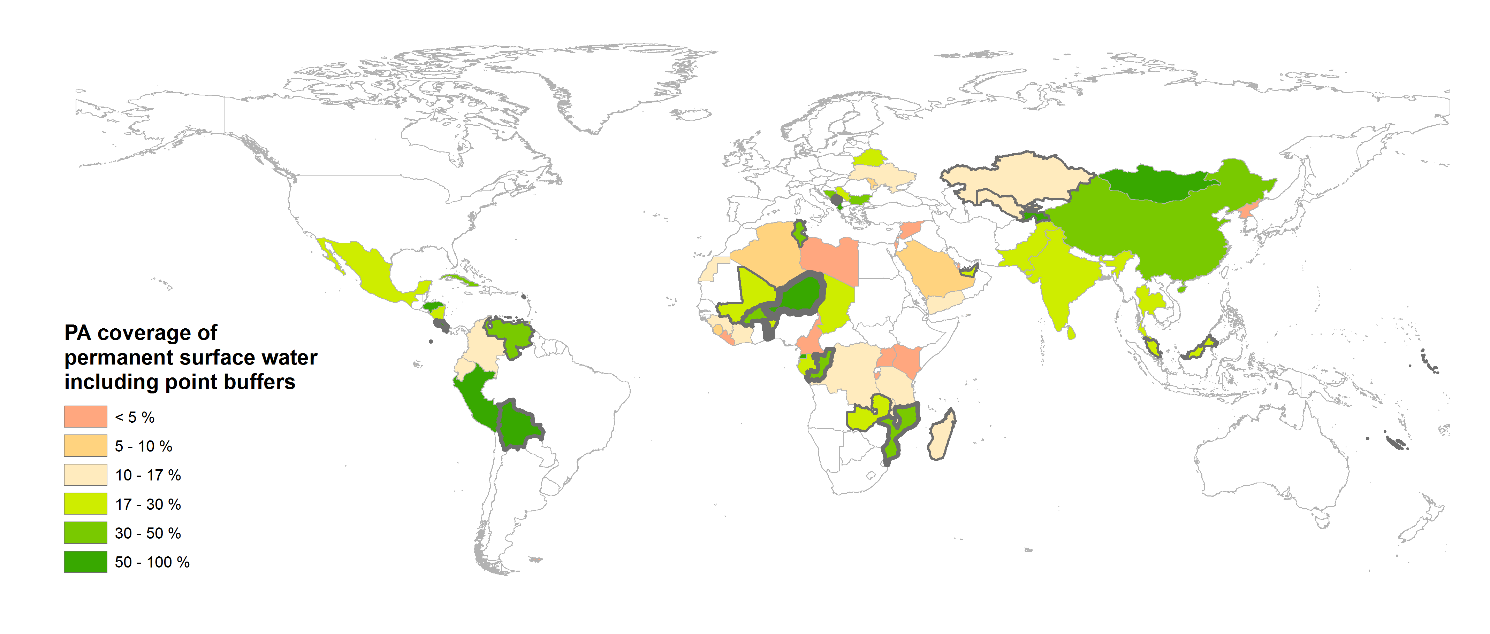


**Figure 4.3 Effect of including point buffers in estimation of permanent surface water protection.** In the lower figure, only those countries where point buffers induce a change above 0.5 % in protection percentage are coloured, and the width of the country outline denotes the level of the change.

Figure 4.4 illustrates the same comparison for seasonal surface water.


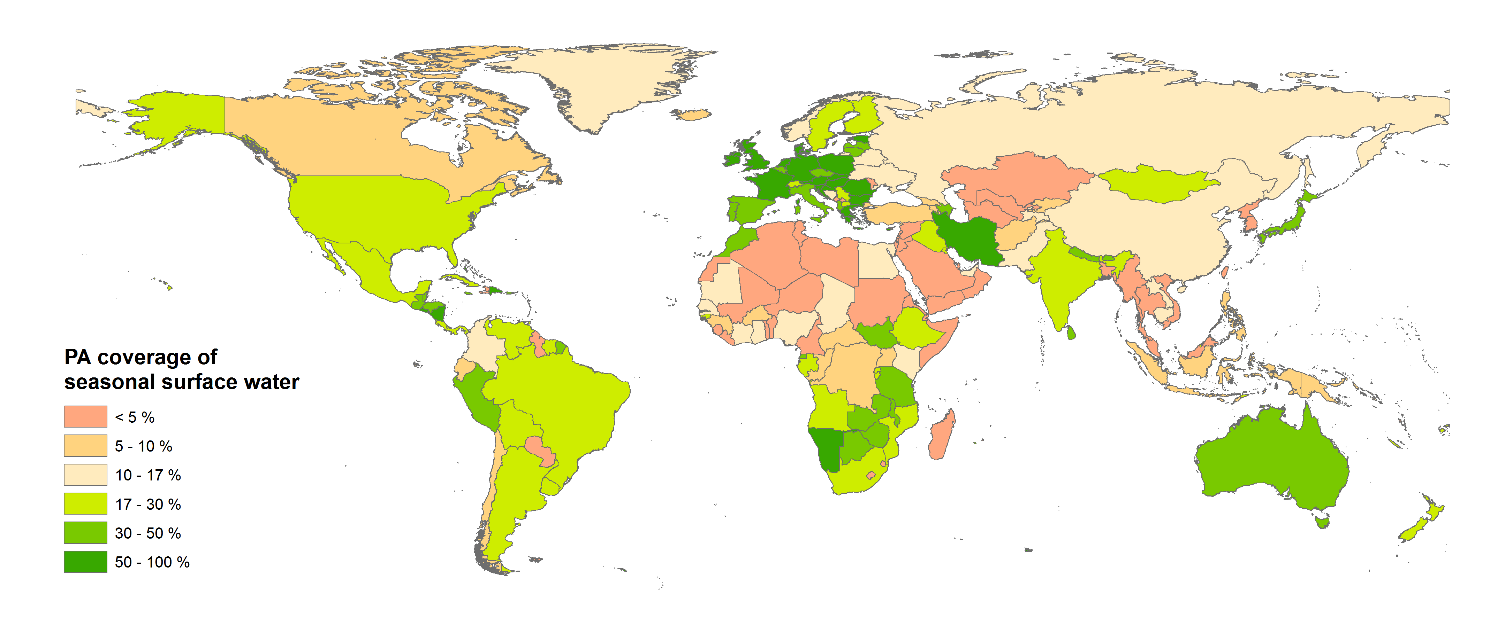

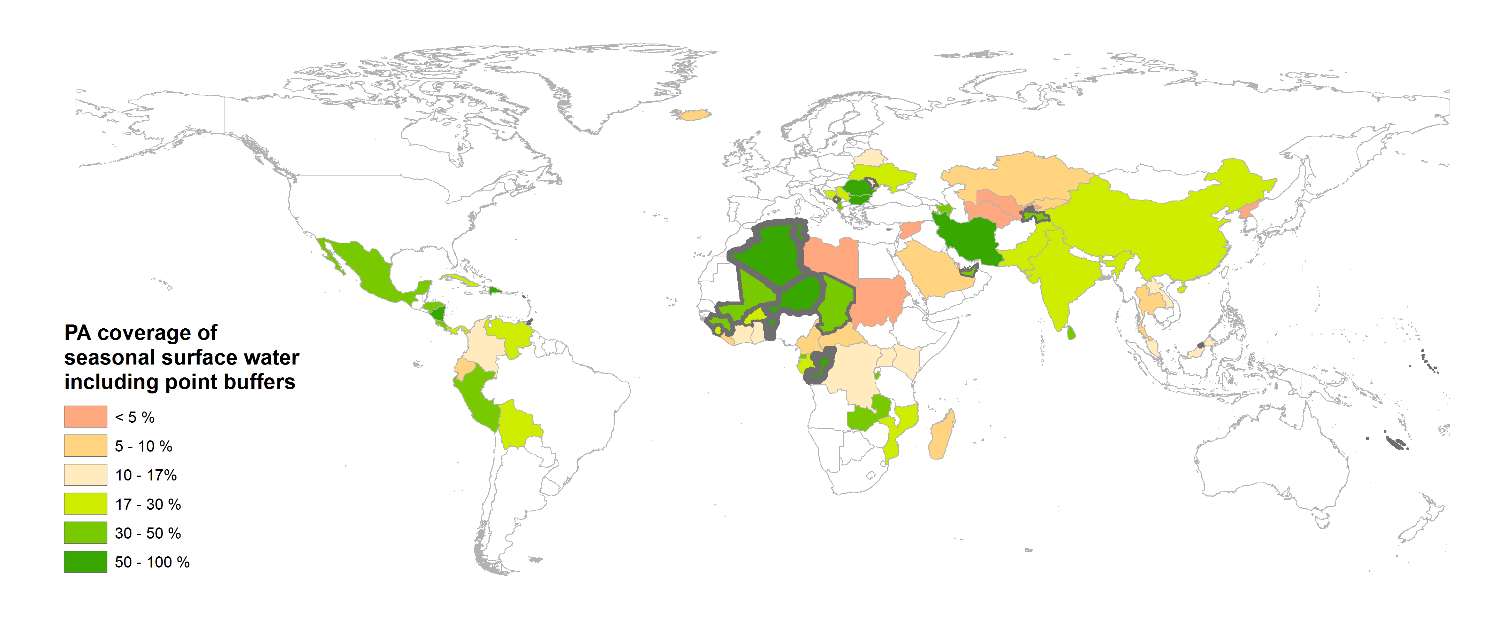


**Figure 4.4 Effect of including point buffers in estimation of seasonal surface water protection.** In the lower figure, only those countries where point buffers induces a change in protection percentage over 0.5 % are coloured, and the width of the country outline denotes the level of the change.

While these point buffers do not accurately delineate protected area boundaries, they may at least capture some of the habitats at a site, and there is an argument that they should be included rather than excluded from analysis.

The contribution of these point buffers to apparent water protection is substantial in some countries. Tables S3.1 and S3.2 list countries in order of the extra water protection apparently afforded by these geometries, and show the cases where overall trends inside and outside protected areas (applying the same 5% and 10% thresholds as in the main text) are changed by the inclusion of the point buffers. The results are substantial for some countries, but it is important to remember that the definitions of ‘inside’ and ‘outside’ PAs are strongly limited by the simple geometric representations [5]. In many cases, water protection may be underestimated by the circular buffers. An example of this can be clearly seen in Figure 4.5 – an example along the Danube where the circular buffers, because of multiple designations at the same site, often refer to the same protected zones as polygons which also exist in the database. In this case it can be seen that on their own, the point buffers would capture little of the river’s structure and connectivity. However when the polygon PAs are added to the map, it can be seen that the circular areas should in reality be more tightly related to the river’s configuration. In this particular case, little is added by including the point buffers, but if no polygon PA boundaries were available, their inclusion would at least mitigate the underestimation of protected water features.


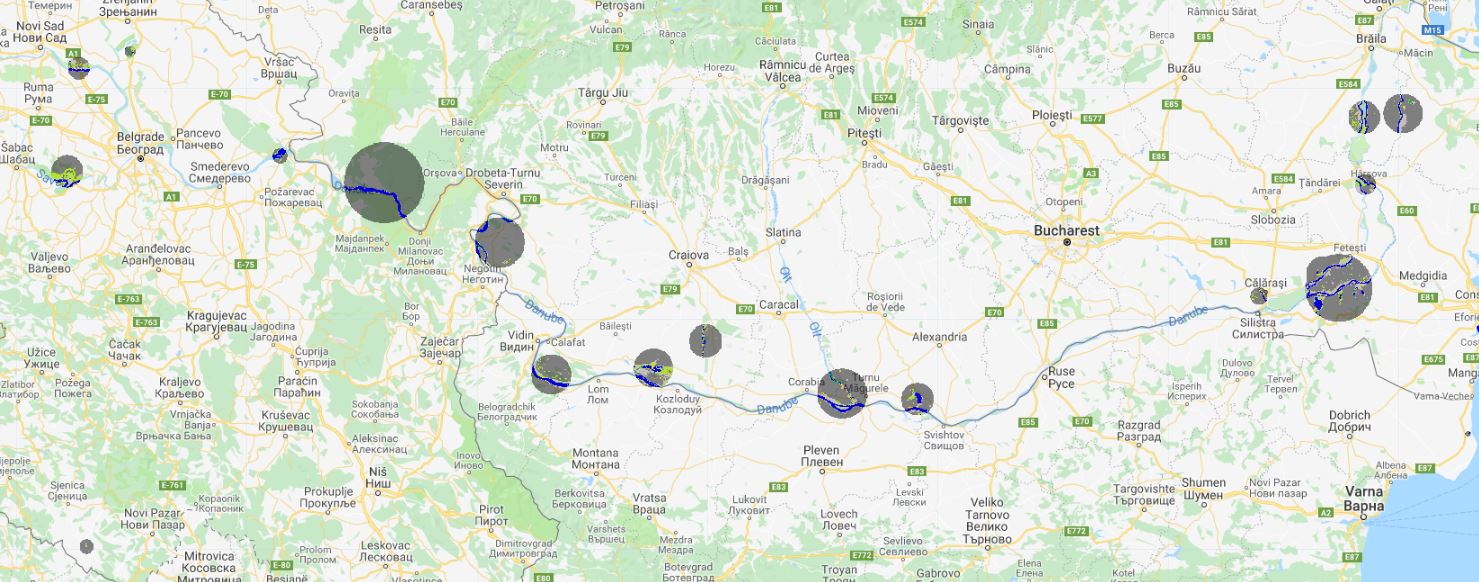

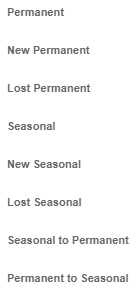

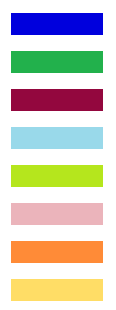

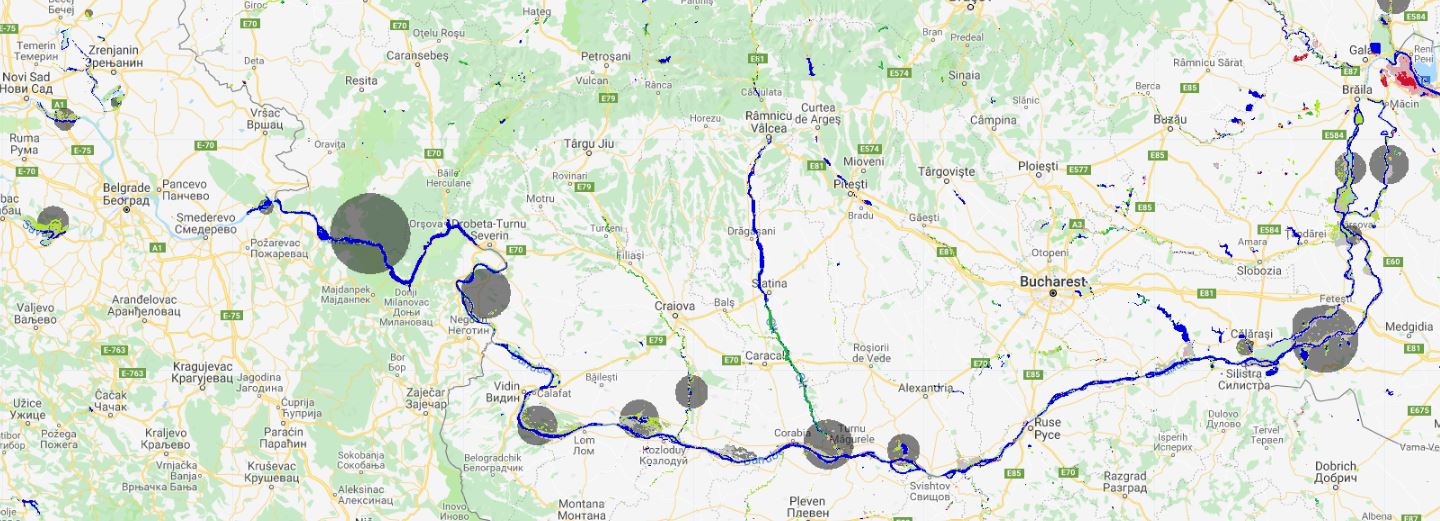


**Figure 4.5 Comparison of protected water along the Danube considering point buffers only (top panel) and including all other PA polygons (bottom panel). The key shows water tranistion classes which can be more fully explored at https://global-surface-water.appspot.com/**

**References**

1. UNEP-WCMC. World Database on Protected Areas User Manual 1.5. 2017. UNEP-WCMC: Cambridge, UK.

UNEP-WCMC and IUCN. Protected Planet Report 2016. UNEP-WCMC and IUCN: Cambridge UK and Gland, Switzerland.

1. Saura S, Bastin L, Battistella L, Mandrici A, Dubois G. Protected areas in the world’s ecoregions: how well connected are they? Ecol Indic. 2017; 76: 144-158.
2. Saura S, Bertzky B, Bastin L, Battistella L, Mandrici A, Dubois G. Protected area connectivity: shortfalls in global targets and country-level priorities. Biol Conserv. 2018; 219: 53-67
3. Abell R, Allan JD, Lehner B. Unlocking the potential of protected areas for freshwaters. Biol Conserv. 2007; 134: 48-63.
