# Supplementary material for: Inland surface waters in protected areas globally: Current coverage and 30-year trends
Source: PLoS One. 2019 Jan 17;14(1):e0210496. doi: 10.1371/journal.pone.0210496 (PMC6336238; doi:10.1371/journal.pone.0210496)
Supplement: S1 Table — Percentages of each country’s area which is covered by water (seasonal, permanent and all surface water) and the percentages of that water that is protected. PAs with point geometries only are assessed separately and their effect on the total protection is noted. (PDF) [file pone.0210496.s005.pdf]

IW = *inland permanent and seasonal water combined;* IPW = *inland permanent water;* ISW = *inland seasonal water*										
**Country name**	**% of country's land area that is IW**	**% of country's land area that is IPW**	**% of country's land area that is ISW**	**% of IPW that is protected**	**% of ISW that is protected**	**% of IW that is protected**	**% of IPW protected (including point buffers)**	**% of ISW protected (including point buffers)**	**% of IW protected (including point buffers)**	**Additional estimated percentage from buffers**
**Afghanistan**	0.33	0.09	0.24	11.23	5.11	6.78	11.23	5.11	6.78	0.00
**Akrotiri and Dhekelia**	5.48	0.93	4.55	0.00	0.00	0.00	0.00	0.00	0.00	0.00
**Aland**	10.11	8.84	1.28	3.58	3.28	3.54	3.58	3.28	3.54	0.00
**Albania**	2.39	1.99	0.41	76.20	31.23	68.56	77.95	32.35	70.21	1.64
**Algeria**	0.13	0.02	0.11	5.63	3.40	3.82	6.97	65.50	54.34	50.51
**American Samoa**	3.27	1.69	1.58	20.70	13.55	17.24	20.70	13.55	17.24	0.00
**Andorra**	0.16	0.14	0.02	11.29	10.00	11.11	11.29	10.00	11.11	0.00
**Angola**	0.28	0.09	0.19	1.04	27.26	18.61	1.04	27.27	18.62	0.01
**Anguilla**	7.39	5.58	1.81	0.00	0.00	0.00	1.28	2.63	1.62	1.62
**Antigua and Barbuda**	4.92	3.44	1.48	77.11	51.85	69.51	77.11	51.85	69.51	0.00
**Argentina**	2.14	1.09	1.04	32.31	20.77	26.68	32.68	20.79	26.88	0.20
**Armenia**	4.63	4.50	0.13	94.56	8.79	92.07	94.56	8.79	92.07	0.00
**Aruba**	2.44	1.50	0.95	0.00	0.00	0.00	0.00	0.00	0.00	0.00
**Australia**	0.65	0.14	0.51	37.12	35.59	35.92	37.12	35.59	35.92	0.00
**Austria**	0.80	0.70	0.10	62.68	63.27	62.75	62.68	63.27	62.76	0.00
**Azerbaijan**	2.37	1.22	1.15	15.77	34.32	24.75	16.14	36.20	25.85	1.10
**Bahamas**	31.33	14.45	16.88	43.37	50.85	47.40	43.41	50.87	47.43	0.03
**Bahrain**	2.46	1.62	0.84	0.00	0.00	0.00	0.34	0.33	0.34	0.34
**Bangladesh**	14.50	2.55	11.95	3.23	0.75	1.19	3.63	1.16	1.60	0.41
**Barbados**	0.55	0.28	0.27	0.81	0.85	0.83	0.81	0.85	0.83	0.00
**Belarus**	1.17	0.86	0.31	22.98	11.16	19.83	24.65	15.16	22.11	2.29
**Belgium**	0.62	0.50	0.12	35.59	36.90	35.84	35.59	36.90	35.84	0.00
**Belize**	2.60	1.72	0.88	30.39	33.94	31.58	30.39	33.94	31.58	0.00
**Benin**	0.40	0.25	0.15	0.06	4.45	1.68	29.60	53.10	38.30	36.61
**Bermuda**	17.83	16.88	0.95	4.08	6.15	4.19	4.08	6.15	4.19	0.00
**Bhutan**	0.35	0.25	0.10	44.47	45.51	44.76	44.47	45.51	44.76	0.00
**Bolivia**	1.72	0.99	0.73	22.05	18.27	20.45	53.88	27.73	42.82	22.37
**Bonaire Saint Eustatius and Saba**	14.12	12.13	1.99	16.27	22.57	17.16	19.26	28.90	20.62	3.46
**Bosnia and Herzegovina**	0.60	0.42	0.18	27.38	16.83	24.19	31.27	20.30	27.95	3.77
**Botswana**	0.59	0.07	0.52	72.63	41.01	44.76	72.63	41.01	44.76	0.00
**Brazil**	1.86	1.34	0.52	17.52	26.67	20.08	17.67	26.92	20.26	0.18
**British Indian Ocean Territory**	15.17	9.50	5.67	100.00	100.00	100.00	100.00	100.00	100.00	0.00
**British Virgin Islands**	12.03	9.26	2.77	13.51	6.14	11.81	15.09	11.23	14.20	2.39
**Brunei**	1.03	0.71	0.32	20.05	18.66	19.62	23.85	38.87	28.52	8.90
**Bulgaria**	0.82	0.66	0.16	47.89	52.05	48.68	48.97	52.66	49.67	0.99
**Burkina Faso**	0.42	0.13	0.29	10.24	9.13	9.47	40.35	20.28	26.54	17.06
**Burundi**	7.40	7.35	0.06	0.22	24.38	0.40	1.73	31.06	1.95	1.55
**Cambodia**	5.31	1.90	3.41	5.41	13.01	10.29	5.41	13.01	10.29	0.00
**Cameroon**	0.75	0.44	0.31	2.56	3.91	3.11	4.33	8.55	6.07	2.96
**Canada**	11.11	10.30	0.81	6.98	9.80	7.19	6.98	9.81	7.19	0.00
**Cape Verde**	1.41	1.09	0.32	0.04	0.53	0.15	0.04	0.53	0.15	0.00
**Cayman Islands**	6.84	4.76	2.08	35.67	17.09	30.03	35.67	17.09	30.03	0.00
**Central African Republic**	0.17	0.10	0.07	1.63	6.29	3.67	1.71	7.40	4.19	0.53
**Chad**	0.33	0.11	0.22	13.10	12.39	12.63	17.63	38.11	31.36	18.73
**Chile**	1.95	1.74	0.21	15.98	8.81	15.21	16.02	8.81	15.25	0.04
**China**	1.89	1.23	0.65	32.98	14.17	26.47	37.02	18.24	30.52	4.05
**Colombia**	1.16	0.70	0.46	12.35	10.95	11.80	14.26	12.59	13.60	1.81
**Comoros**	1.61	1.12	0.49	0.00	0.00	0.00	0.05	0.12	0.07	0.07
**Cook Islands**	1.69	0.49	1.20	4.22	1.06	1.98	4.22	1.06	1.98	0.00
**Costa Rica**	0.66	0.46	0.20	21.64	21.25	21.52	57.91	30.48	49.48	27.95
**Cote dIvoire**	0.70	0.53	0.16	10.04	12.66	10.66	12.03	15.29	12.80	2.14

IW = *inland permanent and seasonal water combined*; IPW = *inland permanent water*; ISW = *inland seasonal water*										
**Country name**	**% of country's land area that is IW**	**% of country's land area that is IPW**	**% of country's land area that is ISW**	**% of IPW that is protected**	**% of ISW that is protected**	**% of IW that is protected**	**% of IPW protected (including point buffers)**	**% of ISW protected (including point buffers)**	**% of IW protected (including point buffers)**	**Additional estimated percentage from buffers**
**Croatia**	1.00	0.68	0.33	72.45	90.87	78.43	72.45	90.87	78.43	0.00
**Cuba**	2.92	1.69	1.22	29.87	26.59	28.49	31.61	27.22	29.77	1.27
**Curacao**	3.19	2.16	1.02	27.91	21.51	25.85	27.91	21.51	25.85	0.00
**Cyprus**	0.43	0.25	0.18	46.07	73.36	57.68	46.07	76.21	58.89	1.21
**Czech Republic**	0.80	0.64	0.16	37.35	32.11	36.31	37.35	32.11	36.31	0.00
**Democratic Republic of the Congo**	1.72	1.53	0.19	6.75	5.99	6.66	16.19	14.96	16.06	9.39
**Denmark**	1.65	1.38	0.28	83.10	87.19	83.78	83.10	87.19	83.78	0.00
**Djibouti**	1.12	0.63	0.48	0.00	0.00	0.00	0.03	0.25	0.13	0.13
**Dominica**	0.38	0.24	0.14	3.26	2.91	3.14	3.26	2.91	3.14	0.00
**Dominican Republic**	1.15	0.93	0.22	69.27	51.64	65.96	69.35	52.53	66.19	0.24
**East Timor**	0.38	0.12	0.27	22.32	25.33	24.40	22.32	25.33	24.40	0.00
**Ecuador**	1.47	1.00	0.47	11.80	7.22	10.34	13.34	8.61	11.84	1.49
**Egypt**	0.95	0.67	0.28	12.27	14.80	13.01	12.42	15.11	13.20	0.19
**El Salvador**	1.80	1.15	0.65	41.79	69.37	51.72	42.02	69.68	51.97	0.25
**Equatorial Guinea**	0.50	0.41	0.09	76.85	45.07	71.33	77.54	48.26	72.45	1.12
**Eritrea**	0.29	0.13	0.16	1.04	0.76	0.88	1.04	1.10	1.07	0.19
**Estonia**	4.96	4.75	0.21	42.32	63.42	43.20	42.32	63.45	43.20	0.00
**Ethiopia**	0.73	0.60	0.13	9.21	26.14	12.28	9.25	26.42	12.36	0.08
**Falkland Islands**	3.96	2.97	1.00	0.00	0.00	0.00	0.57	0.91	0.65	0.65
**Fiji**	0.54	0.36	0.18	32.36	24.54	29.71	32.51	24.65	29.85	0.14
**Finland**	9.43	8.94	0.48	17.16	19.37	17.27	17.16	19.37	17.27	0.00
**France**	0.75	0.55	0.20	54.63	65.52	57.56	54.67	65.55	57.60	0.04
**French Guiana**	1.00	0.78	0.22	12.84	33.82	17.54	12.84	33.83	17.54	0.00
**French Polynesia**	12.13	7.85	4.28	0.00	0.00	0.00	0.05	0.07	0.06	0.06
**French Southern Territories**	7.49	4.92	2.57	90.02	93.18	91.10	90.02	93.18	91.10	0.00
**Gabon**	1.06	0.87	0.19	25.64	23.19	25.20	27.51	27.17	27.45	2.24
**Gambia**	3.23	1.70	1.53	4.73	18.16	11.09	4.73	18.16	11.09	0.00
**Georgia**	0.54	0.34	0.20	14.99	8.35	12.54	14.99	8.35	12.54	0.00
**Germany**	1.13	0.97	0.17	70.98	74.44	71.49	71.00	74.46	71.50	0.02
**Ghana**	2.62	2.24	0.38	4.76	12.56	5.88	4.84	14.29	6.20	0.31
**Gibraltar**	6.78	5.68	1.11	17.07	25.00	18.37	17.07	25.00	18.37	0.00
**Greece**	1.39	1.11	0.28	63.74	56.95	62.37	63.74	56.95	62.38	0.00
**Greenland**	1.74	1.67	0.07	21.63	12.20	21.22	21.78	12.70	21.39	0.16
**Grenada**	1.96	1.45	0.51	5.53	5.38	5.49	5.73	8.06	6.34	0.85
**Guadeloupe**	0.81	0.55	0.26	97.61	91.84	95.77	97.61	91.84	95.77	0.00
**Guam**	1.05	0.72	0.33	18.30	24.59	20.27	18.30	24.59	20.27	0.00
**Guatemala**	1.26	1.03	0.23	28.53	40.18	30.62	28.59	40.41	30.71	0.09
**Guernsey**	7.46	5.60	1.86	14.55	17.68	15.33	18.99	23.78	20.18	4.86
**Guinea**	0.37	0.10	0.27	6.21	7.15	6.90	10.20	42.45	33.71	26.81
**Guinea-Bissau**	2.59	1.17	1.42	20.48	21.61	21.10	20.48	21.61	21.10	0.00
**Guyana**	0.56	0.33	0.23	0.95	4.57	2.44	0.95	4.57	2.44	0.00
**Haiti**	1.11	0.85	0.26	0.54	0.31	0.49	0.54	0.34	0.50	0.01
**Honduras**	1.06	0.68	0.38	68.10	45.47	59.97	69.41	46.61	61.22	1.25
**Hong Kong**	5.30	3.99	1.32	44.10	18.86	37.83	45.95	24.01	40.50	2.68
**Hungary**	1.55	1.29	0.27	80.49	72.97	79.20	80.50	72.98	79.21	0.00
**Iceland**	2.05	2.03	0.02	12.66	8.74	12.62	12.79	9.26	12.75	0.13
**India**	2.30	0.59	1.70	18.16	26.16	24.09	21.82	27.41	25.97	1.88
**Indonesia**	1.30	0.67	0.62	12.28	9.28	10.84	12.44	9.48	11.01	0.18
**Iran**	0.70	0.19	0.51	100.00	51.70	64.99	100.00	52.61	65.65	0.66
**Iraq**	1.73	0.85	0.89	6.15	17.37	11.90	6.15	17.37	11.90	0.00
**Ireland**	1.98	1.71	0.27	62.10	65.27	62.54	62.11	65.29	62.55	0.01

**IW** = *inland permanent and seasonal water combined;* **IPW** = *inland permanent water;* **ISW** = *inland seasonal water*										
**Country name**	**% of country's land area that is IW**	**% of country's land area that is IPW**	**% of country's land area that is ISW**	**% of IPW that is protected**	**% of ISW that is protected**	**% of IW that is protected**	**% of IPW protected (including point buffers)**	**% of ISW protected (including point buffers)**	**% of IW protected (including point buffers)**	**Additional estimated percentage from buffers**
**Isle of Man**	1.15	0.76	0.39	0.00	0.00	0.00	0.00	0.00	0.00	0.00
**Israel**	2.28	2.12	0.17	0.61	2.33	0.73	2.83	2.52	2.81	2.07
**Italy**	1.03	0.82	0.21	50.09	46.55	49.38	50.09	46.55	49.38	0.00
**Jamaica**	0.49	0.32	0.18	59.01	48.50	55.22	59.01	48.50	55.22	0.00
**Japan**	1.19	0.93	0.26	68.12	35.50	61.03	68.13	35.52	61.04	0.01
**Jersey**	2.95	2.05	0.90	6.25	6.19	6.23	7.03	6.19	6.78	0.54
**Jordan**	0.53	0.50	0.04	0.09	0.22	0.10	0.12	0.51	0.15	0.04
**Kazakhstan**	3.27	1.83	1.44	5.23	4.49	4.90	16.74	7.03	12.46	7.56
**Kenya**	2.09	1.97	0.11	2.40	12.45	2.94	3.12	16.55	3.84	0.90
**Kiribati**	13.95	8.24	5.71	1.06	3.17	1.92	25.25	14.51	20.86	18.93
**Kosovo**	0.22	0.14	0.07	0.00	0.00	0.00	0.00	0.00	0.00	0.00
**Kuwait**	1.01	0.46	0.55	5.11	25.94	16.51	5.11	25.94	16.51	0.00
**Kyrgyzstan**	3.78	3.63	0.16	86.72	6.11	83.41	86.88	9.14	83.69	0.29
**Laos**	1.22	0.72	0.50	5.03	16.37	9.67	5.09	16.93	9.93	0.26
**Latvia**	1.88	1.43	0.45	38.49	43.26	39.63	38.49	43.26	39.63	0.00
**Lebanon**	0.32	0.24	0.08	1.66	0.65	1.42	1.66	0.65	1.42	0.00
**Lesotho**	0.30	0.16	0.14	0.00	0.02	0.01	0.00	0.02	0.01	0.00
**Liberia**	0.31	0.19	0.12	0.00	0.21	0.08	4.01	6.93	5.18	5.10
**Libya**	0.03	0.01	0.02	0.00	0.00	0.00	0.89	0.66	0.72	0.72
**Liechtenstein**	0.13	0.07	0.06	0.00	22.22	9.52	0.00	22.22	9.52	0.00
**Lithuania**	1.78	1.44	0.34	48.27	46.85	48.00	48.27	46.85	48.00	0.00
**Luxembourg**	0.22	0.15	0.06	49.37	72.67	56.07	49.62	72.67	56.25	0.18
**Macao**	12.57	8.38	4.19	0.00	0.00	0.00	0.00	0.00	0.00	0.00
**Macedonia**	2.09	1.99	0.10	89.92	25.98	86.84	89.92	25.98	86.84	0.00
**Madagascar**	1.15	0.44	0.71	1.87	0.58	1.07	12.71	6.19	8.70	7.62
**Malawi**	20.20	20.02	0.18	2.88	36.29	3.18	10.33	36.78	10.56	7.38
**Malaysia**	1.07	0.71	0.36	2.61	1.69	2.30	19.65	10.92	16.71	14.41
**Maldives**	28.45	22.29	6.17	0.89	1.83	1.10	0.89	1.83	1.10	0.00
**Mali**	0.43	0.11	0.32	0.33	2.60	2.02	19.20	37.36	32.71	30.69
**Malta**	3.02	2.65	0.37	43.52	60.83	45.63	43.52	60.83	45.63	0.00
**Marshall Islands**	7.41	2.56	4.85	19.50	15.79	17.07	19.50	15.79	17.07	0.00
**Martinique**	0.94	0.60	0.34	81.25	88.25	83.79	81.25	88.25	83.79	0.00
**Mauritania**	0.17	0.02	0.15	16.43	12.95	13.40	16.65	13.28	13.71	0.31
**Mauritius**	1.26	0.87	0.39	3.80	4.56	4.04	3.98	4.89	4.26	0.23
**Mayotte**	3.51	2.57	0.94	91.39	82.75	89.07	91.39	82.75	89.07	0.00
**Mexico**	1.06	0.53	0.53	15.84	29.05	22.47	24.33	36.12	30.25	7.78
**Micronesia**	1.71	1.04	0.67	0.00	0.00	0.00	5.03	3.94	4.60	4.60
**Moldova**	1.19	0.85	0.35	0.63	1.37	0.84	9.34	12.00	10.12	9.27
**Monaco**	7.22	7.22	0.00	100.00		100.00	100.00		100.00	0.00
**Mongolia**	1.01	0.89	0.12	75.49	26.37	69.63	79.95	26.71	73.60	3.97
**Montenegro**	2.52	1.86	0.67	0.88	0.33	0.73	55.63	15.74	45.09	44.36
**Montserrat**	1.30	0.46	0.84	0.00	0.00	0.00	0.00	0.00	0.00	0.00
**Morocco**	0.26	0.17	0.09	37.06	42.68	39.04	37.12	42.81	39.12	0.08
**Mozambique**	1.75	1.43	0.32	1.38	18.91	4.55	36.77	20.23	33.77	29.22
**Myanmar**	2.53	0.79	1.74	6.90	1.75	3.36	6.90	1.78	3.38	0.02
**Namibia**	0.28	0.05	0.24	46.89	85.69	78.95	46.89	85.69	78.95	0.00
**Nepal**	0.44	0.14	0.30	45.01	34.79	37.99	45.01	34.87	38.05	0.06
**Netherlands**	9.34	8.92	0.42	81.41	50.81	80.02	81.41	50.81	80.02	0.00
**New Caledonia**	1.00	0.63	0.38	24.28	24.01	24.18	51.81	48.47	50.56	26.38
**New Zealand**	2.06	1.80	0.26	31.96	26.56	31.28	31.97	26.60	31.29	0.02
**Nicaragua**	7.84	7.42	0.42	23.81	58.76	25.69	24.34	60.40	26.28	0.59

**IW** = *inland permanent and seasonal water combined;* **IPW** = *inland permanent water;* **ISW** = *inland seasonal water*										
**Country name**	**% of country's land area that is IW**	**% of country's land area that is IPW**	**% of country's land area that is ISW**	**% of IPW that is protected**	**% of ISW that is protected**	**% of IW that is protected**	**% of IPW protected (including point buffers)**	**% of ISW protected (including point buffers)**	**% of IW protected (including point buffers)**	**Additional estimated percentage from buffers**
**Niger**	0.13	0.03	0.09	2.42	2.03	2.14	75.19	60.03	64.13	62.00
**Nigeria**	0.96	0.41	0.55	6.92	12.68	10.21	6.96	12.79	10.29	0.08
**North Korea**	1.70	0.98	0.71	0.06	0.01	0.04	3.99	1.38	2.89	2.86
**Northern Cyprus**	0.46	0.30	0.16	0.00	0.00	0.00	0.00	0.00	0.00	0.00
**Northern Mariana Islands**	3.35	2.01	1.34	13.22	8.09	11.17	13.22	8.09	11.17	0.00
**Norway**	5.18	4.98	0.21	8.33	13.44	8.53	8.34	13.46	8.55	0.01
**Oman**	0.14	0.03	0.10	5.94	1.69	2.75	5.97	1.70	2.77	0.02
**Pakistan**	2.37	0.46	1.91	16.58	16.19	16.27	24.98	24.37	24.48	8.22
**Palau**	3.87	3.03	0.84	63.06	55.96	61.53	63.40	55.96	61.79	0.26
**Palestina**	3.20	3.11	0.09	0.00	0.00	0.00	0.00	0.00	0.00	0.00
**Panama**	1.43	1.03	0.40	14.71	18.15	15.67	15.14	18.98	16.21	0.54
**Papua New Guinea**	1.21	0.77	0.45	5.52	5.47	5.50	5.52	5.47	5.50	0.00
**Paraguay**	1.46	0.92	0.54	4.71	1.81	3.64	4.81	1.98	3.77	0.13
**Peru**	1.40	1.02	0.38	49.22	30.29	44.05	50.62	30.91	45.24	1.19
**Philippines**	1.86	1.22	0.64	24.19	6.46	18.06	24.22	6.76	18.19	0.12
**Poland**	1.34	1.11	0.23	75.16	77.47	75.55	75.16	77.48	75.56	0.00
**Portugal**	1.06	0.78	0.27	28.76	47.41	33.60	28.81	47.46	33.65	0.05
**Puerto Rico**	0.91	0.48	0.43	37.62	36.34	37.02	37.62	36.34	37.02	0.00
**Qatar**	0.63	0.27	0.36	0.00	0.00	0.00	3.41	4.20	3.87	3.87
**Republic of Congo**	0.91	0.62	0.29	2.50	5.10	3.32	40.65	59.02	46.49	43.16
**Reunion**	0.55	0.35	0.20	24.55	44.14	31.59	24.55	44.14	31.59	0.00
**Romania**	1.43	1.03	0.40	84.91	82.37	84.19	85.06	83.35	84.58	0.39
**Russia**	3.55	2.77	0.78	17.83	16.57	17.55	18.19	16.95	17.92	0.37
**Rwanda**	5.84	5.65	0.18	8.47	20.48	8.85	8.47	20.48	8.85	0.00
**Saint Helena**	2.96	0.67	2.30	5.51	68.88	54.65	5.51	68.88	54.65	0.00
**Saint Kitts and Nevis**	2.25	1.72	0.53	0.00	0.00	0.00	30.02	11.97	25.79	25.79
**Saint Lucia**	1.72	1.20	0.51	20.70	15.09	19.02	20.70	15.09	19.02	0.00
**Saint Pierre and Miquelon**	5.25	4.22	1.03	3.03	3.86	3.19	3.03	3.86	3.19	0.00
**Saint Vincent and the Grenadines**	2.23	1.65	0.58	14.39	13.85	14.25	14.39	13.85	14.25	0.00
**Saint-Barthelemy**	17.63	15.05	2.58	100.00	60.94	94.29	100.00	60.94	94.29	0.00
**Saint-Martin**	14.98	13.01	1.97	34.97	78.38	40.66	34.97	78.38	40.66	0.00
**Samoa**	0.66	0.46	0.20	0.04	0.05	0.04	14.42	10.46	13.23	13.19
**Saudi Arabia**	0.04	0.01	0.03	3.65	1.50	2.18	5.11	7.34	6.64	4.46
**Senegal**	1.88	0.61	1.27	17.06	10.27	12.47	17.08	10.27	12.48	0.01
**Serbia**	0.99	0.74	0.25	23.30	20.59	22.63	27.39	22.83	26.26	3.63
**Seychelles**	4.24	2.34	1.89	56.59	78.27	66.28	56.61	78.28	66.30	0.01
**Sierra Leone**	0.86	0.38	0.48	0.09	0.59	0.37	7.20	19.47	14.04	13.67
**Singapore**	6.94	3.38	3.56	18.54	8.25	13.26	18.54	8.25	13.26	0.00
**Sint Maarten**	13.84	10.60	3.24	0.49	0.00	0.38	0.49	0.00	0.38	0.00
**Slovakia**	0.57	0.47	0.10	56.95	50.46	55.84	56.95	50.46	55.84	0.00
**Slovenia**	0.37	0.21	0.16	64.14	93.54	77.07	64.14	93.54	77.07	0.00
**Solomon Islands**	1.64	1.32	0.32	45.56	15.62	39.72	45.70	15.81	39.87	0.14
**Somalia**	0.08	0.02	0.06	0.00	0.00	0.00	0.02	0.08	0.06	0.06
**South Africa**	0.43	0.29	0.15	43.25	24.83	37.00	43.25	24.83	37.00	0.00
**South Georgia / S Sandwich Is.**	6.33	4.09	2.24	100.00	100.00	100.00	100.00	100.00	100.00	0.00
**South Korea**	1.97	1.24	0.72	8.62	4.35	7.05	8.62	4.35	7.05	0.00
**South Sudan**	0.27	0.09	0.18	57.97	46.63	50.52	57.97	46.87	50.68	0.16
**Spain**	0.76	0.50	0.26	49.22	49.76	49.41	49.24	49.79	49.43	0.02
**Sri Lanka**	2.58	1.19	1.39	24.92	32.32	28.91	25.98	33.68	30.13	1.22
**Sudan**	0.28	0.15	0.14	0.58	1.34	0.94	0.76	2.36	1.52	0.58
**Suriname**	1.74	1.44	0.30	3.37	21.28	6.46	3.37	21.28	6.46	0.00

IW = *inland permanent and seasonal water combined*; IPW = *inland permanent water*; ISW = *inland seasonal water*										
**Country name**	**% of country's land area that is IW**	**% of country's land area that is IPW**	**% of country's land area that is ISW**	**% of IPW that is protected**	**% of ISW that is protected**	**% of IW that is protected**	**% of IPW protected (including point buffers)**	**% of ISW protected (including point buffers)**	**% of IW protected (including point buffers)**	**Additional estimated percentage from buffers**
**Swaziland**	0.39	0.27	0.12	0.15	0.34	0.21	0.15	0.34	0.21	0.00
**Sweden**	8.12	7.74	0.38	21.71	22.96	21.77	21.72	23.03	21.78	0.01
**Switzerland**	4.29	4.17	0.11	14.89	24.44	15.14	14.89	24.44	15.14	0.00
**Syria**	0.79	0.52	0.27	0.00	0.00	0.00	4.20	4.44	4.28	4.28
**Taiwan**	2.23	0.98	1.25	7.74	3.66	5.46	7.74	3.66	5.46	0.00
**Tajikistan**	1.52	1.06	0.46	49.59	16.21	39.49	61.34	32.94	52.75	13.26
**Tanzania**	6.18	5.83	0.35	9.72	45.85	11.77	11.18	45.94	13.15	1.39
**Thailand**	1.91	0.92	0.99	20.12	4.38	11.93	21.68	7.00	14.04	2.11
**Togo**	0.40	0.18	0.22	0.00	1.10	0.60	45.66	21.55	32.47	31.87
**Tokelau**	19.53	11.53	8.00	0.00	0.00	0.00	0.10	0.14	0.11	0.11
**Tonga**	5.06	3.75	1.32	1.57	2.87	1.91	32.76	21.28	29.77	27.86
**Trinidad and Tobago**	0.79	0.39	0.40	21.72	19.00	20.33	23.51	29.75	26.69	6.36
**Tunisia**	0.86	0.18	0.68	14.73	3.18	5.64	30.03	50.01	45.76	40.13
**Turkey**	1.82	1.39	0.43	4.72	6.13	5.05	4.72	6.14	5.06	0.00
**Turkmenistan**	5.73	4.76	0.96	5.50	3.44	5.15	5.60	4.30	5.38	0.24
**Turks and Caicos Islands**	38.23	17.27	20.96	45.99	63.71	55.71	45.99	63.71	55.71	0.00
**Tuvalu**	18.69	8.06	10.63	4.55	9.27	7.24	4.55	9.27	7.24	0.00
**Uganda**	15.06	14.83	0.23	0.72	8.49	0.84	2.27	16.40	2.49	1.65
**Ukraine**	2.49	2.13	0.36	10.15	13.68	10.66	12.35	18.87	13.29	2.64
**United Arab Emirates**	0.45	0.14	0.30	10.82	12.05	11.66	27.45	30.24	29.34	17.68
**United Kingdom**	1.36	1.17	0.19	47.21	55.86	48.42	47.21	55.86	48.42	0.00
**United States**	3.91	3.27	0.64	12.16	22.19	13.81	12.18	22.29	13.83	0.03
**United States Minor Outlying Is.**	0.92	0.77	0.15	26.67	0.00	22.30	26.67	0.00	22.30	0.00
**Uruguay**	2.86	1.96	0.90	34.70	18.32	29.55	35.20	18.55	29.97	0.42
**Uzbekistan**	4.06	2.02	2.04	0.40	0.35	0.37	12.10	2.17	7.10	6.73
**Vanuatu**	1.23	0.91	0.32	2.27	1.13	1.97	2.48	1.66	2.26	0.29
**Venezuela**	1.88	1.10	0.78	19.28	17.11	18.38	30.53	22.87	27.36	8.98
**Vietnam**	5.38	1.47	3.92	7.11	1.45	2.99	7.14	1.52	3.05	0.06
**Virgin Islands US**	3.76	2.68	1.08	28.48	24.62	27.37	28.48	24.62	27.37	0.00
**Wallis and Futuna**	2.51	1.47	1.03	0.00	0.00	0.00	0.00	0.00	0.00	0.00
**Western Sahara**	0.08	0.02	0.06	13.11	2.87	5.31	14.38	3.07	5.76	0.45
**Yemen**	0.08	0.04	0.04	9.26	3.50	6.32	10.03	3.80	6.85	0.53
**Zambia**	2.01	1.59	0.43	15.40	40.31	20.67	26.14	48.74	30.92	10.25
**Zimbabwe**	1.01	0.87	0.14	79.02	35.99	72.90	79.03	36.04	72.92	0.02
