# Supplementary material for: Inland surface waters in protected areas globally: Current coverage and 30-year trends
Source: PLoS One. 2019 Jan 17;14(1):e0210496. doi: 10.1371/journal.pone.0210496 (PMC6336238; doi:10.1371/journal.pone.0210496)
Supplement: S2 Table — (PDF) [file pone.0210496.s006.pdf]

IW = inland permanent and seasonal water combined; IPW = inland permanent water; ISW = inland seasonal water												
**Country name**	**% net change in IPW (protected)**	**% net change in ISW (protected)**	**% net change in IW (protected)**	**% net change in IPW (unprotected)**	**% net change in ISW (unprotected)**	**% net change in IW (unprotected)**	**Net area change of protected IPW (km2)**	**Net area change of protected ISW (km2)**	**Net area change of unprotected IPW (km2)**	**Net area change of unprotected ISW (km2)**	**Net total area change of IPW (km2)**	**Net total area change of ISW (km2)**
**Afghanistan**	-4.4	396.4	71.1	-56.6	43.2	-10.5	-3.0	63.7	-681.3	447.3	-684.3	511.0
**Akrotiri and Dhekelia**	0.0	0.0	0.0	26.7	102.1	83.6	0.0	0.0	0.5	5.4	0.5	5.4
**Aland**	-5.6	-13.7	-6.6	-8.8	-8.1	-8.7	-0.3	-0.1	-12.4	-1.6	-12.7	-1.7
**Albania**	-0.6	75.1	2.8	23.9	167.4	54.8	-2.6	15.7	26.2	50.3	23.6	66.0
**Algeria**	9.5	935.5	206.7	395.2	223.5	245.9	2.8	74.7	431.9	1623.9	434.7	1698.6
**American Samoa**	4.5	46.1	17.2	16.0	26.4	21.1	0.0	0.2	0.4	0.6	0.4	0.8
**Andorra**	-41.7	0.0	-38.5	7.8	50.0	12.3	-0.1	0.0	0.0	0.0	0.0	0.0
**Angola**	-19.5	151.1	141.7	0.6	77.5	35.9	-2.9	383.6	7.3	742.6	4.4	1126.2
**Anguilla**	0.0	0.0	0.0	-2.3	18.8	2.1	0.0	0.0	-0.1	0.2	-0.1	0.2
**Antigua and Barbuda**	8.9	69.7	18.4	-21.6	-8.0	-15.7	0.9	1.4	-0.9	-0.3	0.0	1.1
**Argentina**	-17.1	43.4	-1.3	-10.0	9.7	-0.6	-2037.5	1826.1	-2284.3	2041.1	-4321.9	3867.2
**Armenia**	0.0	-58.5	-0.4	87.8	12.0	53.1	0.5	-5.0	34.0	3.9	34.5	-1.1
**Aruba**	0.0	0.0	0.0	-9.0	-55.4	-35.1	0.0	0.0	-0.3	-2.2	-0.3	-2.2
**Australia**	-12.7	142.9	74.6	-3.1	146.2	86.4	-577.7	8303.3	-212.6	15166.7	-790.3	23470.0
**Austria**	-2.9	60.4	2.3	13.7	28.2	15.3	-11.1	20.4	26.2	6.9	15.1	27.4
**Azerbaijan**	6.6	54.4	34.6	-8.2	50.6	9.9	10.3	119.7	-79.1	218.3	-68.8	338.0
**Bahamas**	3.1	355.0	86.4	22.1	97.5	51.1	24.9	900.8	198.9	551.0	223.8	1451.8
**Bahrain**	0.0	0.0	0.0	-38.9	-22.2	-34.1	0.0	0.0	-7.4	-1.7	-7.4	-1.7
**Bangladesh**	43.7	17.1	28.5	27.4	-17.8	-12.4	35.1	18.4	743.9	-3596.9	779.0	-3578.6
**Barbados**	-50.0	-97.1	-94.4	-8.9	-84.4	-72.8	0.0	-0.3	-0.1	-6.3	-0.1	-6.6
**Belarus**	-7.6	14.2	-4.8	6.3	14.0	8.5	-33.4	9.0	81.4	70.7	48.0	79.7
**Belgium**	-6.0	95.5	4.7	29.8	109.3	39.8	-3.5	6.6	22.9	12.1	19.4	18.8
**Belize**	-5.4	29.4	4.8	4.7	-7.7	0.3	-6.6	15.0	11.8	-10.7	5.3	4.3
**Benin**	-5.6	39.7	38.3	4.0	209.9	36.7	0.0	2.2	11.2	110.5	11.2	112.6
**Bermuda**	4.4	33.3	6.2	3.9	90.6	6.4	0.0	0.0	0.4	0.3	0.4	0.3
**Bhutan**	-31.2	-12.4	-26.7	49.6	-8.3	27.5	-19.8	-2.5	18.1	-1.9	-1.7	-4.3
**Bolivia**	-18.0	26.4	-5.5	-23.9	-24.9	-24.3	-523.8	302.0	-2634.5	-2140.4	-3158.3	-1838.4
**Bonaire Saint Eustatius and Saba**	-5.6	36.4	0.1	20.0	3.5	17.6	-0.4	0.4	5.5	0.2	5.1	0.6
**Bosnia and Herzegovina**	-13.5	64.9	-3.9	11.8	34.1	18.3	-9.1	6.1	16.3	19.5	7.2	25.6
**Botswana**	625.5	97.4	129.6	316.4	546.9	526.5	253.2	607.0	84.1	1496.3	337.3	2103.3
**Brazil**	-6.2	30.7	4.8	9.8	27.5	13.8	-1325.0	2781.6	8407.0	7024.6	7082.1	9806.2
**British Indian Ocean Territory**	-6.3	110.1	18.2				-0.4	1.9	0.0	0.0	0.0	2.0
**British Virgin Islands**	-5.3	-17.1	-6.9	-1.8	2.5	-0.8	-0.1	-0.1	-0.3	0.1	-0.4	0.0
**Brunei**	6.4	80.3	21.1	7.9	38.0	15.9	0.5	1.6	2.4	4.2	2.9	5.7
**Bulgaria**	-11.4	14.2	-7.2	36.1	40.1	36.8	-45.5	11.2	101.8	23.8	56.4	35.0
**Burkina Faso**	1090.0	93.0	168.9	500.1	374.4	407.3	33.8	35.0	269.5	570.4	303.3	605.4
**Burundi**	-3.8	132.5	31.5	0.0	-4.0	0.0	-0.2	2.1	0.7	-0.5	0.5	1.6
**Cambodia**	-1.3	79.3	55.4	-6.0	14.0	5.5	-2.5	358.2	-208.3	665.0	-210.8	1023.2
**Cameroon**	-33.7	14.8	-15.1	5.0	55.0	20.9	-26.9	7.3	95.6	495.7	68.7	503.1
**Canada**	-7.5	37.7	-4.4	2.3	37.1	4.2	-5769.9	2158.2	21366.7	19614.7	15596.9	21772.9
**Cape Verde**	0.0	75.0	125.0	1.2	-44.6	-14.7	0.0	0.0	0.5	-10.6	0.6	-10.5
**Cayman Islands**	2.1	-44.4	-10.8	32.7	-66.1	-35.2	0.1	-0.8	2.1	-9.5	2.2	-10.3
**Central African Republic**	-61.8	8.5	-25.8	4.4	-3.8	0.7	-15.9	2.3	24.9	-17.4	9.0	-15.1
**Chad**	33.8	357.7	150.4	9.8	338.7	121.3	46.1	274.3	107.8	1915.4	153.8	2189.7
**Chile**	-13.4	21.3	-11.8	4.4	55.2	8.5	-326.4	24.5	470.1	514.3	143.7	538.8
**China**	6.8	39.9	11.7	20.2	33.0	25.1	2433.8	2478.7	13047.1	13068.9	15480.9	15547.6
**Colombia**	-26.4	9.8	-16.3	-2.4	8.2	1.5	-356.7	51.0	-177.3	354.4	-534.1	405.4
**Comoros**	0.0	0.0	0.0	-1.5	35.0	7.3	0.0	0.0	-0.3	2.1	-0.3	2.1
**Cook Islands**	0.0	2.6	1.0	1.5	-14.1	-10.2	0.0	0.0	0.0	-0.5	0.0	-0.5
**Costa Rica**	-12.8	4.8	-8.1	8.4	52.8	19.0	-7.5	1.0	14.3	28.4	6.8	29.5
**Cote dIvoire**	-9.3	19.3	-2.8	6.9	49.7	14.5	-17.8	10.9	100.7	153.7	82.9	164.6
**Croatia**	-14.0	51.9	2.7	126.2	110.8	124.0	-45.8	57.8	59.6	8.9	13.8	66.8
**Cuba**	10.0	100.0	33.6	8.2	104.6	35.8	51.2	181.1	99.4	511.4	150.6	692.5
**Curacao**	-7.0	-70.0	-40.4	-10.5	11.7	-4.1	-0.2	-2.3	-0.8	0.4	-1.0	-1.9
**Cyprus**	79.7	409.2	176.6	102.1	119.5	106.5	2.9	6.2	3.9	1.5	6.8	7.8
**Czech Republic**	-4.0	64.5	3.6	37.2	90.7	45.8	-7.8	15.7	86.0	40.2	78.2	55.9
**Democratic Republic of the Congo**	-4.4	-2.2	-4.2	-0.1	9.6	0.9	-111.8	-6.1	-45.3	368.7	-157.1	362.6
**Denmark**	1.9	47.3	7.7	49.0	59.8	50.4	9.4	33.4	32.9	5.7	42.3	39.1
**Djibouti**	0.0	0.0	0.0	6.7	46.8	21.0	0.0	0.0	8.8	34.0	8.8	34.0
**Dominica**	-50.0	-40.0	-47.1	-0.6	-35.1	-16.5	-0.1	0.0	0.0	-0.5	-0.1	-0.6
**Dominican Republic**	-6.5	188.7	3.8	116.0	100.6	111.6	-21.7	35.2	74.3	25.3	52.7	60.4

IW = *inland permanent and seasonal water combined;* IPW = *inland permanent water;* ISW = *inland seasonal water*												
**Country name**	**% net change in IPW (protected)**	**% net change in ISW (protected)**	**% net change in IW (protected)**	**% net change in IPW (unprotected)**	**% net change in ISW (unprotected)**	**% net change in IW (unprotected)**	**Net area change of protected IPW (km2)**	**Net area change of protected ISW (km2)**	**Net area change of unprotected IPW (km2)**	**Net area change of unprotected ISW (km2)**	**Net total area change of IPW (km2)**	**Net total area change of ISW (km2)**
**East Timor**	2.6	-51.8	-43.4	-9.8	278.2	87.9	0.1	-10.9	-1.5	21.9	-1.4	11.0
**Ecuador**	-12.5	4.9	-9.1	46.7	59.4	50.7	-43.6	4.1	726.9	417.2	683.3	421.3
**Egypt**	5.8	32.3	13.3	37.7	60.5	43.5	44.6	98.5	1595.4	876.5	1640.1	975.0
**El Salvador**	-12.7	57.3	11.2	0.8	136.7	16.0	-14.3	33.6	1.1	23.5	-13.2	57.1
**Equatorial Guinea**	7.1	4.3	6.8	1.0	17.3	5.9	5.7	0.4	0.2	1.9	5.9	2.3
**Eritrea**	0.0	1400.0	3010.0	22.2	78.8	48.5	1.6	1.4	27.8	86.3	29.5	87.7
**Estonia**	-4.6	-32.6	-6.9	2.8	-6.9	2.5	-43.6	-29.0	33.6	-2.6	-10.0	-31.5
**Ethiopia**	8.1	66.0	25.0	2.5	78.9	9.7	47.4	157.2	152.7	493.0	200.2	650.3
**Falkland Islands**	0.0	0.0	0.0	5.0	74.7	16.7	0.0	0.0	17.4	52.7	17.4	52.7
**Fiji**	11.8	10.0	11.3	14.1	23.0	17.2	2.3	0.8	5.7	4.9	8.0	5.7
**Finland**	-14.9	-11.4	-14.7	3.9	-10.5	3.1	-902.1	-40.4	937.8	-154.2	35.7	-194.6
**France**	-3.4	52.2	8.8	36.4	61.3	41.1	-57.9	249.2	364.0	145.3	306.1	394.5
**French Guiana**	-1.7	82.2	22.7	87.0	188.8	99.7	-1.5	28.7	264.7	81.4	263.2	110.0
**French Polynesia**	0.0	0.0	0.0	6.7	52.1	19.3	0.0	0.0	20.2	59.8	20.2	59.8
**French Southern Territories**	-1.0	-14.7	-6.3	0.2	5.6	1.6	-3.4	-32.5	0.1	0.7	-3.3	-31.8
**Gabon**	-14.6	11.2	-11.3	8.9	13.3	9.7	-102.3	11.8	141.1	45.6	38.8	57.4
**Gambia**	-1.4	400.3	161.2	-2.7	138.4	31.1	-0.1	23.9	-4.8	78.0	-5.0	101.9
**Georgia**	13.8	-0.7	9.8	10.1	36.9	19.1	4.3	-0.1	18.4	34.2	22.7	34.1
**Germany**	-5.3	37.5	-0.6	102.4	48.9	93.3	-137.1	119.9	506.6	49.5	369.5	169.4
**Ghana**	785.2	654.0	740.3	2.3	279.3	13.4	227.0	98.4	116.2	581.6	343.2	680.0
**Gibraltar**	-22.2	0.0	0.0	-15.0	50.0	-9.1	0.0	0.0	-0.1	0.0	-0.1	0.0
**Greece**	0.9	85.8	10.1	28.7	125.2	42.8	8.1	97.3	119.3	88.6	127.4	185.9
**Greenland**	-10.2	19.0	-9.7	4.0	9.2	4.2	-879.5	31.2	1074.4	118.5	194.9	149.7
**Grenada**	-12.1	-80.8	-54.1	3.8	-64.0	-30.5	0.0	-0.4	0.2	-3.1	0.1	-3.6
**Guadeloupe**	-11.5	-56.1	-32.5	0.0	-86.1	-77.4	-1.2	-5.0	0.2	-2.2	-0.3	-7.2
**Guam**	-14.1	36.4	0.0	0.0	53.3	11.5	-0.1	0.1	0.0	0.5	-0.1	0.6
**Guatemala**	-2.1	69.3	8.7	3.7	56.0	9.3	-6.9	40.7	28.6	53.1	21.7	93.8
**Guernsey**	-5.3	-32.6	-15.1	3.9	-15.6	-1.6	0.0	-0.1	0.2	-0.3	0.1	-0.4
**Guinea**	-13.1	65.8	35.8	1.6	63.9	40.4	-2.3	19.0	3.8	242.7	1.5	261.7
**Guinea-Bissau**	-33.0	29.2	-8.1	-2.9	76.6	28.7	-40.2	23.7	-9.5	165.0	-49.8	188.8
**Guyana**	71.9	6.1	16.3	10.4	-3.9	4.2	2.8	1.3	65.6	-18.9	68.4	-17.6
**Haiti**	231.6	633.3	261.0	8.2	84.5	19.7	0.9	0.2	17.5	32.1	18.3	32.3
**Honduras**	-4.2	74.3	9.2	47.7	88.5	65.2	-22.8	83.2	78.9	110.0	56.1	193.2
**Hong Kong**	-8.3	-2.4	-7.6	-9.6	-31.4	-18.0	-1.8	-0.1	-2.7	-5.6	-4.5	-5.6
**Hungary**	-1.7	-35.2	-9.1	74.5	28.0	61.4	-16.5	-98.0	99.5	14.6	83.0	-83.4
**Iceland**	-9.3	-14.3	-9.3	5.4	-20.1	5.0	-26.8	-0.3	91.7	-5.4	64.9	-5.7
**India**	3.4	346.9	171.2	20.1	13.8	15.5	113.4	10951.1	2579.8	4816.6	2693.2	15767.7
**Indonesia**	-3.9	40.5	10.5	3.3	82.4	31.1	-64.5	318.0	353.7	4867.8	289.2	5185.8
**Iran**	-40.7	96.9	-0.7	-100.0	15.8	-24.6	-2162.7	2108.0	-1854.8	546.6	-4017.5	2654.6
**Iraq**	-41.2	-10.5	-20.9	-33.0	-7.7	-22.8	-162.5	-80.9	-1742.4	-272.3	-1904.8	-353.2
**Ireland**	-22.8	36.4	-17.7	103.8	49.9	94.9	-219.7	33.2	231.6	22.0	11.9	55.2
**Isle of Man**	0.0	0.0	0.0	6.3	81.6	23.7	0.0	0.0	0.3	1.0	0.3	1.0
**Israel**	20.2	36.5	23.6	1.4	4.7	1.6	0.5	0.2	6.2	1.6	6.7	1.8
**Italy**	-3.1	36.9	2.5	18.5	-30.3	3.3	-40.0	77.6	193.3	-143.4	153.3	-65.9
**Jamaica**	4.6	-15.8	-2.9	10.7	-25.1	-7.7	0.9	-1.8	1.4	-3.4	2.3	-5.2
**Japan**	-3.2	19.9	-0.8	0.2	19.7	6.4	-77.2	56.7	2.0	102.3	-75.2	159.0
**Jersey**	33.3	-12.5	15.0	2.1	-8.6	-1.4	0.0	0.0	0.0	-0.1	0.1	-0.1
**Jordan**	1300.0	133.3	716.7	-0.8	29.3	0.8	0.4	0.0	-3.5	7.1	-3.1	7.1
**Kazakhstan**	0.5	50.3	16.0	-25.8	80.0	0.3	13.2	589.9	-16441.6	16668.0	-16428.5	17257.8
**Kenya**	8.8	-9.0	4.2	0.9	39.6	2.3	22.7	-8.2	105.3	164.2	127.9	156.1
**Kiribati**	89.1	-9.8	8.8	7.3	57.4	23.1	0.4	-0.2	5.6	20.5	6.0	20.3
**Kosovo**	0.0	0.0	0.0	-1.2	202.2	28.2	0.0	0.0	-0.2	5.4	-0.2	5.4
**Kuwait**	2.0	156.5	111.6	17.3	39.9	27.3	0.1	15.2	11.1	20.3	11.2	35.5
**Kyrgyzstan**	-1.6	72.1	-1.5	50.3	25.0	43.6	-101.0	7.9	320.9	58.0	219.9	65.9
**Laos**	265.4	653.0	467.9	16.9	128.4	43.4	60.9	163.7	228.5	542.2	289.4	705.9
**Latvia**	-15.2	-9.3	-13.7	10.5	7.7	9.8	-63.6	-12.9	53.8	11.8	-9.9	-1.1
**Lebanon**	28.1	-16.7	21.1	-1.9	-10.0	-4.0	0.1	0.0	-0.5	-0.8	-0.4	-0.9
**Lesotho**	0.0	-50.0	-50.0	305.7	-4.9	58.9	0.0	0.0	36.3	-2.2	36.3	-2.2
**Liberia**	0.0	92.3	92.3	0.5	41.0	13.5	0.0	0.1	0.9	35.0	0.9	35.1
**Libya**	0.0	0.0	0.0	12.3	6.7	8.1	0.0	0.0	13.2	20.9	13.2	20.9
**Liechtenstein**	0.0	0.0	0.0	-20.0	0.0	-13.6	0.0	0.0	0.0	0.0	0.0	0.0
**Lithuania**	-16.6	9.2	-12.7	24.7	21.9	24.1	-89.5	8.8	95.7	21.2	6.1	30.1

IW = *inland permanent and seasonal water combined;* **IPW** = *inland permanent water;* **ISW** = *inland seasonal water*

Country name	% net change in IPW (protected)	% net change in ISW (protected)	% net change in IW (protected)	% net change in IPW (unprotected)	% net change in ISW (unprotected)	% net change in IW (unprotected)	Net area change of protected IPW (km2)	Net area change of protected ISW (km2)	Net area change of unprotected IPW (km2)	Net area change of unprotected ISW (km2)	Net total area change of IPW (km2)	Net total area change of ISW (km2)
**Luxembourg**	-31.8	10.4	-20.5	45.3	76.0	50.0	-0.9	0.1	0.6	0.2	-0.3	0.3
**Macao**	0.0	0.0	0.0	-57.4	-64.5	-60.1	0.0	0.0	-3.8	-2.6	-3.8	-2.6
**Macedonia**	-3.5	19.0	-3.2	37.2	44.0	39.0	-16.0	1.0	13.5	5.7	-2.5	6.7
**Madagascar**	2.8	105.2	23.2	8.5	90.8	48.0	1.3	12.5	201.3	1984.7	202.6	1997.1
**Malawi**	-21.2	59.3	-16.9	1.4	30.1	1.5	-184.5	28.9	317.8	31.6	133.3	60.5
**Malaysia**	128.0	76.8	112.7	18.0	51.5	27.6	34.3	8.8	347.1	398.3	381.3	407.0
**Maldives**	-13.0	25.9	-2.1	-1.8	10.5	0.6	-0.1	0.1	-1.2	1.7	-1.3	1.8
**Mali**	684.7	688.4	688.2	29.2	35.9	34.1	4.0	91.4	312.6	1035.9	316.6	1127.3
**Malta**	-15.3	108.6	-6.3	11.2	88.0	15.3	-0.7	0.4	0.5	0.2	-0.2	0.6
**Marshall Islands**	2.3	29.4	17.2	11.5	40.2	29.1	0.0	0.5	0.6	3.5	0.7	4.1
**Martinique**	-15.5	-27.8	-20.6	0.0	-38.4	134.2	-1.0	-1.3	1.3	-0.3	0.8	-1.6
**Mauritania**	-1.7	244.3	147.3	13.6	562.8	315.4	-0.7	145.5	23.1	1169.8	22.4	1315.3
**Mauritius**	-45.7	65.8	-28.9	-9.1	34.7	1.0	-0.6	0.1	-1.7	2.0	-2.3	2.1
**Mayotte**	-3.0	293.6	19.4	0.0	128.6	439.3	-0.3	2.3	0.9	0.4	0.7	2.7
**Mexico**	1.5	60.4	33.3	5.7	89.7	32.7	23.8	1138.5	471.5	3492.3	495.3	4630.8
**Micronesia**	0.0	0.0	0.0	2.5	5.5	3.7	0.0	0.0	0.2	0.3	0.2	0.3
**Moldova**	-22.0	73.1	5.2	-11.3	85.7	4.5	-0.5	0.7	-36.5	53.6	-37.0	54.3
**Monaco**	-15.0	-100.0	-19.0	0.0	0.0	0.0	0.0	0.0	0.0	0.0	0.0	0.0
**Mongolia**	-2.4	77.2	-0.4	6.9	28.2	12.3	-258.5	216.1	219.9	304.9	-38.6	521.0
**Montenegro**	-3.1	38.1	0.4	-1.5	105.4	14.3	-0.1	0.1	-3.7	45.4	-3.8	45.5
**Montserrat**	0.0	0.0	0.0	-42.0	-12.4	-25.8	0.0	0.0	-0.3	-0.1	-0.3	-0.1
**Morocco**	117.9	154.0	130.5	146.6	254.7	174.2	140.6	98.3	262.3	156.3	402.8	254.6
**Mozambique**	-11.2	61.5	34.2	7.1	98.4	15.3	-19.8	180.5	743.6	1007.6	723.8	1188.1
**Myanmar**	25.3	60.4	36.0	33.7	87.9	67.5	74.1	77.2	1250.2	5383.6	1324.3	5460.8
**Namibia**	7.6	313.3	219.5	4.7	110.6	45.9	13.5	1264.8	9.7	146.3	23.2	1411.1
**Nepal**	-2.3	22.4	11.9	-3.0	-11.5	-9.3	-2.2	28.3	-3.5	-37.7	-5.7	-9.4
**Netherlands**	-1.9	64.3	-0.8	31.7	113.6	37.6	-53.7	31.8	150.1	41.8	96.4	73.5
**New Caledonia**	-6.9	64.0	10.9	4.6	66.9	21.7	-2.1	6.7	4.0	21.7	1.8	28.3
**New Zealand**	-0.2	11.2	0.9	-1.0	30.5	2.3	-3.6	18.8	-34.7	120.6	-38.4	139.4
**Nicaragua**	-3.0	136.5	4.6	1.8	32.7	2.5	-70.7	184.9	130.4	55.4	59.7	240.3
**Niger**	-0.9	109.9	56.3	209.4	353.1	302.6	-0.1	11.6	268.2	835.8	268.1	847.5
**Nigeria**	62.0	99.5	86.9	19.7	116.7	59.3	99.8	317.4	577.0	2360.5	676.8	2677.9
**North Korea**	-1.5	22.2	1.3	4.9	31.4	14.6	0.0	0.0	56.7	209.3	56.7	209.3
**Northern Cyprus**	0.0	0.0	0.0	34.3	28.5	32.2	0.0	0.0	2.6	1.2	2.6	1.2
**Northern Mariana Islands**	3.9	49.6	14.0	28.2	26.0	27.3	0.1	0.2	1.9	1.3	2.0	1.5
**Norway**	-29.0	-15.0	-28.3	5.3	-5.7	4.8	-549.2	-16.0	745.1	-35.5	195.9	-51.5
**Oman**	-1.6	294.9	50.1	-26.1	148.8	58.0	-0.1	4.0	-35.5	187.3	-35.6	191.3
**Pakistan**	4.8	424.0	191.9	19.7	163.8	113.7	30.8	2190.3	555.1	8698.8	585.9	10889.1
**Palau**	-7.5	123.3	4.6	19.3	67.6	28.5	-0.8	1.3	0.9	0.7	0.1	2.0
**Palestina**	0.0	0.0	0.0	-7.3	22.9	-6.7	0.0	0.0	-15.3	1.1	-15.3	1.1
**Panama**	1.3	37.8	10.7	7.3	21.4	10.8	1.5	14.9	45.2	43.2	46.7	58.1
**Papua New Guinea**	-5.8	32.6	5.3	12.6	138.5	39.7	-12.2	27.9	380.3	1140.9	368.1	1168.8
**Paraguay**	0.7	-25.7	-5.5	28.5	127.7	53.7	1.2	-13.6	784.1	1191.1	785.4	1177.5
**Peru**	-3.4	6.6	-1.7	13.6	51.0	24.1	-231.4	93.6	807.3	1170.0	575.9	1263.6
**Philippines**	5.5	24.4	7.6	0.0	41.4	13.1	45.9	24.2	0.6	522.7	46.6	546.9
**Poland**	-5.6	51.5	1.0	76.5	85.7	77.9	-154.3	186.5	373.1	73.6	218.8	260.1
**Portugal**	22.7	160.7	52.3	87.2	90.9	87.9	38.3	73.8	238.8	63.2	277.1	137.0
**Puerto Rico**	14.1	18.2	16.0	-3.0	-20.5	-12.2	2.0	2.2	-0.8	-6.3	1.2	-4.2
**Qatar**	0.0	0.0	0.0	-10.4	14.8	2.6	0.0	0.0	-3.6	5.5	-3.6	5.5
**Republic of Congo**	-13.7	25.4	1.8	-2.5	6.2	0.0	-8.4	10.3	-53.5	54.7	-61.9	64.9
**Reunion**	-4.3	69.5	22.4	0.3	99.3	17.4	-0.1	0.9	0.0	1.4	-0.1	2.3
**Romania**	-0.8	22.9	4.8	38.6	55.3	43.4	-16.0	146.7	102.6	60.0	86.6	206.7
**Russia**	-8.0	38.9	-1.0	6.4	25.7	10.2	-7185.7	6140.3	22949.8	22603.9	15764.1	28744.1
**Rwanda**	-19.5	-44.2	-22.0	3.3	110.5	4.8	-29.5	-7.5	42.7	19.3	13.2	11.8
**Saint Helena**	35.0	-0.4	0.2	125.5	-32.6	0.2	0.0	0.0	1.4	-1.4	1.5	-1.5
**Saint Kitts and Nevis**	0.0	0.0	0.0	-0.9	27.9	4.7	0.0	0.0	0.0	0.3	0.0	0.3
**Saint Lucia**	48.1	6.7	35.6	16.6	7.6	13.6	0.5	0.0	0.8	0.2	1.3	0.2
**Saint Pierre and Miquelon**	-3.3	50.0	5.6	1.0	65.9	9.3	0.0	0.0	0.1	0.9	0.1	0.9
**Saint Vincent and the Grenadines**	-13.6	-76.1	-48.0	1.6	-49.4	-19.5	-0.2	-1.0	0.1	-1.9	-0.1	-3.0
**Saint-Barthelemy**	-1.6	30.0	0.7	-100.0	-7.4	-19.4	-0.1	0.1	0.0	0.0	-0.1	0.1
**Saint-Martin**	-4.8	7.4	-2.0	1.1	-45.5	-2.9	-0.1	0.1	0.0	-0.2	-0.1	-0.1

IW = *inland permanent and seasonal water combined;* IPW = *inland permanent water;* ISW = *inland seasonal water*											
**Country name**	**% net change in IPW (protected)**	**% net change in ISW (protected)**	**% net change in IW (protected)**	**% net change in IPW (unprotected)**	**% net change in ISW (unprotected)**	**% net change in IW (unprotected)**	**Net area change of protected IPW (km2)**	**Net area change of protected ISW (km2)**	**Net area change of unprotected IPW (km2)**	**Net area change of unprotected ISW (km2)**	**Net total area change of IPW (km2)**	**Net total area change of ISW (km2)**
**Samoa**	0.0	0.0	0.0	1.9	51.6	13.0	0.0	0.0	0.3	1.9	0.3	1.9
**Saudi Arabia**	-15.9	-4.6	-10.9	32.3	75.9	59.6	-1.8	-0.4	62.0	244.5	60.2	244.0
**Senegal**	41.0	28.3	33.6	8.0	337.7	125.9	60.0	57.1	73.9	1745.3	133.9	1802.4
**Serbia**	-2.8	149.8	12.8	15.6	135.0	32.8	-3.9	23.7	60.2	87.5	56.4	111.1
**Seychelles**	38.0	7.6	20.1	10.5	-49.0	-17.3	1.8	0.5	0.5	-2.0	2.3	-1.4
**Sierra Leone**	-51.0	12.6	-1.3	-0.4	9.4	4.8	-0.3	0.2	-1.1	29.9	-1.4	30.2
**Singapore**	-27.4	114.6	-8.0	-50.9	29.4	-26.0	-1.7	1.1	-20.1	5.2	-21.7	6.3
**Sint Maarten**	0.0	0.0	0.0	-12.2	-28.3	-16.6	0.0	0.0	-0.6	-0.5	-0.6	-0.5
**Slovakia**	29.4	-24.4	16.5	40.3	51.1	42.2	30.0	-7.9	28.7	8.1	58.8	0.2
**Slovenia**	-8.1	19.4	4.8	204.1	101.9	186.2	-2.3	4.9	9.9	1.1	7.6	5.9
**Solomon Islands**	196.4	-88.8	0.7	6.5	73.9	19.1	114.8	-113.6	12.6	33.0	127.3	-80.5
**Somalia**	0.0	0.0	0.0	-16.1	-22.2	-20.6	0.0	0.0	-27.2	-105.2	-27.2	-105.2
**South Africa**	-3.1	149.7	12.6	49.1	124.4	72.5	-47.8	267.4	654.1	748.5	606.2	1015.9
**South Georgia / S. Sandwich Islands**	27.5	-28.3	-0.1				36.0	-36.2	0.0	0.0	36.0	-36.2
**South Korea**	-0.4	28.0	4.9	-3.2	1.0	-1.6	-0.4	6.9	-37.3	7.1	-37.8	14.0
**South Sudan**	-18.3	-21.1	-20.0	-15.8	8.4	0.0	-76.2	-140.2	-46.2	46.6	-122.4	-93.6
**Spain**	25.5	25.7	25.6	76.5	112.9	87.5	250.9	134.9	552.3	352.8	803.2	487.7
**Sri Lanka**	-25.6	110.0	21.8	39.1	44.9	42.1	-67.5	155.7	166.5	193.0	99.0	348.8
**Sudan**	1.8	78.1	43.3	26.1	50.8	36.7	0.3	15.0	577.4	847.9	577.7	862.9
**Suriname**	-18.0	5.0	-6.3	6.5	99.3	14.2	-15.7	4.5	125.2	174.0	109.5	178.5
**Swaziland**	16.7	-22.2	-6.7	40.5	141.1	60.7	0.0	0.0	13.7	12.0	13.7	12.0
**Sweden**	-17.2	-19.1	-17.3	7.4	-20.4	5.7	-1567.1	-92.6	1867.2	-337.5	300.2	-430.1
**Switzerland**	-8.0	19.0	-7.1	1.2	20.6	1.6	-22.3	1.8	18.0	6.0	-4.4	7.8
**Syria**	0.0	0.0	0.0	30.3	60.8	39.3	0.0	0.0	228.1	191.5	228.1	191.5
**Taiwan**	-8.3	21.4	1.0	-22.0	29.9	1.0	-2.5	3.0	-93.2	101.2	-95.7	104.1
**Tajikistan**	-0.1	115.6	7.0	6.0	25.3	13.3	-0.8	57.0	42.9	110.9	42.1	167.8
**Tanzania**	-11.0	32.6	-4.0	-0.5	91.9	1.2	-663.4	373.7	-258.2	858.9	-921.6	1232.7
**Thailand**	-7.6	29.5	-2.3	14.7	-5.8	2.1	-78.9	51.3	486.5	-304.0	407.6	-252.7
**Togo**	0.0	172.5	172.5	85.7	328.2	168.4	0.0	0.9	48.2	95.7	48.2	96.6
**Tokelau**	0.0	0.0	0.0	33.6	78.5	48.9	0.0	0.0	0.5	0.6	0.5	0.6
**Tonga**	-0.9	17.2	5.5	6.3	26.4	10.8	0.0	0.0	1.7	2.0	1.7	2.1
**Trinidad and Tobago**	-8.4	53.5	13.4	-1.9	-44.4	-29.7	-0.4	1.4	-0.3	-13.5	-0.7	-12.1
**Tunisia**	-41.7	181.8	-10.0	92.9	103.3	101.2	-29.9	21.6	116.4	516.6	86.5	538.1
**Turkey**	-11.7	11.9	-6.1	28.2	28.1	28.2	-68.0	21.8	2274.1	690.9	2206.1	712.6
**Turkmenistan**	51.0	141.8	57.6	248.7	58.0	188.9	433.5	95.4	15742.1	1675.4	16175.5	1770.7
**Turks and Caicos Islands**	-7.9	-2.4	-4.5	46.1	16.4	31.1	-6.8	-3.3	29.3	10.7	22.5	7.4
**Tuvalu**	-6.0	2.6	0.1	-1.6	18.7	8.8	0.0	0.0	-0.1	0.6	-0.1	0.6
**Uganda**	-12.2	-8.8	-11.7	-0.4	98.4	0.3	-35.9	-4.5	-137.1	251.6	-173.0	247.0
**Ukraine**	-3.4	-4.7	-3.6	1.1	31.8	4.5	-45.2	-14.6	124.0	450.9	78.8	436.3
**United Arab Emirates**	51.8	17.0	25.6	9.4	8.4	8.7	4.2	4.2	8.7	16.3	12.9	20.5
**United Kingdom**	-22.6	45.0	-16.3	39.4	56.6	41.3	-393.9	80.6	426.8	74.2	32.9	154.8
**United States**	-6.8	33.9	1.3	1.7	27.7	4.8	-2765.1	3405.0	4416.1	10236.3	1651.0	13641.3
**United States Minor Outlying Islands**	-15.3	77.1	-7.4				-0.1	0.0	0.0	0.0	-0.1	0.0
**Uruguay**	-3.9	-12.8	-5.8	8.4	72.9	25.5	-48.9	-43.1	177.5	550.4	128.6	507.3
**Uzbekistan**	-4.1	81.0	22.8	-59.8	47.5	-36.6	-1.6	14.2	-13380.6	2935.6	-13382.1	2949.8
**Vanuatu**	-28.2	40.6	-22.5	5.2	43.7	13.2	-1.0	0.1	5.4	11.9	4.4	12.1
**Venezuela**	-2.5	4.4	0.0	5.6	4.8	5.3	-50.6	51.0	435.2	270.0	384.5	321.0
**Vietnam**	7.9	5.7	7.1	32.4	32.9	32.7	25.2	10.1	1101.1	3153.0	1126.3	3163.1
**Virgin Islands US**	-11.7	-32.6	-18.3	-5.9	-40.0	-19.6	-0.4	-0.5	-0.4	-2.0	-0.8	-2.5
**Wallis and Futuna**	0.0	0.0	0.0	-0.4	26.6	9.2	0.0	0.0	0.0	0.3	0.0	0.3
**Western Sahara**	-4.4	442.5	44.7	21.6	163.9	110.2	-0.3	3.9	7.9	99.2	7.6	103.1
**Yemen**	-4.4	-11.9	-6.7	-6.0	65.3	21.6	-0.8	-0.9	-10.6	72.4	-11.4	71.5
**Zambia**	-18.0	63.4	3.2	-0.6	63.1	5.9	-405.2	503.2	-65.8	742.9	-471.0	1246.1
**Zimbabwe**	-1.6	293.0	3.9	330.8	169.4	258.6	-44.1	151.4	547.9	227.1	503.8	378.5
