# Supplementary material for: Inland surface waters in protected areas globally: Current coverage and 30-year trends
Source: PLoS One. 2019 Jan 17;14(1):e0210496. doi: 10.1371/journal.pone.0210496 (PMC6336238; doi:10.1371/journal.pone.0210496)
Supplement: S3 Table — This table also highlights the cases where trends are altered by including buffered points. (PDF) [file pone.0210496.s007.pdf]

Name	% net change in inland permanent water (protected)	% net change in inland seasonal water (protected)	% net change in all inland water (protected)	% net change in inland permanent water (unprotected)	% net change in inland seasonal water (unprotected)	% net change in all inland water (unprotected)	Permanent (+/-5% threshold)	Seasonal (+/-10% threshold)	Permanent (if different with buffers included)	Seasonal (if different with buffers included)
**Afghanistan**	-4.4	396.4	71.1	-56.6	43.2	-10.5	Loss outside PAs	Gain both inside and outside PAs		
**Akrotiri and Dhekelia**				26.7	102.1	83.6	Gain outside PAs, no water protected	Gain outside PAs, no water protected		
**Aland**	-5.6	-13.7	-6.6	-8.8	-8.1	-8.7	Loss both inside and outside PAs	Loss inside PAs		
**Albania**	-0.6	75.1	2.8	23.9	167.4	54.8	Gain outside PAs	Gain both inside and outside PAs		
**Algeria**	9.5	935.5	206.7	395.2	223.5	245.9	Gain both inside and outside PAs	Gain both inside and outside PAs		
**American Samoa**	4.5	46.1	17.2	16.0	26.4	21.1	Gain outside PAs	Gain both inside and outside PAs		
**Andorra**	-41.7	0.0	-38.5	7.8	50.0	12.3	Loss inside PAs, gain outside	Gain outside PAs		Loss inside PAs, gain outside
**Angola**	-19.5	151.1	141.7	0.6	77.5	35.9	Loss inside PAs	Gain both inside and outside PAs		
**Anguilla**				-2.3	18.8	2.1		Gain outside PAs, no water protected	Gain both inside and outside PAs	Gain outside PAs
**Antigua and Barbuda**	8.9	69.7	18.4	-21.6	-8.0	-15.7	Gain inside PAs, loss outside	Gain inside PAs		
**Argentina**	-17.1	43.4	-1.3	-10.0	9.7	-0.6	Loss both inside and outside PAs	Gain inside PAs		
**Armenia**	0.0	-58.5	-0.4	87.8	12.0	53.1	Gain outside PAs	Loss inside PAs, gain outside		
**Aruba**				-9.0	-55.4	-35.1	Loss outside PAs, no water protected	Loss outside PAs, no water protected		Loss both inside and outside PAs
**Australia**	-12.7	142.9	74.6	-3.1	146.2	86.4	Loss inside PAs	Gain both inside and outside PAs		
**Austria**	-2.9	60.4	2.3	13.7	28.2	15.3	Gain outside PAs	Gain both inside and outside PAs		
**Azerbaijan**	6.6	54.4	34.6	-8.2	50.6	9.9	Gain inside PAs, loss outside	Gain both inside and outside PAs		
**Bahamas**	3.1	355.0	86.4	22.1	97.5	51.1	Gain outside PAs	Gain both inside and outside PAs		
**Bahrain**				-38.9	-22.2	-34.1	Loss outside PAs, no water protected	Loss outside PAs, no water protected	Loss both inside and outside PAs	Loss both inside and outside PAs
**Bangladesh**	43.7	17.1	28.5	27.4	-17.8	-12.4	Gain both inside and outside PAs	Gain inside PAs, loss outside		Loss outside PAs
**Barbados**	-50.0	-97.1	-94.4	-8.9	-84.4	-72.8	Loss both inside and outside PAs	Loss both inside and outside PAs		
**Belarus**	-7.6	14.2	-4.8	6.3	14.0	8.5	Loss inside PAs, gain outside	Gain both inside and outside PAs		
**Belgium**	-6.0	95.5	4.7	29.8	109.3	39.8	Loss inside PAs, gain outside	Gain both inside and outside PAs		
**Belize**	-5.4	29.4	4.8	4.7	-7.7	0.3	Loss inside PAs	Gain inside PAs		
**Benin**	-5.6	39.7	38.3	4.0	209.9	36.7	Loss inside PAs	Gain both inside and outside PAs	Gain outside PAs	
**Bermuda**	4.4	33.3	6.2	3.9	90.6	6.4	Gain both inside and outside PAs	Gain both inside and outside PAs		
**Bhutan**	-31.2	-12.4	-26.7	49.6	-8.3	27.5	Loss inside PAs, gain outside	Loss inside PAs		
**Bolivia**	-18.0	26.4	-5.5	-23.9	-24.9	-24.3	Loss both inside and outside PAs	Gain inside PAs, loss outside		
**Bonaire Saint Eustatius and Sa**	-5.6	36.4	0.1	20.0	3.5	17.6	Loss inside PAs, gain outside	Gain inside PAs		
**Bosnia and Herzegovina**	-13.5	64.9	-3.9	11.8	34.1	18.3	Loss inside PAs, gain outside	Gain both inside and outside PAs		
**Botswana**	625.5	97.4	129.6	316.4	546.9	526.5	Gain both inside and outside PAs	Gain both inside and outside PAs		
**Brazil**	-6.2	30.7	4.8	9.8	27.5	13.8	Loss inside PAs, gain outside	Gain both inside and outside PAs		
**British Indian Ocean Territory**	0.0	110.1	18.2				Loss both inside and outside PAs	Gain inside PAs, loss outside		
**British Virgin Islands**	-5.3	-17.1	-6.9	-1.8	2.5	-0.8	Loss inside PAs	Loss inside PAs		Gain inside PAs
**Brunei**	6.4	80.3	21.1	7.9	38.0	15.9	Gain both inside and outside PAs	Gain both inside and outside PAs		
**Bulgaria**	-11.4	14.2	-7.2	36.1	40.1	36.8	Loss inside PAs, gain outside	Gain both inside and outside PAs		
**Burkina Faso**	1090.0	93.0	168.9	500.1	374.4	407.3	Gain both inside and outside PAs	Gain both inside and outside PAs		
**Burundi**	-3.8	132.5	31.5	0.0	-4.0	0.0	Gain both inside and outside PAs	Gain inside PAs	Loss inside PAs	
**Cambodia**	-1.3	79.3	55.4	-6.0	14.0	5.5	Loss outside PAs	Gain both inside and outside PAs		
**Cameroon**	-33.7	14.8	-15.1	5.0	55.0	20.9	Loss inside PAs	Gain both inside and outside PAs	Loss inside PAs, gain outside	
**Canada**	-7.5	37.7	-4.4	2.3	37.1	4.2	Loss inside PAs	Gain both inside and outside PAs		
**Cape Verde**		75.0	125.0	1.2	-44.6	-14.7		Gain inside PAs, loss outside		
**Cayman Islands**	2.1	-44.4	-10.8	32.7	-66.1	-35.2	Gain outside PAs	Loss both inside and outside PAs		
**Central African Republic**	-61.8	8.5	-25.8	4.4	-3.8	0.7	Loss inside PAs	Gain both inside and outside PAs		Gain inside PAs
**Chad**	33.8	357.7	150.4	9.8	338.7	121.3	Gain both inside and outside PAs	Gain both inside and outside PAs		
**Chile**	-13.4	21.3	-11.8	4.4	55.2	8.5	Loss inside PAs	Gain both inside and outside PAs		
**China**	6.8	39.9	11.7	20.2	33.0	25.1	Gain both inside and outside PAs	Gain both inside and outside PAs		
**Colombia**	-26.4	9.8	-16.3	-2.4	8.2	1.5	Loss inside PAs	Gain both inside and outside PAs		Gain inside PAs
**Comoros**				-1.5	35.0	7.3	Gain both inside and outside PAs	Gain both inside and outside PAs	Gain both inside and outside PAs	Gain outside PAs
**Cook Islands**	0.0	2.6	1.0	1.5	-14.1	-10.2	Gain both inside and outside PAs	Loss outside PAs		
**Costa Rica**	-12.8	4.8	-8.1	8.4	52.8	19.0	Loss inside PAs, gain outside	Gain outside PAs		Gain both inside and outside PAs
**Cote dIvoire**	-9.3	19.3	-2.8	6.9	49.7	14.5	Loss inside PAs, gain outside	Gain both inside and outside PAs		
**Croatia**	-14.0	51.9	2.7	126.2	110.8	124.0	Loss inside PAs, gain outside	Gain both inside and outside PAs		
**Cuba**	10.0	100.0	33.6	8.2	104.6	35.8	Gain both inside and outside PAs	Gain both inside and outside PAs		
**Curacao**	-7.0	-70.0	-40.4	-10.5	11.7	-4.1	Loss both inside and outside PAs	Loss inside PAs, gain outside		

Name	% net change in inland permanent water (protected)	% net change in inland seasonal water (protected)	% net change in all inland water (protected)	% net change in inland permanent water (unprotected)	% net change in inland seasonal water (unprotected)	% net change in all inland water (unprotected)	Permanent (+/-5% threshold)	Seasonal (+/-10% threshold)	Permanent (if different with buffers included)	Seasonal (if different with buffers included)
**Cyprus**	79.7	409.2	176.6	102.1	119.5	106.5	Gain both inside and outside PAs	Gain both inside and outside PAs		
**Czech Republic**	-4.0	64.5	3.6	37.2	90.7	45.8	Gain outside PAs	Gain both inside and outside PAs		
**Democratic Republic of the Co**	-4.4	-2.2	-4.2	-0.1	9.6	0.9	Gain both inside and outside PAs	Gain both inside and outside PAs	Loss inside PAs	Gain inside PAs
**Denmark**	1.9	47.3	7.7	49.0	59.8	50.4	Gain outside PAs	Gain both inside and outside PAs		
**Djibouti**				6.7	46.8	21.0	Gain outside PAs, no water protected	Gain both inside and outside PAs	Loss inside PAs, gain outside	Gain both inside and outside PAs
**Dominica**	-50.0	-40.0	-47.1	-0.6	-35.1	-16.5	Loss inside PAs	Loss both inside and outside PAs		
**Dominican Republic**	-6.5	188.7	3.8	116.0	100.6	111.6	Loss inside PAs, gain outside	Gain both inside and outside PAs		
**East Timor**	2.6	-51.8	-43.4	-9.8	278.2	87.9	Loss outside PAs	Loss inside PAs, gain outside		
**Ecuador**	-12.5	4.9	-9.1	46.7	59.4	50.7	Loss inside PAs, gain outside	Gain outside PAs		
**Egypt**	5.8	32.3	13.3	37.7	60.5	43.5	Gain both inside and outside PAs	Gain both inside and outside PAs		
**El Salvador**	-12.7	57.3	11.2	0.8	136.7	16.0	Loss inside PAs	Gain both inside and outside PAs		
**Equatorial Guinea**	7.1	4.3	6.8	1.0	17.3	5.9	Gain inside PAs	Gain outside PAs		
**Eritrea**		1400.0	3010.0	22.2	78.8	48.5	Gain outside PAs, no water protected	Gain both inside and outside PAs		
**Estonia**	-4.6	-32.6	-6.9	2.8	-6.9	2.5	Gain both inside and outside PAs	Loss inside PAs		
**Ethiopia**	8.1	66.0	25.0	2.5	78.9	9.7	Gain inside PAs	Gain both inside and outside PAs		
**Falkland Islands**				5.0	74.7	16.7		Gain outside PAs, no water protected	Loss inside PAs, gain outside	Gain both inside and outside PAs
**Fiji**	11.8	10.0	11.3	14.1	23.0	17.2	Gain both inside and outside PAs	Gain outside PAs		
**Finland**	-14.9	-11.4	-14.7	3.9	-10.5	3.1	Loss inside PAs	Loss both inside and outside PAs		
**France**	-3.4	52.2	8.8	36.4	61.3	41.1	Gain outside PAs	Gain both inside and outside PAs		
**French Guiana**	-1.7	82.2	22.7	87.0	188.8	99.7	Gain outside PAs	Gain both inside and outside PAs		
**French Polynesia**				6.7	52.1	19.3	Gain outside PAs, no water protected	Gain both inside and outside PAs	Gain outside PAs	Gain both inside and outside PAs
**French Southern Territories**	-1.0	-14.7	-6.3	0.2	5.6	1.6	Gain both inside and outside PAs	Loss inside PAs		
**Gabon**	-14.6	11.2	-11.3	8.9	13.3	9.7	Loss inside PAs, gain outside	Gain both inside and outside PAs		
**Gambia**	-1.4	400.3	161.2	-2.7	138.4	31.1	Gain both inside and outside PAs	Gain both inside and outside PAs		
**Georgia**	13.8	-0.7	9.8	10.1	36.9	19.1	Gain both inside and outside PAs	Gain outside PAs		
**Germany**	-5.3	37.5	-0.6	102.4	48.9	93.3	Loss inside PAs, gain outside	Gain both inside and outside PAs		
**Ghana**	785.2	654.0	740.3	2.3	279.3	13.4	Gain inside PAs	Gain both inside and outside PAs		
**Gibraltar**	-22.2		0.0	-15.0	50.0	-9.1	Loss both inside and outside PAs	Gain both inside and outside PAs		Gain outside PAs, no water protected
**Greece**	0.9	85.8	10.1	28.7	125.2	42.8	Gain outside PAs	Gain both inside and outside PAs		
**Greenland**	-10.2	19.0	-9.7	4.0	9.2	4.2	Loss inside PAs	Gain inside PAs		
**Grenada**	-12.1	-80.8	-54.1	3.8	-64.0	-30.5	Loss inside PAs	Loss both inside and outside PAs		
**Guadeloupe**	-5.8	-56.1	-32.5		-86.1	-77.4	Loss both inside and outside PAs	Loss both inside and outside PAs		
**Guam**	-14.1	36.4	0.0	0.0	53.3	11.5	Loss inside PAs	Gain both inside and outside PAs		
**Guatemala**	-2.1	69.3	8.7	3.7	56.0	9.3	Gain both inside and outside PAs	Gain both inside and outside PAs		
**Guernsey**	-5.3	-32.6	-15.1	3.9	-15.6	-1.6	Loss inside PAs	Loss both inside and outside PAs	Loss inside PAs, gain outside	
**Guinea**	-13.1	65.8	35.8	1.6	63.9	40.4	Loss inside PAs	Gain both inside and outside PAs	Loss inside PAs, gain outside	
**Guinea-Bissau**	-33.0	29.2	-8.1	-2.9	76.6	28.7	Loss inside PAs	Gain both inside and outside PAs		
**Guyana**	71.9	6.1	16.3	10.4	-3.9	4.2	Gain both inside and outside PAs	Gain both inside and outside PAs		
**Haiti**	231.6	633.3	261.0	8.2	84.5	19.7	Gain both inside and outside PAs	Gain both inside and outside PAs		
**Honduras**	-4.2	74.3	9.2	47.7	88.5	65.2	Gain outside PAs	Gain both inside and outside PAs		
**Hong Kong**	-8.3	-2.4	-7.6	-9.6	-31.4	-18.0	Loss both inside and outside PAs	Loss outside PAs		
**Hungary**	-1.7	-35.2	-9.1	74.5	28.0	61.4	Gain outside PAs	Loss inside PAs, gain outside		
**Iceland**	-9.3	-14.3	-9.3	5.4	-20.1	5.0	Loss inside PAs, gain outside	Loss both inside and outside PAs		
**India**	3.4	346.9	171.2	20.1	13.8	15.5	Gain outside PAs	Gain both inside and outside PAs		
**Indonesia**	-3.9	40.5	10.5	3.3	82.4	31.1	Gain both inside and outside PAs	Gain both inside and outside PAs		
**Iran**	-40.7	96.9	-0.7	-100.0	15.8	-24.6	Loss both inside and outside PAs	Gain both inside and outside PAs		
**Iraq**	-41.2	-10.5	-20.9	-33.0	-7.7	-22.8	Loss both inside and outside PAs	Loss inside PAs		
**Ireland**	-22.8	36.4	-17.7	103.8	49.9	94.9	Loss inside PAs, gain outside	Gain both inside and outside PAs		
**Isle of Man**				6.3	81.6	23.7	Gain outside PAs, no water protected	Gain both inside and outside PAs		
**Israel**	20.2	36.5	23.6	1.4	4.7	1.6	Gain inside PAs	Gain inside PAs	Gain both inside and outside PAs	
**Italy**	-3.1	36.9	2.5	18.5	-30.3	3.3	Gain outside PAs	Gain inside PAs, loss outside		
**Jamaica**	4.6	-15.8	-2.9	10.7	-25.1	-7.7	Gain outside PAs	Loss both inside and outside PAs		
**Japan**	-3.2	19.9	-0.8	0.2	19.7	6.4	Gain both inside and outside PAs	Gain both inside and outside PAs		
**Jersey**	33.3	-12.5	15.0	2.1	-8.6	-1.4	Gain inside PAs	Loss inside PAs		

Name	% net change in inland permanent water (protected)	% net change in inland seasonal water (protected)	% net change in all inland water (protected)	% net change in inland permanent water (unprotected)	% net change in inland seasonal water (unprotected)	% net change in all inland water (unprotected)	Permanent (+/-5% threshold)	Seasonal (+/-10% threshold)	Permanent (if different with buffers included)	Seasonal (if different with buffers included)
**Jordan**	1300.0	133.3	716.7	-0.8	29.3	0.8	Gain inside PAs	Gain both inside and outside PAs		
**Kazakhstan**	0.5	50.3	16.0	-25.8	80.0	0.3	Loss outside PAs	Gain both inside and outside PAs		
**Kenya**	8.8	-9.0	4.2	0.9	39.6	2.3	Gain inside PAs	Gain outside PAs		
**Kiribati**	89.1	-9.8	8.8	7.3	57.4	23.1	Gain both inside and outside PAs	Gain outside PAs	Gain inside PAs	Gain both inside and outside PAs
**Kosovo**				-1.2	202.2	28.2		Gain outside PAs, no water protected		Gain both inside and outside PAs
**Kuwait**	2.0	156.5	111.6	17.3	39.9	27.3	Gain outside PAs	Gain both inside and outside PAs		
**Kyrgyzstan**	-1.6	72.1	-1.5	50.3	25.0	43.6	Gain outside PAs	Gain both inside and outside PAs		
**Laos**	265.4	653.0	467.9	16.9	128.4	43.4	Gain both inside and outside PAs	Gain both inside and outside PAs		
**Latvia**	-15.2	-9.3	-13.7	10.5	7.7	9.8	Loss inside PAs, gain outside	Gain both inside and outside PAs		
**Lebanon**	28.1	-16.7	21.1	-1.9	-10.0	-4.0	Gain inside PAs	Loss inside PAs		
**Lesotho**		-50.0	-50.0	305.7	-4.9	58.9	Gain both inside and outside PAs	Loss inside PAs		
**Liberia**		92.3	92.3	0.5	41.0	13.5		Gain both inside and outside PAs	Loss inside PAs	
**Libya**				12.3	6.7	8.1	Gain outside PAs, no water protected		Gain outside PAs	Gain inside PAs
**Liechtenstein**		0.0	0.0	-20.0	0.0	-13.6	Loss outside PAs, no water protected	Gain both inside and outside PAs		
**Lithuania**	-16.6	9.2	-12.7	24.7	21.9	24.1	Loss inside PAs, gain outside	Gain outside PAs		
**Luxembourg**	-31.8	10.4	-20.5	45.3	76.0	50.0	Loss inside PAs, gain outside	Gain both inside and outside PAs		
**Macao**				-57.4	-64.5	-60.1	Loss outside PAs, no water protected	Loss outside PAs, no water protected		
**Macedonia**	-3.5	19.0	-3.2	37.2	44.0	39.0	Gain outside PAs	Gain both inside and outside PAs		
**Madagascar**	2.8	105.2	23.2	8.5	90.8	48.0	Gain outside PAs	Gain both inside and outside PAs		
**Malawi**	-21.2	59.3	-16.9	1.4	30.1	1.5	Loss inside PAs	Gain both inside and outside PAs		
**Malaysia**	128.0	76.8	112.7	18.0	51.5	27.6	Gain both inside and outside PAs	Gain both inside and outside PAs		
**Maldives**	-13.0	25.9	-2.1	-1.8	10.5	0.6	Loss inside PAs	Gain both inside and outside PAs		
**Mali**	684.7	688.4	688.2	29.2	35.9	34.1	Gain both inside and outside PAs	Gain both inside and outside PAs		
**Malta**	-15.3	108.6	-6.3	11.2	88.0	15.3	Loss inside PAs, gain outside	Gain both inside and outside PAs		
**Marshall Islands**	2.3	29.4	17.2	11.5	40.2	29.1	Gain outside PAs	Gain both inside and outside PAs		
**Martinique**	-8.2	-27.8	-20.6		-38.4	134.2	Loss both inside and outside PAs	Loss both inside and outside PAs		
**Mauritania**	-1.7	244.3	147.3	13.6	562.8	315.4	Gain outside PAs	Gain both inside and outside PAs		
**Mauritius**	-45.7	65.8	-28.9	-9.1	34.7	1.0	Loss both inside and outside PAs	Gain both inside and outside PAs		
**Mayotte**	-1.8	293.6	19.4		128.6	439.3	Loss outside PAs	Gain both inside and outside PAs		
**Mexico**	1.5	60.4	33.3	5.7	89.7	32.7	Gain outside PAs	Gain both inside and outside PAs		
**Micronesia**				2.5	5.5	3.7			Gain both inside and outside PAs	Loss inside PAs
**Moldova**	-22.0	73.1	5.2	-11.3	85.7	4.5	Loss both inside and outside PAs	Gain both inside and outside PAs		
**Monaco**	-10.5	-100.0	-19.0				Loss both inside and outside PAs	Gain both inside and outside PAs	Loss inside PAs	
**Mongolia**	-2.4	77.2	-0.4	6.9	28.2	12.3	Gain outside PAs	Gain both inside and outside PAs		
**Montenegro**	-3.1	38.1	0.4	-1.5	105.4	14.3	Gain both inside and outside PAs	Gain both inside and outside PAs		
**Montserrat**				-42.0	-12.4	-25.8	Loss outside PAs, no water protected	Loss outside PAs, no water protected		
**Morocco**	117.9	154.0	130.5	146.6	254.7	174.2	Gain both inside and outside PAs	Gain both inside and outside PAs		
**Mozambique**	-11.2	61.5	34.2	7.1	98.4	15.3	Loss inside PAs, gain outside	Gain both inside and outside PAs	Gain outside PAs	
**Myanmar**	25.3	60.4	36.0	33.7	87.9	67.5	Gain both inside and outside PAs	Gain both inside and outside PAs		
**Namibia**	7.6	313.3	219.5	4.7	110.6	45.9	Gain inside PAs	Gain both inside and outside PAs		
**Nepal**	-2.3	22.4	11.9	-3.0	-11.5	-9.3	Gain both inside and outside PAs	Gain inside PAs, loss outside		
**Netherlands**	-1.9	64.3	-0.8	31.7	113.6	37.6	Gain outside PAs	Gain both inside and outside PAs		
**New Caledonia**	-6.9	64.0	10.9	4.6	66.9	21.7	Loss inside PAs	Gain both inside and outside PAs	Loss inside PAs, gain outside	
**New Zealand**	-0.2	11.2	0.9	-1.0	30.5	2.3	Gain both inside and outside PAs	Gain both inside and outside PAs		
**Nicaragua**	-3.0	136.5	4.6	1.8	32.7	2.5	Gain both inside and outside PAs	Gain both inside and outside PAs		
**Niger**	-0.9	109.9	56.3	209.4	353.1	302.6	Gain outside PAs	Gain both inside and outside PAs	Gain inside PAs, loss outside	
**Nigeria**	62.0	99.5	86.9	19.7	116.7	59.3	Gain both inside and outside PAs	Gain both inside and outside PAs		
**North Korea**	-1.5	22.2	1.3	4.9	31.4	14.6	Gain both inside and outside PAs	Gain both inside and outside PAs	Gain outside PAs	Loss inside PAs, gain outside
**Northern Cyprus**				34.3	28.5	32.2	Gain outside PAs, no water protected	Gain outside PAs, no water protected		
**Northern Mariana Islands**	3.9	49.6	14.0	28.2	26.0	27.3	Gain outside PAs	Gain both inside and outside PAs		
**Norway**	-29.0	-15.0	-28.3	5.3	-5.7	4.8	Loss inside PAs, gain outside	Loss inside PAs		
**Oman**	-1.6	294.9	50.1	-26.1	148.8	58.0	Loss outside PAs	Gain both inside and outside PAs		
**Pakistan**	4.8	424.0	191.9	19.7	163.8	113.7	Gain outside PAs	Gain both inside and outside PAs		
**Palau**	-7.5	123.3	4.6	19.3	67.6	28.5	Loss inside PAs, gain outside	Gain both inside and outside PAs		

Name	% net change in inland permanent water (protected)	% net change in inland seasonal water (protected)	% net change in all inland water (protected)	% net change in inland permanent water (unprotected)	% net change in inland seasonal water (unprotected)	% net change in all inland water (unprotected)	Permanent (+/-5% threshold)	Seasonal (+/-10% threshold)	Permanent (if different with buffers included)	Seasonal (if different with buffers included)
Palestina				-7.3	22.9	-6.7	Loss outside PAs, no water protected	Gain outside PAs, no water protected		
Panama	1.3	37.8	10.7	7.3	21.4	10.8	Gain outside PAs	Gain both inside and outside PAs		
Papua New Guinea	-5.8	32.6	5.3	12.6	138.5	39.7	Loss inside PAs, gain outside	Gain both inside and outside PAs		
Paraguay	0.7	-25.7	-5.5	28.5	127.7	53.7	Gain outside PAs	Loss inside PAs, gain outside		
Peru	-3.4	6.6	-1.7	13.6	51.0	24.1	Gain outside PAs	Gain outside PAs		
Philippines	5.5	24.4	7.6	0.0	41.4	13.1	Gain inside PAs	Gain both inside and outside PAs		
Poland	-5.6	51.5	1.0	76.5	85.7	77.9	Loss inside PAs, gain outside	Gain both inside and outside PAs		
Portugal	22.7	160.7	52.3	87.2	90.9	87.9	Gain both inside and outside PAs	Gain both inside and outside PAs		
Puerto Rico	14.1	18.2	16.0	-3.0	-20.5	-12.2	Gain inside PAs	Gain inside PAs, loss outside		
Qatar				-10.4	14.8	2.6	Loss outside PAs, no water protected	Gain outside PAs, no water protected	Loss both inside and outside PAs	Gain both inside and outside PAs
Republic of Congo	-13.7	25.4	1.8	-2.5	6.2	0.0	Loss inside PAs	Gain inside PAs	Loss inside PAs, gain outside	Gain outside PAs
Reunion	-4.3	69.5	22.4	0.3	99.3	17.4	Gain both inside and outside PAs	Gain both inside and outside PAs		
Romania	-0.8	22.9	4.8	38.6	55.3	43.4	Gain outside PAs	Gain both inside and outside PAs		
Russia	-8.0	38.9	-1.0	6.4	25.7	10.2	Loss inside PAs, gain outside	Gain both inside and outside PAs		
Rwanda	-19.5	-44.2	-22.0	3.3	110.5	4.8	Loss inside PAs	Loss inside PAs, gain outside		
Saint Helena	35.0	-0.4	0.2	125.5	-32.6	0.2	Gain both inside and outside PAs	Loss outside PAs		
Saint Kitts and Nevis				-0.9	27.9	4.7		Gain outside PAs, no water protected	Loss inside PAs	Gain both inside and outside PAs
Saint Lucia	48.1	6.7	35.6	16.6	7.6	13.6	Gain both inside and outside PAs	Gain both inside and outside PAs		
Saint Pierre and Miquelon	-3.3	50.0	5.6	1.0	65.9	9.3	Gain both inside and outside PAs	Gain both inside and outside PAs		
Saint Vincent and the Grenadi	-13.6	-76.1	-48.0	1.6	-49.4	-19.5	Loss inside PAs	Loss both inside and outside PAs		
Saint-Barthelemy	-1.6	30.0	0.7	-100.0	-7.4	-19.4	Loss outside PAs	Gain inside PAs		
Saint-Martin	-4.8	7.4	-2.0	1.1	-45.5	-2.9	Gain both inside and outside PAs	Loss outside PAs		
Samoa	0.0	0.0	0.0	1.9	51.6	13.0	Gain both inside and outside PAs	Gain outside PAs	Gain inside PAs	Gain both inside and outside PAs
Saudi Arabia	-15.9	-4.6	-10.9	32.3	75.9	59.6	Loss inside PAs, gain outside	Gain outside PAs		Gain both inside and outside PAs
Senegal	41.0	28.3	33.6	8.0	337.7	125.9	Gain both inside and outside PAs	Gain both inside and outside PAs		
Serbia	-2.8	149.8	12.8	15.6	135.0	32.8	Gain outside PAs	Gain both inside and outside PAs		
Seychelles	38.0	7.6	20.1	10.5	-49.0	-17.3	Gain both inside and outside PAs	Loss outside PAs		
Sierra Leone	-51.0	12.6	-1.3	-0.4	9.4	4.8	Loss inside PAs	Gain inside PAs		Loss inside PAs, gain outside
Singapore	-27.4	114.6	-8.0	-50.9	29.4	-26.0	Loss both inside and outside PAs	Gain both inside and outside PAs		
Sint Maarten	0.0		0.0	-12.2	-28.3	-16.6	Loss outside PAs	Loss outside PAs, no water protected		
Slovakia	29.4	-24.4	16.5	40.3	51.1	42.2	Gain both inside and outside PAs	Loss inside PAs, gain outside		
Slovenia	-8.1	19.4	4.8	204.1	101.9	186.2	Loss inside PAs, gain outside	Gain both inside and outside PAs		
Solomon Islands	196.4	-88.8	0.7	6.5	73.9	19.1	Gain both inside and outside PAs	Loss inside PAs, gain outside		
Somalia				-16.1	-22.2	-20.6	Loss outside PAs, no water protected	Loss outside PAs, no water protected	Loss outside PAs	Loss outside PAs
South Africa	-3.1	149.7	12.6	49.1	124.4	72.5	Gain outside PAs	Gain both inside and outside PAs		
South Georgia and the South S	27.5	-28.3	-0.1				Gain inside PAs	Gain both inside and outside PAs		
South Korea	-0.4	28.0	4.9	-3.2	1.0	-1.6	Gain both inside and outside PAs	Gain inside PAs		
South Sudan	-18.3	-21.1	-20.0	-15.8	8.4	0.0	Loss both inside and outside PAs	Loss inside PAs		
Spain	25.5	25.7	25.6	76.5	112.9	87.5	Gain both inside and outside PAs	Gain both inside and outside PAs		
Sri Lanka	-25.6	110.0	21.8	39.1	44.9	42.1	Loss inside PAs, gain outside	Gain both inside and outside PAs		
Sudan	1.8	78.1	43.3	26.1	50.8	36.7	Gain outside PAs	Gain both inside and outside PAs	Loss inside PAs, gain outside	
Suriname	-18.0	5.0	-6.3	6.5	99.3	14.2	Loss inside PAs, gain outside	Gain outside PAs		
Swaziland	16.7	-22.2	-6.7	40.5	141.1	60.7	Gain both inside and outside PAs	Loss inside PAs, gain outside		
Sweden	-17.2	-19.1	-17.3	7.4	-20.4	5.7	Loss inside PAs, gain outside	Loss both inside and outside PAs		
Switzerland	-8.0	19.0	-7.1	1.2	20.6	1.6	Loss inside PAs	Gain both inside and outside PAs		
Syria				30.3	60.8	39.3	Gain outside PAs, no water protected	Gain outside PAs, no water protected	Gain both inside and outside PAs	Gain both inside and outside PAs
Taiwan	-8.3	21.4	1.0	-22.0	29.9	1.0	Loss both inside and outside PAs	Gain both inside and outside PAs		
Tajikistan	-0.1	115.6	7.0	6.0	25.3	13.3	Gain outside PAs	Gain both inside and outside PAs		
Tanzania	-11.0	32.6	-4.0	-0.5	91.9	1.2	Loss inside PAs	Gain both inside and outside PAs		
Thailand	-7.6	29.5	-2.3	14.7	-5.8	2.1	Loss inside PAs, gain outside	Gain inside PAs		Gain both inside and outside PAs
Togo		172.5	172.5	85.7	328.2	168.4	Gain outside PAs, no water protected	Gain both inside and outside PAs	Loss inside PAs, gain outside	
Tokelau				33.6	78.5	48.9	Gain outside PAs, no water protected	Gain outside PAs, no water protected		
Tonga	-0.9	17.2	5.5	6.3	26.4	10.8	Gain outside PAs	Gain both inside and outside PAs	Gain both inside and outside PAs	Gain outside PAs
Trinidad and Tobago	-8.4	53.5	13.4	-1.9	-44.4	-29.7	Loss inside PAs	Gain inside PAs, loss outside		Loss both inside and outside PAs

Name	% net change in inland permanent water (protected)	% net change in inland seasonal water (protected)	% net change in all inland water (protected)	% net change in inland permanent water (unprotected)	% net change in inland seasonal water (unprotected)	% net change in all inland water (unprotected)	Permanent (+/-5% threshold)	Seasonal (+/-10% threshold)	Permanent (if different with buffers included)	Seasonal (if different with buffers included)
**Tunisia**	-41.7	181.8	-10.0	92.9	103.3	101.2	Loss inside PAs, gain outside	Gain both inside and outside PAs		
**Turkey**	-11.7	11.9	-6.1	28.2	28.1	28.2	Loss inside PAs, gain outside	Gain both inside and outside PAs		
**Turkmenistan**	51.0	141.8	57.6	248.7	58.0	188.9	Gain both inside and outside PAs	Gain both inside and outside PAs		
**Turks and Caicos Islands**	-7.9	-2.4	-4.5	46.1	16.4	31.1	Loss inside PAs, gain outside	Gain outside PAs		
**Tuvalu**	-6.0	2.6	0.1	-1.6	18.7	8.8	Loss inside PAs	Gain outside PAs		
**Uganda**	-12.2	-8.8	-11.7	-0.4	98.4	0.3	Loss inside PAs	Gain outside PAs		Gain both inside and outside PAs
**Ukraine**	-3.4	-4.7	-3.6	1.1	31.8	4.5	Gain both inside and outside PAs	Gain outside PAs		
**United Arab Emirates**	51.8	17.0	25.6	9.4	8.4	8.7	Gain both inside and outside PAs	Gain inside PAs	Gain inside PAs	
**United Kingdom**	-22.6	45.0	-16.3	39.4	56.6	41.3	Loss inside PAs, gain outside	Gain both inside and outside PAs		
**United States**	-6.8	33.9	1.3	1.7	27.7	4.8	Loss inside PAs	Gain both inside and outside PAs		
**United States Minor Outlying I**	0.0		0.0	-19.8	77.1	-9.3	Loss outside PAs	Gain outside PAs, no water protected		
**Uruguay**	-3.9	-12.8	-5.8	8.4	72.9	25.5	Gain outside PAs	Loss inside PAs, gain outside		
**Uzbekistan**	-4.1	81.0	22.8	-59.8	47.5	-36.6	Loss outside PAs	Gain both inside and outside PAs	Gain inside PAs, loss outside	
**Vanuatu**	-28.2	40.6	-22.5	5.2	43.7	13.2	Loss inside PAs, gain outside	Gain both inside and outside PAs		
**Venezuela**	-2.5	4.4	0.0	5.6	4.8	5.3	Gain outside PAs	Gain both inside and outside PAs		
**Vietnam**	7.9	5.7	7.1	32.4	32.9	32.7	Gain both inside and outside PAs	Gain outside PAs		
**Virgin Islands US**	-11.7	-32.6	-18.3	-5.9	-40.0	-19.6	Loss both inside and outside PAs	Loss both inside and outside PAs		
**Wallis and Futuna**				-0.4	26.6	9.2		Gain outside PAs, no water protected		
**Western Sahara**	-4.4	442.5	44.7	21.6	163.9	110.2	Gain outside PAs	Gain both inside and outside PAs		
**Yemen**	-4.4	-11.9	-6.7	-6.0	65.3	21.6	Loss outside PAs	Loss inside PAs, gain outside	Loss both inside and outside PAs	
**Zambia**	-18.0	63.4	3.2	-0.6	63.1	5.9	Loss inside PAs	Gain both inside and outside PAs		
**Zimbabwe**	-1.6	293.0	3.9	330.8	169.4	258.6	Gain outside PAs	Gain both inside and outside PAs		
